# Efficient Self-Immolative RAFT End Group Modification for Macromolecular Immunodrug Delivery

**DOI:** 10.1021/acs.biomac.3c00239

**Published:** 2023-04-24

**Authors:** Maximilian Scherger, Yannick A. Pilger, Judith Stickdorn, Patric Komforth, Sascha Schmitt, Kaloian Koynov, Hans Joachim Räder, Lutz Nuhn

**Affiliations:** † Max Planck Institute for Polymer Research, Ackermannweg 10, 55128 Mainz, Germany; ‡ Chair of Macromolecular Chemistry, Julius-Maximilians-University Würzburg, Röntgenring 11, 97070 Würzburg, Germany

## Abstract

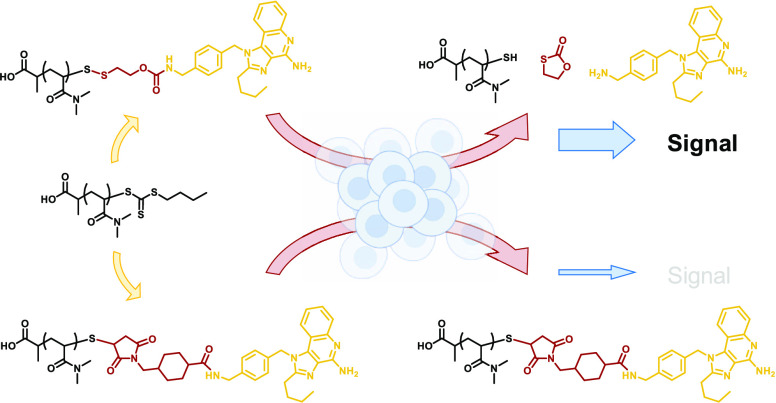

The reversible addition–fragmentation chain-transfer (RAFT) polymerization provides access to a broad variety of biocompatible and functional macromolecules for diverse polymer–drug conjugates. Due to thiocarbonylthio groups at the ends of each growing polymer chain, they can straightforwardly be converted into disufilde-containing self-immolative motives for reversible drug conjugation by traceless linkers. This may be relevant for RAFT-polymerized poly(*N*,*N*-dimethylacrylamide) (pDMA), which has been demonstrated to provide similar properties as poly(ethylene glycol) (PEG) in terms of improving the drug’s poor pharmacokinetic profile or enhancing its bioavailability. For that purpose, we established a highly efficient one-pot reaction procedure for introducing various functionalities including both primary and secondary amines and primary alcohols and demonstrated their reversible conjugation and traceless release from pDMA’s polymer chain end. Next, a first polymer–drug conjugate with a Toll-like receptor agonist exhibited significantly increased activity *in vitro* compared to conventional irreversibly covalently fixed variants. Finally, α-ω-bifunctional dye or drug conjugates could be generated by a cholesterol-modified RAFT chain-transfer agent. It facilitated the polymer–drug conjugate’s internalization at the cellular level monitored by flow cytometry and confocal imaging. This approach provides the basis for a variety of potentially impactful polymer–drug conjugates by combining versatile small molecular drugs with a plethora of available RAFT polymers through reductive-responsive self-immolative linkers.

## Introduction

Many potentially very impactful drugs possess a poor pharmacokinetic profile and thus low bioavailability, which is why the formulation of polymer–drug conjugates using poly(ethylene glycol) (PEG) has become a typical approach to counteract this tendency.^1^ However, recently, the trend has moved away again from these forms of PEGylations due to the oxidative instability of these systems and their increasing potential to cause immune reactions.^2^ Some other clinically studied or even FDA-approved polymers such as poly(2-hydroxyethyl methacrylate) (pHEMA), poly(vinylpyrrolidone) (PVP), poly(*N*-(2-hydroxypropyl)methacrylamide) (pHPMA), and poly(*N*,*N*-dimethylacrylamide) (pDMA) are considered as possible vinyl-type alternatives. While pHEMA or pHPMA can in principle be modified by cumbersome esterification of the hydroxy functionality in the side chain, all other polymers lack additional side chain functionalities for drug conjugation, which makes the formulation of drug–polymer conjugates nontrivial.

The aforementioned polymers are, however, accessible via controlled reversible addition–fragmentation chain-transfer (RAFT) polymerization that enables straightforward controlled homo and block copolymerization as well as the possibility of controlling α- and ω-functionalities. On the one hand, the α-end can be adapted, for example, by attaching the drug to the chain-transfer agent (CTA) prior to polymerization^3,4^ or by post-polymerization modifications exploiting, for instance, pentafluorophenyl ester,^5^*N*-hydroxysuccinimide ester,^6^ squaric acid ester,^6^ or azide functionalized CTAs.^7^

The ω-end, on the other hand, bears a thiocarbonylthio moiety, for which studies attribute only very low to no cytotoxicity at all,^8^ but it is often contemplated less beneficial, for example, due to its high reactivity toward nucleophiles, which should be considered especially in the context of biomedical applications. Accordingly, this group can either be completely replaced or removed by using excess of radical starters,^9^ thermolysis,^10^ UV irradiation,^11^ or radical-induced reduction,^12^ or it can be exploited to introduce functionalities in various ways. For instance, the thiocarbonyl moiety can undergo hetero-Diels–Alder reactions with dienes,^13^ but due to its susceptibility to aminolysis and associated revelation of thiols, the use of thiol click reactions is also widespread. In addition to Michael additions,^14^ reactions with maleimides,^15^ vinyl sulfones,^16^ and isocyanates,^17^ the sulfhydryl group can be trapped with 2,2′-dithiodipyridine and the polymer subsequently used for biofunctionalizations via disulfide exchange reactions.^18^ A similar principle underlies when the terminal thiol is reacted with other thiols activated by methanethiosulfonates.^19−21^

In the latter two variants, disulfides are generated at the polymer end, which can correspondingly be cleaved reductively, thus making modifications reversible. This property is of particular interest in a biomedical context, as it creates systems that retain their integrity in an extracellular environment but can be degraded intracellularly at their site of action due to the overall reductive environment caused by the abundant presence of glutathione (GSH) and, therefore, release their cargo in a controlled manner.

However, these systems are usually limited by the fact that the active compound must contain a free thiol. Alternatively, it requires an additional implementation of such, which in turn can compromise its bioactivity. In contrast, other functionalities such as alcohols or amines are much more widespread in many drug classes, making reductive-responsive self-immolative linkers (SILs) capable of bridging thiol-free drugs with disulfide chemistry an attractive tool.^22^ These can be conjugated to alcohols or amines, respectively, as carbonates or carbamates in the β-position to the disulfide. When the disulfide is reduced by an external trigger, the previously introduced moiety is released again without traces in an intramolecular 5-exo-trig or 3-exo-tet cyclization.^22,23^

**Figure 1:**
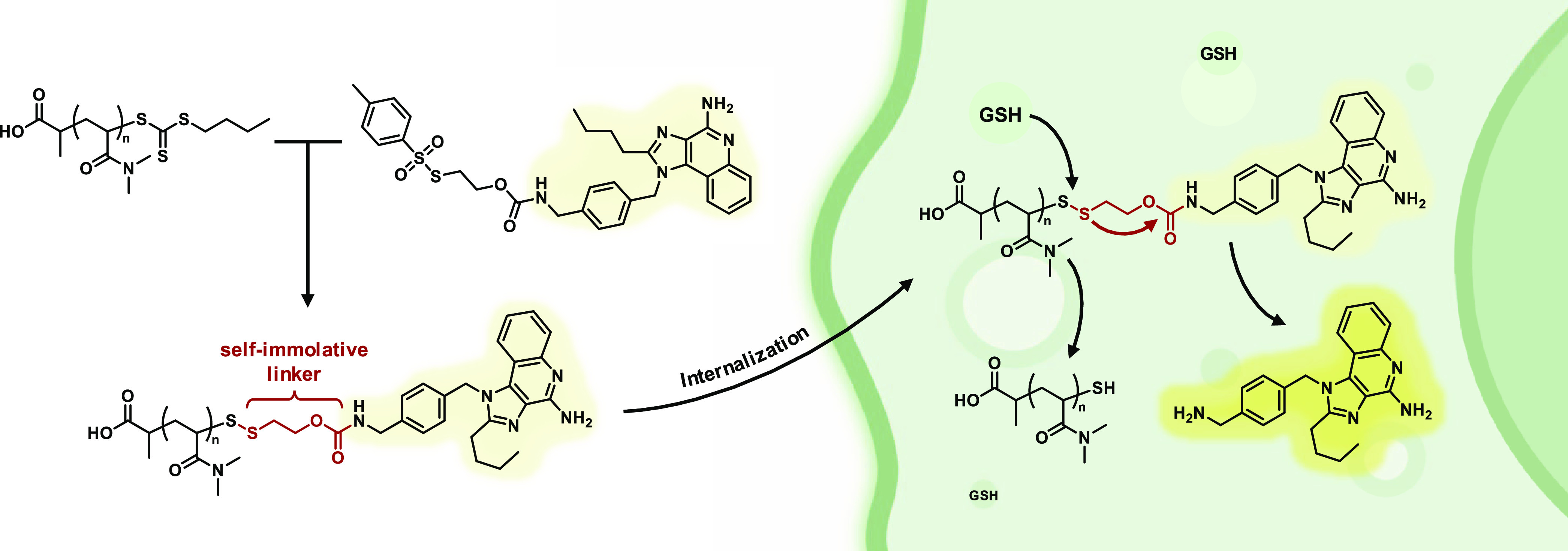
Polymer–drug conjugate derived from RAFT polymer ω-end functionalization with tosyl thiolate-bearing self-immolative linker. The disulfide linkage is reduced upon cellular uptake by GSH, and the cargo is released in its active form.

Herein, we demonstrate an approach to combine removal of RAFT end groups with the introduction of self-immolative linkers at the ω-end, thereby boosting the potential of these polymer–drug conjugates compared to irreversibly covalently bound alternatives. For that purpose, a tosyl thiolate-containing SIL was established that can be attached to RAFT-derived pDMA in a one-pot reaction. The cargo, a potent Toll-like receptor (TLR) 7 and TLR8 agonist 1-(4-(aminomethyl)benzyl)-2-butyl-1*H*-imidazo[4,5-*c*]quinolin-4-amine (IMDQ),^24^ can be bridged to the polymer scaffold via its free amine exploiting the SIL and released again without traces after internalization by intracellularly abundant GSH (see Figure 1). Furthermore, by using a cholesterol CTA, α-ω-bifunctional conjugates can be obtained, enhancing transport into the cell interior without compromising the efficacy of the system.

## Experimental Section

### Materials

Bromoethanol, *p*-nitrophenyl chloroformate, 3-toluenesulfonyl chloride, triethylamine, 2,4-quinolinediol, phenylphosphonyl dichloride, *p*-xylylendiamine, valeroyl chloride, calcium oxide, di-*tert*-butyl decarbonate, deuterated solvents, ethyl acetate, dimethyl sulfoxide (DMSO), sodium hydroxide (NaOH), hydrochloric acid (HCl), 2,4-dimethoxybenzyl amine, trifluoroacetic acid (TFA), platinum on carbon (10% Pt/C), benzylamine, benzyl alcohol, dibenzylamine, 4-dimethylaminopyridine (DMAP), 2,2′-azobis(2-methylpropionitrile) (AIBN), *n*-butylamine, *N*,*N*-dimethylacrylamide (DMA), sodium bicarbonate (NaHCO_3_), cholesterol, acryloxyethyl thiocarbamoyl rhodamine B, 2-mercaptoethanol, 2,2′-dipyridyl disulfide, and *N*,*N*′-dicyclohexylcarbodiimide (DCC) were obtained from Sigma-Aldrich. Potassium sulfite, sulfur, tris(2-carboxyethyl)phosphin (TCEP), and sodium sulfate were purchased from Acros Organics, Alfa Aesar, TCI Chemicals, and Carl Roth GmbH+ Co. KG, respectively. *N*,*N*-Dimethylformamide (DMF), chloroform, dichloromethane (DCM), tetrahydrofuran (THF), succinimidyl 4-(*N*-maleimidomethyl)cyclohexane-1-carboxylate (SMCC), and Gibco Dulbecco’s phosphate-buffered saline (DPBS) were obtained from Thermo Fisher Scientific Inc., and methanol (MeOH), acetone, and ethanol were obtained from Honeywell International.

### Characterization

#### Chromatography

Analysis grade solvents were used to elute compounds during chromatographic purifications on silica 60, 0.063–0.2 mm, from Macherey-Nagel GmbH & Co. KG and collected fractions were analyzed using thin-layer chromatography with silica gel 60 F_254_ from Supelco.

#### NMR Spectroscopy

^1^H, ^13^C, and 2D spectra were recorded on a Bruker Avance II 300 MHz or a Bruker Avance III 700 MHz spectrometer. Samples were dissolved in and referenced on deuterated solvents from Sigma-Aldrich and obtained spectra were analyzed with MestReNova 14.2.0 by Mestrelab Research.

#### Size-Exclusion Chromatography

Size-exclusion chromatography (SEC) was conducted on a SECcurity^2^Instrument purchased from PSS, Mainz, equipped with a SECcurity^2^Isocratic pump, a degasser, an auto sampler, an RI detector, a column thermostat, and a modified silica gel column (PFG columns, particle size: 7 μm, porosity: 100 Å + 1000 Å) purchased from PSS Polymer Standards Service GmbH. Measurements were conducted at 40 °C with hexafluoro-2-propanol (HFIP) bearing 3 g/L potassium trifluoroacetate as the eluent and a flow rate of 0.8 mL/min. Calibration was done with PMMA (PSS Polymer Standards Services GmbH), and elution diagrams were analyzed with PSS WinGPC from PSS Polymer Standard Service GmbH.

#### Mass Spectrometry

Matrix-assisted laser desorption ionization time-of-flight (MALDI-ToF) mass spectrometry were conducted using *trans*-2-[3-(4-*tert*-butylphenyl)-2-methyl-2-propenylidene]malononitrile (DCTB) as matrix on a rapifleX MALDI-ToF/ToF mass spectrometer from Bruker with a 10 kHz scanning smartbeam 3D laser (Nd:YAG at 355 nm) and a 10 bit 5 GHz digitizer in positive ion reflector mode. Calibration was done by polymer standards of poly(ethylene glycol). Mass spectrometry by electrospray ionization (ESI-MS) was performed on a SYNAPT G2-Si high-definition Q-TOF mass spectrometer (Waters Corp., Manchester, U.K.). The instrument was calibrated by clusters of sodium formate. All mass spectrometry data were processed with mMass version 5.5.0 and plotted with GraphPad PRISM version 5.02.

#### UV–Vis and Fluorescence Spectroscopy

A Thermo Scientific NanoDrop 2000c spectrophotometer equipped with an ultra-micro-cuvette 105.202-QS SD 10 mm from Hellma Analytics was used for UV–vis spectroscopy measurements. QUANTI-Blue secreted alkaline phosphatase and MTT assay readouts were performed on a Spark 20M multimode microplate reader from TecanTrading AG (Mannedorf, Switzerland).

#### Fluorescence Correlation Spectroscopy

FCS measurements were performed using a commercial LSM880 setup (Carl Zeiss, Jena, Germany) equipped with a C-Apochromat 40×/1.2 W water immersion objective. For excitation of the fluorescent dye tetramethyl rhodamine a helium–neon laser (543 nm) was used. The fluorescence light was collected with the same objective and after passing a pinhole directed to a spectral detection unit (Quasar, Carl Zeiss). There, the fluorescence light is separated spectrally by a grating element on a 32-channel array of GaAsP detectors operating in a single photon counting mode. The fluorescence signal in the range of 555–690 nm was detected. All measurements were performed in eight-well polystyrene-chambered cover glass (Laboratory-Tek, Nalge Nunc International). The obtained FCS autocorrelation curves were fitted with the theoretical model function for an ensemble of either one or two types of freely diffusing fluorescent species. The fits yielded the diffusion coefficients (*D*) of the studied species. Finally, the hydrodynamic radii (*R*_H_) were calculated using the Stokes–Einstein relation *R*_H_ = (κ_B_*T*)/6πη*D*, where κ_B_ is the Boltzmann constant, *T* is the temperature, and η is the viscosity of the solvent. For calibration, a diluted sample of Alexa 546 was used with a diffusion coefficient (*D*) of 341 μm^2^/s.^25^

### Synthesis

#### RAFT Chain-Transfer Agents

The synthesis of PABTC and Chol-PABTC can be found in the Supporting Information (Figures S2–S5 and S47–S50).

#### General Procedure of RAFT Polymerization

For a typical RAFT polymerization, 1.0 equiv of the RAFT chain-transfer agent PABTC or Chol-PABTC, 0.2 equiv of AIBN, and the monomer DMA were dissolved in DMF and subjected to at least three freeze–pump–thaw cycles. The amount of monomer was adjusted to the desired degree of polymerization. The polymerization was then conducted at elevated temperatures of 75 °C, and the product finally precipitated in cold diethyl ether affording the polymer as a yellow powder. Further details on the polymerization of pDMA at different molecular weights and cholesteryl-modified pDMA, as well as copolymers with acryloxyethyl thiocarbamoyl rhodamine B can be found in the Supporting Information (Figures S6, S51, S52, and S57).

#### End Group Reactive Activated Disulfide Reagents

The syntheses of pyridyl-based self-immolative linkers can be found in the Supporting Information (Figures S8–S17). Tosyl thiolate-based self-immolative linkers were synthesized as described in the Supporting Information (Figures S21–S36).

#### General Procedure of RAFT End Group Modification

For a typical RAFT end group modification, the respective pDMA polymer was treated with an excess of *n*-butylamine and the respective modified self-immolative linker in dry DMSO simultaneously. The *in situ*-generated thiol subsequently reacted most efficiently in a disulfide exchange reaction by replacing the tosyl unit of the self-immolative linker yielding the end group-modified polymer. For quantitative modifications, the polymers were typically treated with the reagents for 1 day and then precipitated into cold diethyl ether. Further details on the individual polymer end group modifications with the self-immolative linker derivatives can be found in the Supporting Information (Figures S37, S54–S56, S59, and S60).

### Biological Evaluation

#### Cell Culture

For fluorescence-activated cell scanning (FACS), microscopy and reporter assays, as well as cell viability experiments, RAW-Blue macrophages were cultured at 95% relative humidity, 5% CO_2_, and at 37 °C in Dulbecco’s modified Eagle’s medium (DMEM). Cell medium was additionally supplemented with 10% fetal bovine serum, 2 mM l-glutamine, 1 mM sodium pyruvate, 1% penicillin/streptomycin, and 0.01% zeocin.

#### Flow Cytometry

Into 24-well titer plates, 900 μL cell medium containing 200,000 RAW-Blue macrophages was seeded per well and incubated at 37 °C for 24 h. 100 μL of 0.75 mg/mL polymer solution in PBS was added as well as 100 μL of PBS as control and incubated for another 24 h at 37 °C. The next day, cell medium was aspirated, cells were washed with 1 mL of PBS, treated with 500 μL of 0.5 M EDTA in PBS and incubated for 15 min at 37 °C. Cells were detached by repeated pipetting, transferred to Eppendorf tubes, and centrifuged at 300*g* for 10 min at 5 °C. The supernatant was aspirated, and cells were resuspended in 150 μL of PBS and immediately passed on to perform FACS analyses on a BD Accuri C6 Plus flow cytometer with ∼30 000 counts each. All samples were performed as triplicates. During the whole process, after detachment, cells were kept on ice.

#### Confocal Microscopy

Into an ibidi μ-Slide 8 Well ibiTreat chambered coverslip, 180 μL of cell medium containing 50 000 RAW-Blue macrophages was seeded per well and incubated at 37 °C for 24 h. 20 μL of 0.25 mg/mL polymer solution in PBS was added as well as 20 μL of PBS as control and incubated for another 24 h at 37 °C. The next day, cell medium was aspirated, the cells were washed three times with 200 μL of PBS, incubated with 200 μL of 4% paraformaldehyde solution for 15 min at 37 °C, and washed three times with 200 μL of PBS again. Two drops of NucBlue Live ReadyProbes Reagent (Hoechst 33342) were dissolved in 1 mL of PBS, and 125 μL of this solution was applied per well and incubated at room temperature for 20 min. Cells were washed three times with 200 μL of PBS and stored under 2–3 drops of Fluoromount Aqueous Mounting Medium. The samples were imaged with a STELLARIS 8 FALCON microscope from Leica and processed with Leica Application Suite X (LAS X).

#### QUANTI-Blue Secreted Alkaline Phosphatase Assay

Per well in a 96-well plate, 90 000 RAW-Blue macrophages suspended in 180 μL of cell medium were seeded and incubated at 37 °C overnight. The next day, 20 μL of polymer–drug conjugate and control samples were applied to each well at various concentrations. After 48 h incubation at 37 °C, 150 μL of QUANTI-Blue solution was applied to each well of a new 96-well plate and 50 μL of supernatant of treated cells were added and incubated for 20 min at 37 °C. The QUANTI-Blue assay readout was performed by absorption measurements at 620 nm in quadruplicates.

#### MTT Assay

30 μL of 3-(4,5-dimethylthiazol-2-yl)-2,5-diphenyltetrazolium bromide (MTT, 2 mg/mL in PBS) was added to the remaining supernatant of cells after QUANTI-Blue assay at each well of the 96-well plate and incubated for 1 h at 37 °C. 100 μL of 10% SDS/0.01 M HCl were added to each well and incubated overnight at 37 °C. Cell viability was determined in quadruplicate (*n* = 4) in relation to positive PBS and negative 10% DMSO controls based on absorbance at 570 nm.

## Results and Discussion

As previously reported, RAFT polymerization end group modification with reductive-responsive self-immolative linkers can be obtained by a symmetric approach in which the SIL unit carries a central disulfide and is terminated by two cargo molecules on both ends.^26^ Reversible attachment and re-establishment of the original functionalities have already been demonstrated for these and other structures.^26,27^ However, a major drawback of this approach is the end group functionalization itself. The disulfide exchange reaction is primarily driven by one of the two cargo moieties acting as a leaving group due to the symmetrical character of this linker. Thus, one of the cargo molecules is sacrificed as essential requisite for the success of this modification reaction. This system-immanent loss can quickly become very cost-intensive and uneconomical, especially toward applications for biomedical and pharmaceutical purposes. Only a step-wise introduction of amine-reactive, reductive-responsive end groups via an SIL could so far be introduced for additional post-polymerization modification.^28^

In this study, we, therefore, introduce a more efficient and asymmetric approach for self-immolative RAFT polymer end group modification, in which the attachment of the SIL moiety is achieved by substitution of a suitable leaving group. A widely used structural element for activated disulfide units is dithiopyridine, where the free thiol of the RAFT end group analogously replaces the thiopyridyl unit in a disulfide exchange reaction. However, we observed that product mixtures occurred when combined with SIL chemistry (cf. Figure S20). We assume that, due to cyclization of the SIL unit, the afforded entropy gain competes with the leaving tendencies of the thiopyridyl group, resulting in both SIL-terminated and dithiopyridyl-terminated polymers. Moreover, the thiol-containing leaving group might additionally act as a nucleophile and react with the generated product, which also leads to such side product formation. A detailed characterization by ^1^H NMR spectroscopy and MALDI-ToF mass spectrometry for the modification of RAFT-polymerized pDMA (degree of polymerization DP = 25, *M*_n_ = 3297 g mol^–1^, *Đ* = 1.11, or DP = 50, *M*_n_ = 6970 g mol^–1^, *Đ* = 1.13, Figures S2–S5) with 2-(pyridin-2-yldisulfaneyl)ethyl benzylcarbamate (Figures S6–S17) and the proposed mechanism for the formation of the respective end group side products can be found in the Supporting Information (Figures S18–S20).

**Figure 2:**
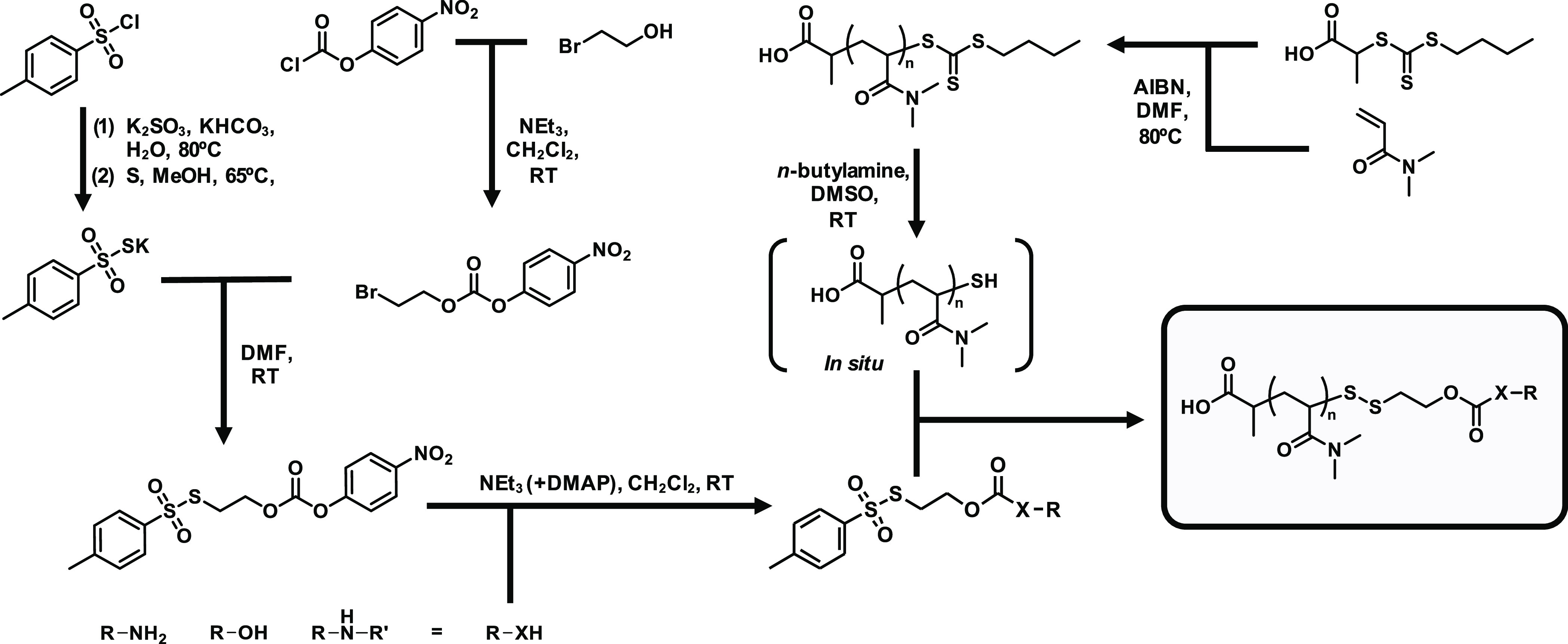
Synthetic scheme for the post-polymerization end group modification of RAFT-derived poly(*N*,*N*-dimethylacrylamide) (pDMA). Starting from tosyl chloride, potassium tosyl thiolate was generated in two steps and used for nucleophilic substitution of bromoethanol, which had before been transferred into a reactive carbonate moiety with 4-nitrochloroformate, to yield the functionizable self-immolative linker. Its reactive carbonate allows subsequent conjugation of component R bearing various functionalities (X = primary/secondary amines, primary alcohols). The resulting compounds can straightforwardly be coupled in a one-pot reaction after *in situ* liberation of terminal thiols by aminolysis of RAFT-polymerized pDMA.

Based on these findings, an alternative route via a tosyl thiolate-activated disulfide was chosen (see Figure 2). In contrast to the thiopyridyl linker, a non-nucleophilic toluoyl sulfinic acid is liberated during reaction with thiols. This does not interfere with the self-immolative end group modification according to the proposed reaction mechanism (Figure S20). For that purpose, a reactive carbonate was introduced with 4-nitrochloroformate starting from bromoethanol (Figure S23). The bromide was then exchanged by nucleophilic substitution with tosyl thiolate (Figures S24–S27), which can be prepared in two steps from tosyl chloride via 4-methylbenzene sulfinate (Figures S21 and S22). The resulting molecule contains both an activated disulfide with a non-nucleophilic leaving group that is reactive to free thiols and an activated carbonate where further structures can be introduced reversibly through various functional groups of choice. RAFT-polymerized pDMA (Figures S2–S6) can subsequently substitute in turn the tosyl group after aminolysis and *in situ* release of a terminal thiol, which leads to the desired SIL-modified RAFT polymer (Figure 2).

**Figure 3:**
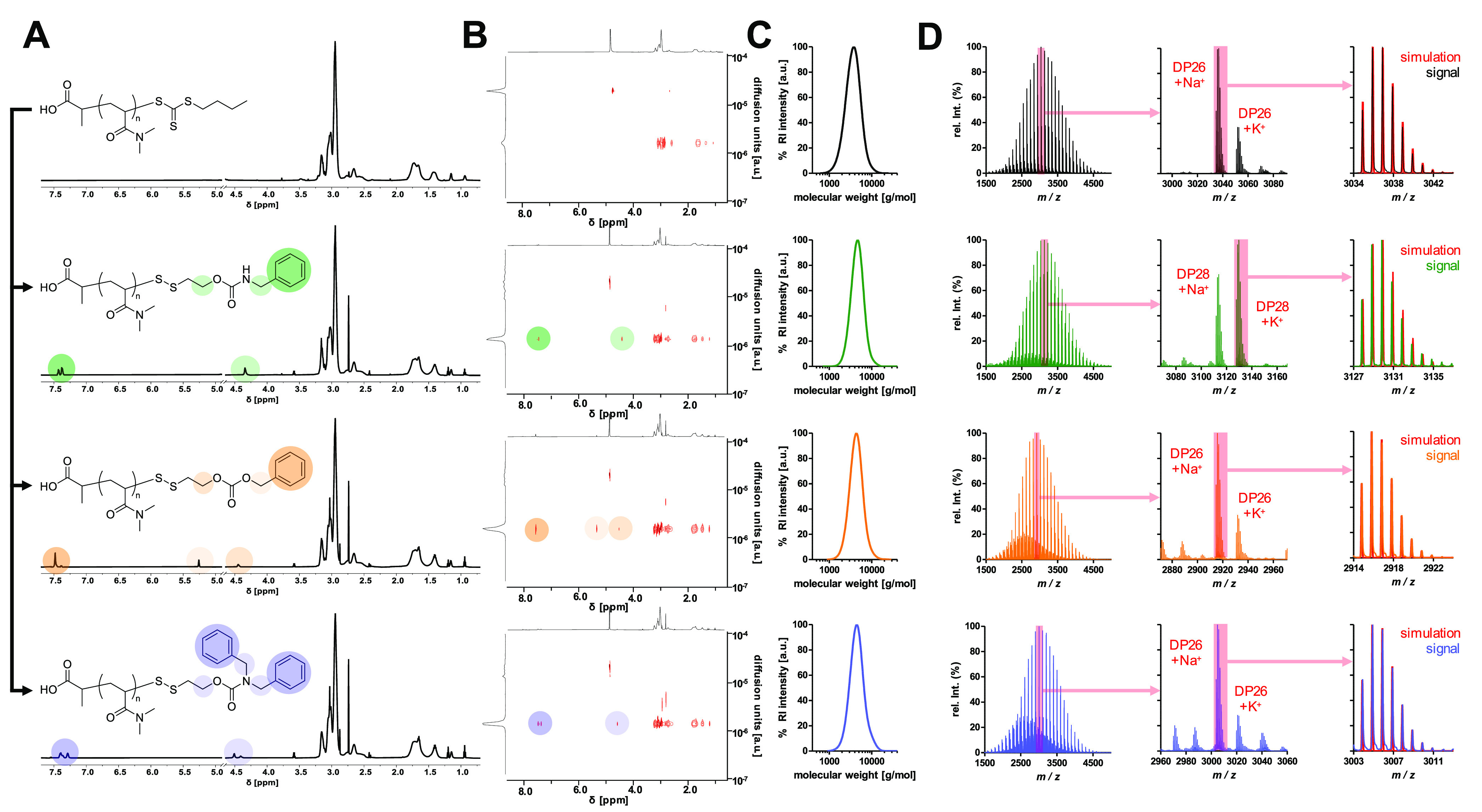
Characterization of end group-modified RAFT polymerization-derived pDMA (average DP = 25, black) with benzylamine (green), benzyl alcohol (orange), and dibenzylamine (blue) bridged by a self-immolative linker. (A) ^1^H NMR spectra of pDMA and its post-polymerization-modified derivatives. Respective aromatic and benzylic proton signals are highlighted. (B) ^1^H DOSY NMR experiments of pDMA and its end group-modified pDMA derivatives confirming the ligation of the three respective aromatic compounds to the polymer backbone. Similar diffusion properties of the respective aromatic proton signals to the aliphatic protons of the polymer backbone are recorded. (C) SEC traces of pDMA and end group-modified pDMAs. (D) MALDI-ToF MS data of pDMA and its end group-modified pDMA derivatives. Full polymer mass range (left), zoomed mass range of DP with highest relative intensity (middle), and overlay of DP with highest relative intensity and its corresponding simulated isotope pattern in red (right).

In order to demonstrate that this approach is versatilely applicable and that such disulfide systems are not only accessible for thiol-containing moieties, model compounds for primary alcohols, primary amines, and secondary amines were used in the following. For this purpose, benzyl alcohol (Figures S28–S31), benzylamine (Figure S32), and dibenzylamine (Figures S33–S36) were first attached to the low-molecular-weight SIL unit. Subsequently, in a one-pot reaction under mild conditions, the RAFT end group of pDMA (DP = 25) was converted by aminolysis into a free thiol, which could immediately react with the appropriately modified SIL unit in a disulfide exchange reaction (cf. Supporting Information). The obtained functionalized polymers were afterward carefully characterized by NMR, size-exclusion chromatography (SEC), and mass spectrometry (cf. Figures 3 and S37).

In the ^1^H NMR spectra, both the introduced aromatic protons and the protons adjacent to the carbonate and carbamate, respectively, could be observed (Figure 3A). Since these signals showed the same diffusion behavior as the signals of pDMA during ^1^H DOSY NMR spectroscopy experiments (Figure 3B), successful attachment of SIL units to the polymer backbone could be confirmed. Moreover, SEC traces did not show additional distributions but were still narrow and monomodal after the conversion (Figure 3C). Dispersities (*Đ*) below 1.2 and average molecular weights (*M*_n_) above 4000 g mol^–1^ were achieved for all functionalized polymers (pDMA-NHBz: *M*_n_ = 4287 g mol^–1^, *Đ* = 1.13; pDMA-NDiBz: *M*_n_ = 4138 g mol^–1^, *Đ* = 1.17; pDMA-OBz: *M*_n_ = 4005 g mol^–1^, *Đ* = 1.16). Most importantly, MALDI-ToF mass spectrometry measurements were finally recorded for detailed molecular end group analysis (Figure 3D). The signals obtained here were all in perfect agreement with the isotope simulation distributions of the expected end group-modified polymers. In addition, detailed ^1^H NMR end group analyses confirmed the introduction of the respective aromatic species via the self-immolative motif onto each polymer chain stoichiometrically (cf. Figure S37). Altogether, our collected data overall demonstrated the quantitative and straightforward modification of the RAFT polymer end groups with different functionalities by the asymmetric tosyl thiolate linker.

Having illustrated the general feasibility of this approach, we aimed at applying this approach for reversible polymer–drug conjugation with pDMA as a versatile alternative for drug PEGylation. In order to track the polymer chains in subsequent *in vitro* experiments, DMA was copolymerized with a small amount of a rhodamine B acrylate derivative (DP = 40, Figures S52 and S53). The percentage of fluorescently labeled polymer chains is accordingly negligible (as the main species is still unlabeled pDMA, the abbreviation pDMA will be continued in the following for simplification).

**Figure 4:**
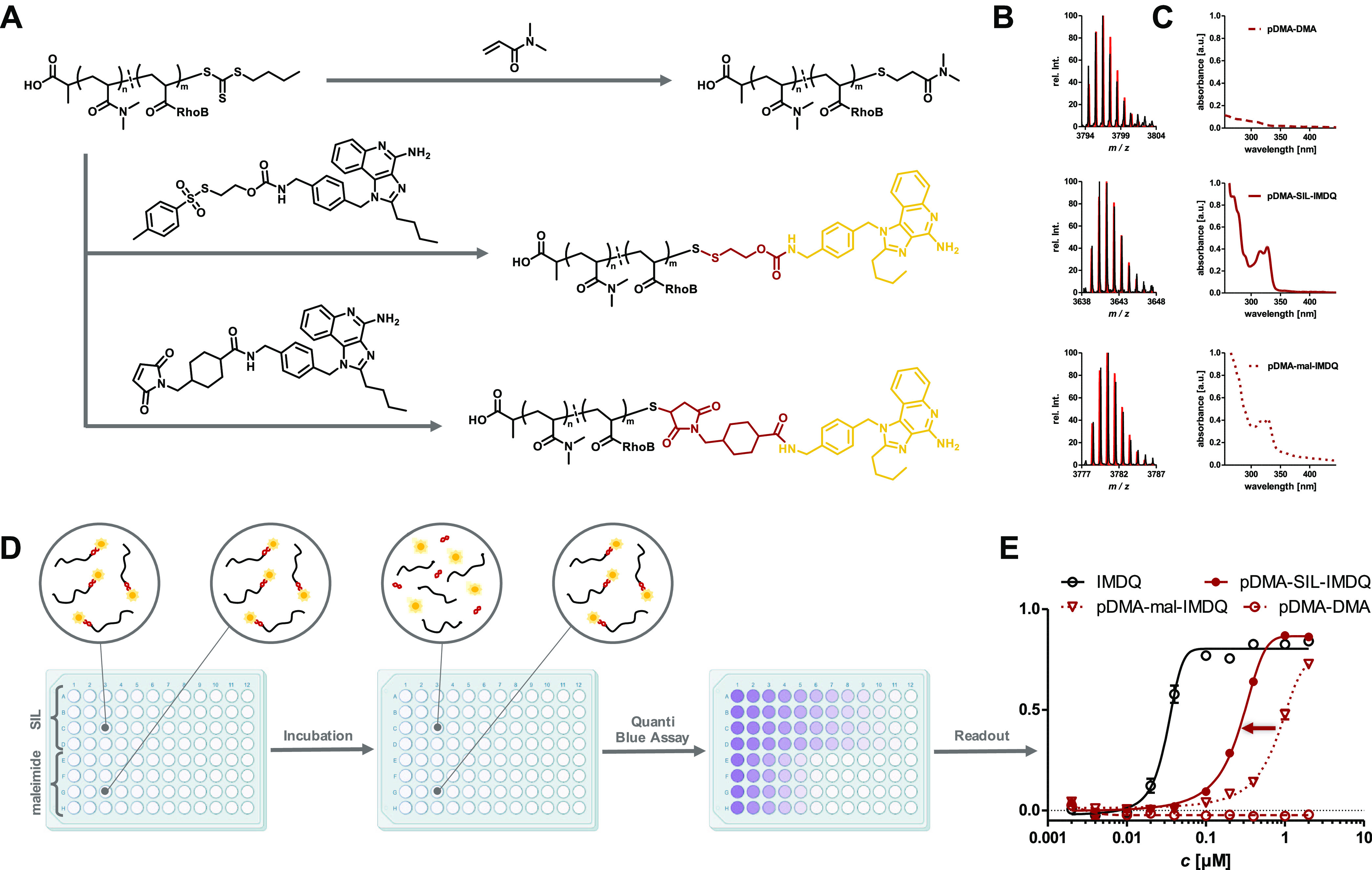
Characterization and application of RAFT polymer–immunodrug conjugates demonstrating the superiority of the *in situ*-introduced self-immolative linker strategy. (A) Post-polymerization modification of fluorescently labeled pDMA (average DP = 40) without active component (top), IMDQ bridged via the self-immolative motif (middle) and IMDQ attached via thioether (bottom). (B) MALDI-ToF MS data of respective DP with highest relative intensity and its corresponding simulated isotope pattern in red. (C) UV–vis spectra of post-polymerization-modified polymers exhibiting the characteristic IMDQ absorbance at 322 nm. (D) Illustration of the *in vitro* reporter cell assay. Cells were incubated with polymer samples on a 96-well plate for 48 h, when the polymer samples can get intracellularly processed and QUANTI-Blue assay can be performed to verify enhanced receptor activity for the SIL conjugate. Color intensity, reflecting induced phosphatase activity, rises from increasing IMDQ activity. (E) Results of the assay readout. The data of the self-immolative linked IMDQ (red) were shifted toward free IMDQ (black) in comparison to thioether-linked IMDQ (red, dotted). Polymer without active compound exhibited no activity at all (red, dashed) (illustration of 96-well plates was adapted from BioRender.com).

To illustrate the superiority of this system over conventional conjugation strategies, three different end group functionalizations were conducted (Figure 4A): The TLR agonist IMDQ was again functionalized with the tosyl thiolate-containing SIL (Figures S38–S42) to generate a reversible ligation to pDMA (pDMA-SIL-IMDQ, *M*_n_ = 7360 g mol^–1^, *Đ* = 1.18), as demonstrated before (Figure S54). Alternatively, IMDQ was converted into a maleimide-containing variant (Figures S43–S45) that ultimately generates a noncleavable thioether bridge at the polymer end (pDMA-mal-IMDQ, *M*_n_ = 7583 g mol^–1^, *Đ* = 1.21, Figure S55). Additionally, by treatment with excess DMA after aminolysis, the liberated thiol can also undergo a Michael addition to the acrylamide affording a DMA thioether pDMA (pDMA-DMA, *M*_n_ = 6465 g mol^–1^, *Đ* = 1.17, Figure S56). The latter should not have any therapeutic activity and served as a negative control. For all cases, successful end group modification could again be confirmed by mass spectrometry with isotope patterns fitting with simulated signals (Figure 4B).

Subsequently, the TLR agonistic activity of these polymers was examined *in vitro* on RAW-Blue reporter cells. Through TLR-mediated activation of the nuclear factor ’kappa-light-chain-enhancer’ of activated B-cells (NF-κB) signaling cascade, this macrophage cell line was genetically modified to secrete embryonic alkaline phosphatase (SEAP), which in turn can be quantified from cell culture medium spectrophotometrically. Prior to applying the samples onto the cells, UV–vis spectroscopy measurements of the polymer samples were recorded to assure that pDMA-SIL-IMDQ and pDMA-mal-IMDQ contained the same amount of IMDQ, as nonconjugated IMDQ bearing a characteristic absorption maximum at 322 nm (Figure 4C).

When cells were treated with the conjugates, both polymer conjugates carry the immunomodulator covalently bound to the polymer backbone for both pDMA-SIL-IMDQ and pDMA-mal-IMDQ. In contrast, after 48 h of incubation at 37 °C, the conjugates should get taken up by macrophages and sufficiently processed, allowing intracellular reductive degradation of the SIL moiety affording a traceless release of IMDQ and, therefore, an increased activity of pDMA-SIL-IMDQ over pDMA-mal-IMDQ (cf. Figure 4D). Indeed, the readout of the cell culture medium 48 h after RAW-Blue cell incubation with the samples confirmed this trend. While pDMA-DMA did not show any TLR agonistic activity at all, pDMA-mal-IMDQ was able to stimulate the expression of SEAP to some extent only at reasonably higher concentrations. Interestingly, a clear shift toward activation rates of native IMDQ and thus an increase in activity could be observed for pDMA-SIL-IMDQ compared to pDMA-mal-IMDQ (cf. Figure 4E). Therefore, already at much lower concentrations, similar TLR activation can be achieved for the reducible SIL-IMDQ polymer in comparison with the nondegradable version. Still, the full potency of the native drug had not yet been fully recovered, probably due to stealth properties of pDMA compared to the nonconjugated drug. However, polymer–drug conjugation is assumed to be superior during actual *in vivo* setups by improving the pharmacokinetic profile through prolonged blood circulation instead of rapid renal excretion. Moreover, like many other potent immunomodulators, IMDQ tends to exhibit severe systemic distribution, which can be overcome by conjugation to a macromolecular carrier.^29−34^

Often, additional mechanisms are exploited not only to prolong the circulation of the drug in the bloodstream after systemic application but also to enforce more specific targeting. For example, cholesterol has now frequently been applied for translocation to lymphoid tissue by noncovalent binding to albumin as a hitch hiker and, bound to a polymer–drug conjugate, cholesterol can thereby transport the immunomodulators to their desired site of action. Additionally, its membrane permeability fosters interaction and cellular uptake especially by antigen presenting cells as major targets for immunodrug delivery.^35−37^

**Figure 5:**
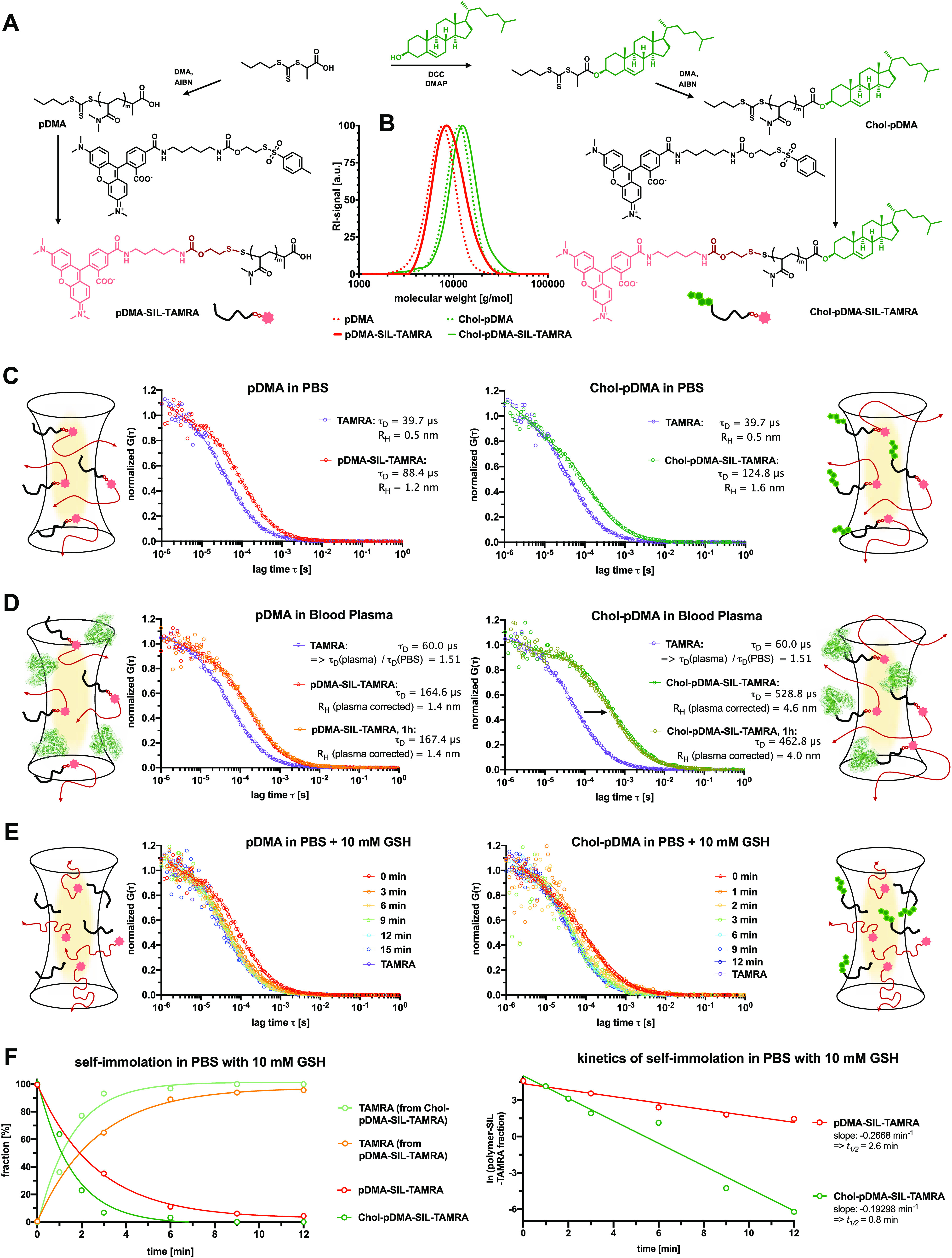
Comparison of the RAFT polymer-derived SIL-TAMRA conjugates with and without α-end cholesterol modification. (A) Synthetic scheme of the chain-transfer agent modification with or without cholesterol prior to RAFT polymerization of DMA (targeting an average DP = 50). The resulting polymers with the cholesterol α-end group exhibit improved membrane permeability and capability of noncovalently binding to albumin, while the trithiocarbonate end group can still be exploited for SIL-TAMRA conjugation. (B) Successful TAMRA conjugation does not alter the polymer distribution verified by SEC. (C) Fluorescence correlation spectroscopy (FCS) measurements of pDMA-SIL-TAMRA (left) and Chol-pDMA-SIL-TARMA in PBS and their corresponding results. (D) FCS measurements of pDMA-SIL-TAMRA (left) and Chol-pDMA-SIL-TARMA in full human blood plasma. Both conjugates remain stable and do not release the dye. While pDMA-SIL-TAMRA remains as individual polymer chains in full blood plasma (left), Chol-pDMA-SIL-TARMA exhibits lager sizes indicating interactions with plasma protein components including albumin. (E) FCS measurements of pDMA-SIL-TAMRA (left) and Chol-pDMA-SIL-TARMA in PBS with 10 mM glutathione (GSH) indicating the rapid reductive-responsive release of the dye. (F) A fit of two fluorescent species was applied to these measurements for quantifying the fraction of polymer–TAMRA conjugates and released TAMRA (left). These data can further be analyzed by first-order release kinetics (right) revealing a half-life of about *t*_1/2_ = 2.6 min for pDMA-SIL-TAMRA and *t*_1/2_ = 0.8 min for Chol-pDMA-SIL-TAMRA.

To investigate whether this approach could also be employed for the demonstrated system, a cholesterol-modified CTA was synthesized (Figures S47–S50) and subjected to homopolymerization with DMA alone affording Chol-pDMA (Figures 5A and S51, *M*_n_ = 10 572 g mol^–1^, *Đ* = 1.13). pDMA (*M*_n_ = 6970 g mol^–1^, *Đ* = 1.13) prepared from nonfunctionalized CTA served as a reference compound (note that both polymers were now only prepared from DMA at DP = 50, no copolymerization with traces of rhodamine B acrylate was applied). Consequently, in order to study the stability of the attached functionality via the reductive SIL onto both polymers’ end, a fluorescent tracer molecule could reversibly be ligated. For that purpose, tetramethyl rhodamine cadaverine (TAMRA) was extended by the 2-(tosylthio ethoxy)-carbonyl unit (Figure S46) and then conjugated in a one-step procedure by the SIL motif to both polymers (Figure 5A). A gradual shift of the molecular weight distribution was obtained for both pDMA-SIL-TAMRA (*M*_n_ = 8478 g mol^–1^, *Đ* = 1.15) and Chol-pDMA-SIL-TAMRA (*M*_n_ = 11474 g mol^–1^, *Đ* = 1.18) by size-exclusion chromatography compared to the initial pDMA and Chol-pDMA (Figure 5B).

The reversibly attached fluorophore on both polymers can be monitored by fluorescence correlation spectroscopy (FCS) in order to evaluate the stability of the formed self-immolative disulfide bond under biological relevant conditions as well as its release kinetics upon reductive stimuli using Figure 5C–E. FCS records the diffusion properties of the fluorescent species (intact polymer–fluorophore conjugate or released fluorophore) even in complex biological media in the presence of other macromolecules (e.g., plasma proteins).^38,39^ In PBS, both conjugates remained stable and provided hydrodynamic radii of *R*_H_ = 1.2 nm for pDMA-SIL-TAMRA, and slightly larger for the cholesterol-functionalized polymer Chol-pDMA-SIL-TAMRA at *R*_H_ = 1.6 nm (Figure 5C). These sizes are in agreement with other previously determined hydrodynamic radii of water-soluble polymers providing similar molecular weights,^40^ while the unconjugated free dye TAMRA yielded a hydrodynamic radius *R*_H_ of 0.5 nm.

Next, these measurements were repeated in human blood plasma (Figure 5D); however, the diffusion times of TAMRA thereby increased by a factor of 1.5. This is related to the higher viscosity of the blood plasma compared to PBS, which has previously been investigated during similar FCS plasma measurements.^41^ Taking this value into account, the hydrodynamic radius of pDMA-SIL-TAMRA was determined at *R*_H_ = 1.4 nm, which is only slightly larger than in PBS (Figure 5D), thus confirming that pDMA provides stealth-like properties and is not interacting with the plasma proteins. Interestingly, even after 1 h of incubation in human blood plasma, the autocorrelation curve remained stable. Thus, no decrease in size could be found, confirming the stability of the self-immolative disulfide bond under full plasma conditions.

Interestingly, after incubation in human blood plasma, the cholesterol- and dye-modified polymer Chol-pDMA-SIL-TAMRA massively increased in size. While it provided a hydrodynamic radius of *R*_H_ = 1.6 nm in PBS, this increased to a value of *R*_H_ = 4.6 nm in human blood plasma and remained in this size regime even after 1 h of incubation (Figure 5D). These observations clearly underline the expected interaction of the adjacent cholesterol group with various plasma protein components including albumin and using this mechanism for hitch hiking, e.g., for lymph node accumulation.^35−37^

Next, both polymers pDMA-SIL-TAMRA and Chol-pDMA-SIL-TAMRA were incubated with 10 mM glutathione (GSH), mimicking the reductive concentrations of intracellular environments (Figure 5E). Immediately upon exposure to GSH, a rapid shift of both autocorrelation curves was found, and within 12 min for pDMA-SIL-TAMRA and within 6 min for Chol-pDMA-SIL-TAMRA, all TAMRA was fully released from the polymers (the autocorrelation curves were similar as for the free dye TAMRA—Figure 5E). The recorded data were then evaluated by applying a fit of two fluorescent species to quantify the fraction of intact polymer-bound TAMRA and released TAMRA (Figure 5F). By applying first-order release kinetics for both polymers, the half-lives of the self-immolation process could be calculated. For pDMA-SIL-TAMRA, a half-life of about *t*_1/2_ = 2.6 min, while for Chol-pDMA-SIL-TAMRA, this process was 3 times faster with *t*_1/2_ = 0.8 min (Figure 5F), probably related to a preferential interaction of the adjacent cholesterol group with the reducing agent glutathione.

Altogether, these observations confirmed that the self-immolative linkers respond rapidly upon external reductive stimuli, while they remain stable under full plasma conditions and allow hitch hiking by plasma components mediated by the introduction of an additional cholesterol group onto the heterotelechelic pDMA end groups.

**Figure 6:**
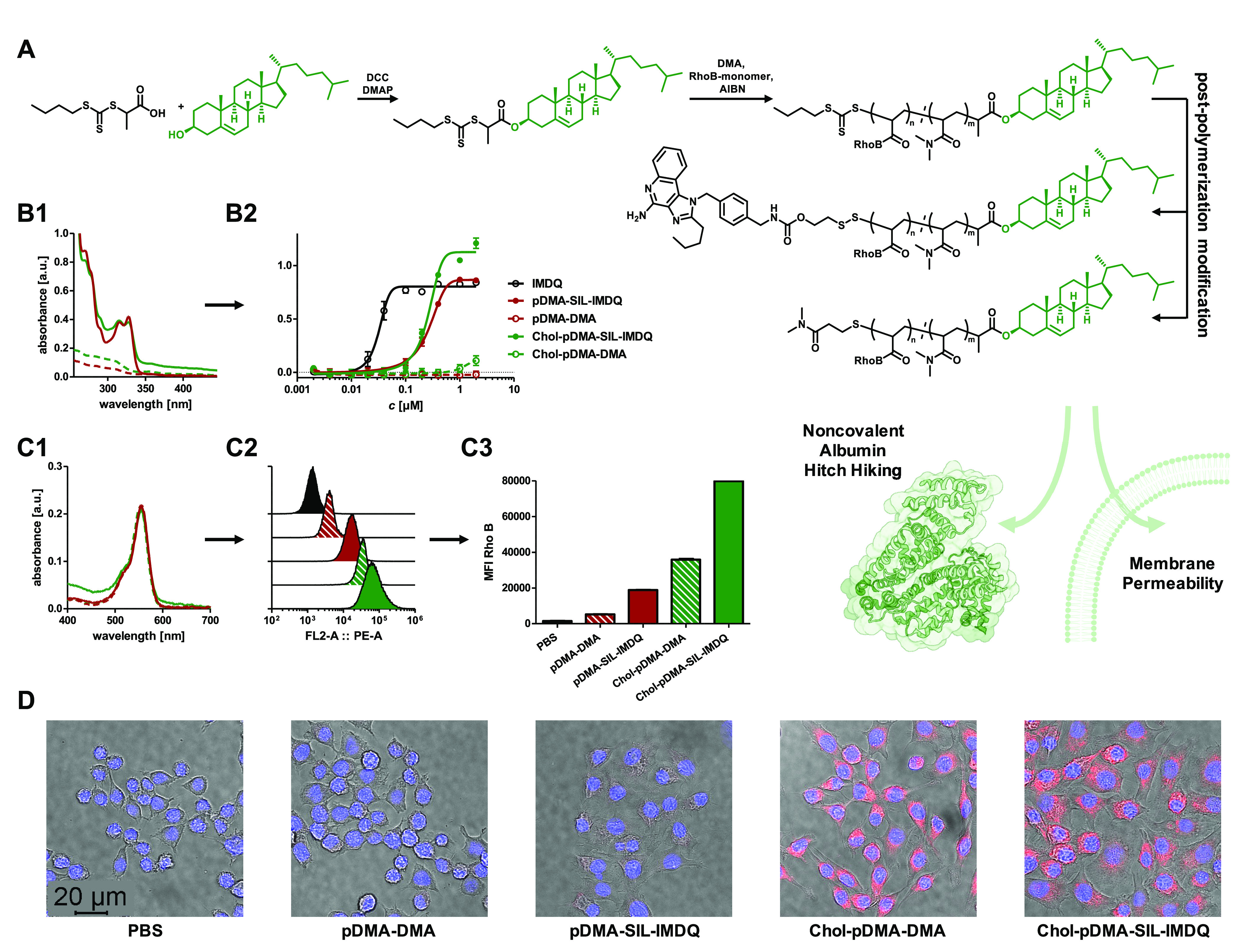
Comparison of RAFT polymer-derived SIL–immunodrug conjugates with and without α-end cholesterol modification. (A) Synthetic scheme of chain-transfer agent modification with cholesterol prior to polymerization. Resulting polymer-bearing cholesterol moiety exhibiting improved membrane permeability and capability of noncovalently binding to albumin. The trithiocarbonate end group can still be exploited for SIL–immunodrug conjugation (targeted average DP = 40). (B) Successful immunodrug conjugation verified by characteristic IMDQ absorbance at 322 nm via UV–vis spectroscopy confirming similar loading for polymer–drug conjugates (B1) prior to their application on RAW-Blue macrophages and subsequent QUANTI-Blue reporter cell assay readout. The recorded absorbances reveal a similar level of IMDQ activity at given concentrations for the polymers with and without cholesterol modification. Interestingly, the cholesterol IMDQ conjugate provided a more intense activity; however, cholesterol functionalization itself also exhibited a slight intrinsic immune activation (B2). The cholesterol polymer conjugate without IMDQ also slightly triggers immune activation at these elevated concentrations. (C) Polymer samples with similar fluorescent labeling determined by UV–vis spectroscopy (C1) were applied on RAW-Blue macrophages leading to the respective histograms by flow cytometry (C2). Uptake was generally found at higher levels for the cholesterol-functionalized polymers, which could further be quantified by mean fluorescence intensity analyses (C3). (D) Merged confocal microscopy images confirming enhanced cellular uptake and internalization for the cholesterol-modified fluorescently labeled polymers (1BM0 was used for albumin structure and processed with BioRender.com).

To further elaborate on the delivery performance of the Chol-pDMA polymers for the reductive-responsive transport of the TLR 7/8 agonist IMDQ as SIL-payload, additional Chol-pDMA polymers were synthesized with the rhodamine B acrylate species copolymerized into the polymer backbone (DP = 40, *M*_n_ = 7126 g mol^–1^, *Đ* = 1.12, Figures S57 and S58). The SIL-IMDQ moiety was subsequently introduced to the RAFT polymer end group following the beforementioned protocol (Chol-pDMA-SIL-IMDQ, *M*_n_ = 8946 g mol^–1^, *Đ* = 1.19) (Figures 6A and S59). This conjugate, together with a negative control (Chol-pDMA-DMA, *M*_n_ = 7169 g mol^–1^, *Đ* = 1.13) (Figures 6A and S60), was compared to the corresponding polymer conjugates without cholesterol for their immune stimulatory activity.

To ensure approximately comparable IMDQ loading, UV–vis spectroscopy measurements were recorded, demonstrating similar IMDQ absorbances (Figure 6B1). During subsequent RAW-Blue stimulation studies, Chol-pDMA-SIL-IMDQ did not lose any activity compared to pDMA-SIL-IMDQ, despite its additional lipid modification. In fact, even a stronger activation of the reporter genes was revealed by the introduction of the cholesterol moiety at higher concentrations, which could also be observed when comparing the two negative controls (Figure 6B2). This could indicate an additional, independent signaling pathway caused by the cholesterol modification itself since the combination of cholesterol and pDMA leads to the formation of amphiphilic structures that might also be recognized by other TLRs such as TLR4, which bears lipopolysaccharide (LPS) recognition structures. During these experiments, no difference in influence on the cells’ viability was found between Chol-pDMA and pDMA (Figure S61).

The rhodamine B backbone labeling of the IMDQ conjugates could finally be exploited to visualize the polymer internalization into RAW-Blue macrophages. The polymers’ degree of labeling was confirmed by UV–vis spectroscopy (Figure 6C1) before RAW-Blue macrophages were incubated with similar sample concentrations and then analyzed by flow cytometry (Figure S1). The resulting histograms exhibited a significantly higher shift and thus an increased cellular uptake for the cholesterol conjugates than for the polymers without cholesterol (Figure 6C2). Also, the mean fluorescence intensity (MFI) values demonstrated higher values for the cholesterol-bearing polymers. Interestingly, an increased uptake of the polymers was already present by the reversible SIL conjugation of IMDQ, which may be accounted for by its additional hydrophobic character compared to the DMA thioether (Figure 6C3). Consequently, the highest mean fluorescence intensity (MFI) values were achieved for those cells treated with Chol-pDMA-SIL-IMDQ. Uptake and cell internalization were finally confirmed by confocal microscopy, providing strongest intracellular fluorescence for the cholesterol-modified pDMA conjugates (Figures 6D and S62).

## Conclusions

Overall, an efficient and quantitative conversion of thiol-containing terminal groups of heterotelechelic RAFT-derived polymers into a self-immolative motif in a one-pot reaction was demonstrated in this study. By disulfide activation using a tosyl thiolate group, various functionalities could be generated at the polymer end of pDMA after *in situ* exposure of a terminal thiol by aminolysis during disulfide exchange reactions. This was exemplified first for benzyl alcohol, benzylamine, and dibenzylamine, as model compounds for alcohols and primary and secondary amines, respectively, followed by the insertion of the fluorescent dye TAMRA as a tracer molecule or the potent small molecular immunomodulator IMDQ as a TLR 7/8 agonist. The dye conjugate could be used for verifying stabilities in full human blood plasma as well as immediate self-immolation release kinetics in the presence of 10 mM GSH, while the IMDQ conjugate was applied to a reporter cell line, demonstrating the boosted activity of the immunomodulator by the self-immolative processes compared with a nondegradable variant of the polymer–drug conjugate. Furthermore, additional incorporation of a cholesterol moiety at the α-end of the polymer facilitated the binding to plasma proteins and uptake into cells *in vitro*, as evidenced by fluorescence correlation spectroscopy, flow cytometry, and confocal imaging. This additional modification did not affect the activity of IMDQ but actually resulted in an overall stronger signal output in the reporter assay compared to conjugates without cholesterol at the same concentration and may therefore be applicable for albumin-mediated hitch-hiking delivery mechanisms to lymph nodes.

In conclusion, this methodology established a straightforward procedure that combines elegant reductive-responsive self-immolation chemistry with the broad field of available RAFT polymers. With pDMA as RAFT-polymerizable water-soluble polymer, it opens up a diverse range of applications for these reversible polymer–drug conjugates beyond conventional PEGylated alternatives.

## Supporting Information Available

Further details on the conducted syntheses and characterization including experimental data for all RAFT polymerizations; the preparation of chain-transfer agents and the post-polymerization building blocks; and additional data on polymer characterization, NMR spectra and assignments, SEC data, MALDI spectra, etc. (PDF)
