# Supplementary material for: Efficient Self-Immolative RAFT End Group Modification for Macromolecular Immunodrug Delivery
Source: Biomacromolecules. 2023 Apr 24;24(5):2380–91. doi: 10.1021/acs.biomac.3c00239 (PMC10170519; doi:10.1021/acs.biomac.3c00239)
Supplement: Supplementary file 1 — bm3c00239_si_001.pdf [file bm3c00239_si_001.pdf]

Efficient Self-Immolative RAFT End Group Modification for Macromolecular Immunodrug Delivery

– Supporting Information –

Maximilian Scherger,[a] Yannick A. Pilger,[b] Judith Stickdorn,[a] Patric Komforth,[a] Sascha Schmitt,[a] Kaloian Koynov,[a] Hans Joachim Räder[a] and Lutz Nuhn*[a,b]

[a]: Max Planck Institute for Polymer Research, Ackermannweg 10, 55128 Mainz, Germany
E-mail: lutz.nuhn@mpip-mainz.mpg.de

[b|: Chair of Macromolecular Chemistry, Julius-Maximilians-University Würzburg, Röntgenring 11, 97070 Würzburg, Germany
E-mail: lutz.nuhn@uni-wuerzburg.de

*: corresponding author: Prof. Dr. Lutz Nuhn

(lutz.nuhn@mpip-mainz.mpg.de or lutz.nuhn@uni-wuerzburg.de)

Flow Cytometry Gating Strategy

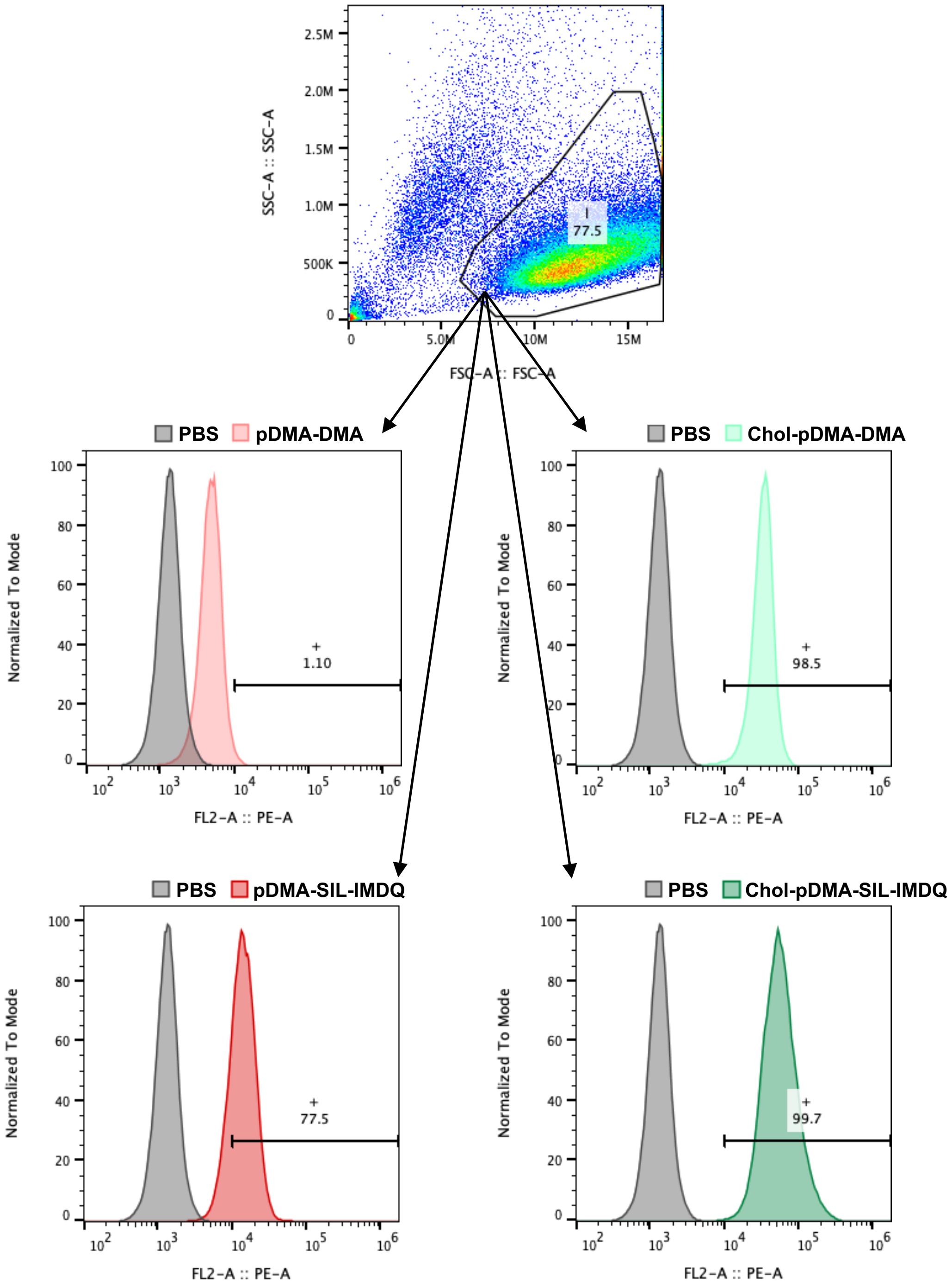

Figure S1: Gating strategy for RAW-Blue macrophages incubated with PBS or pDMA-DMA/ pDMA-SIL-IMDA without cholesterol end group as well as Chol-pDMA-DMA/ Chol-pDMA-SIL-IMDQ with cholesterol end group at 75 µg/mL for 24 h at 37 °C.

Syntheses and Characterization:

2-(butylthiocarbonothioylthio)propanoic acid (PABTC)

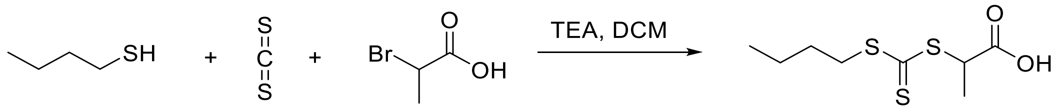

The synthesis was carried out as previously reported.[1] Triethylamine (4.24 mL, 30.4 mmol) was added dropwise under nitrogen atmosphere to a solution of 1-butanethiol (3.00 mL, 27.8 mmol) in 30 mL dry DCM. This reaction mixture was cooled to 0 °C and a solution of carbon disulfide (1.83 mL, 30.4 mmol) in 30 mL dry DCM was added dropwise under stirring. The cooling was removed and the solution was stirred for another 30 minutes before 2-bromopropionic acid (2.74 mL, 30.4 mmol) in 15 mL dry DCM was added dropwise. After 3 h of stirring, the solvent was removed under reduced pressure and the residue was diluted with cyclohexane. This solution was extracted with 10% HCl, water and brine and the organic layer was dried over sodium sulfate and concentrated in vacuum. Recrystallization from hexane gave 2-(butylthiocarbonothioylthio)propanoic acid as yellow crystals (4.3 g, 65%).

^{1}H NMR (400 MHz, $CDCl_3$, *δ*): 4.87 (q, 1H, H_3C–C*H*–), 3.37 (t, 2H, –C*H*$_2$–S–), 1.70 (m, 2H, –C*H*$_2$–CH_2–S–), 1.63 (d, 3H, –C*H*$_3$), 1.43 (m, 2H, H_3C–C*H*$_2$–), 0.94 (t, 3H, –CH_2–C*H*$_3$).
^{13}C NMR (100 MHz, $CDCl_3$): *δ* [ppm] = 221.95, 117.36, 47.59, 37.26, 30.00, 22.19, 16.73, 13.73.

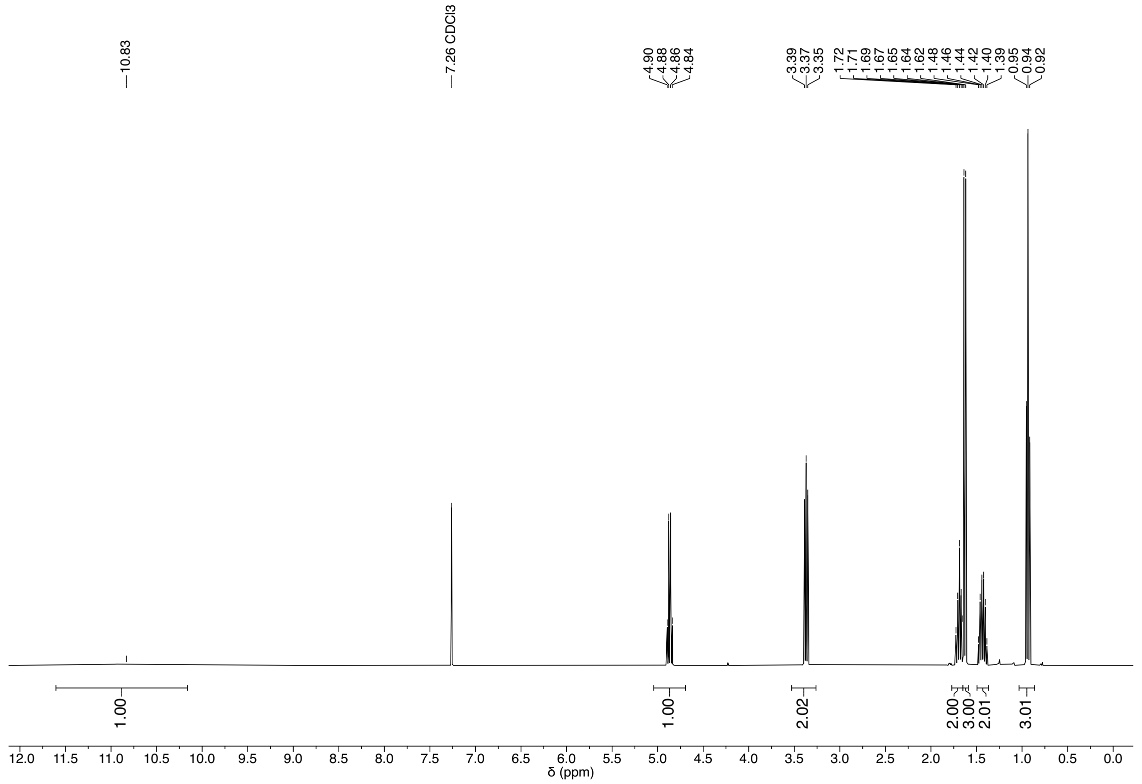

Figure S1: ^{1}H NMR (400 MHz, $CDCl_3$) of 2-(butylthiocarbonothioylthio)propanoic acid.

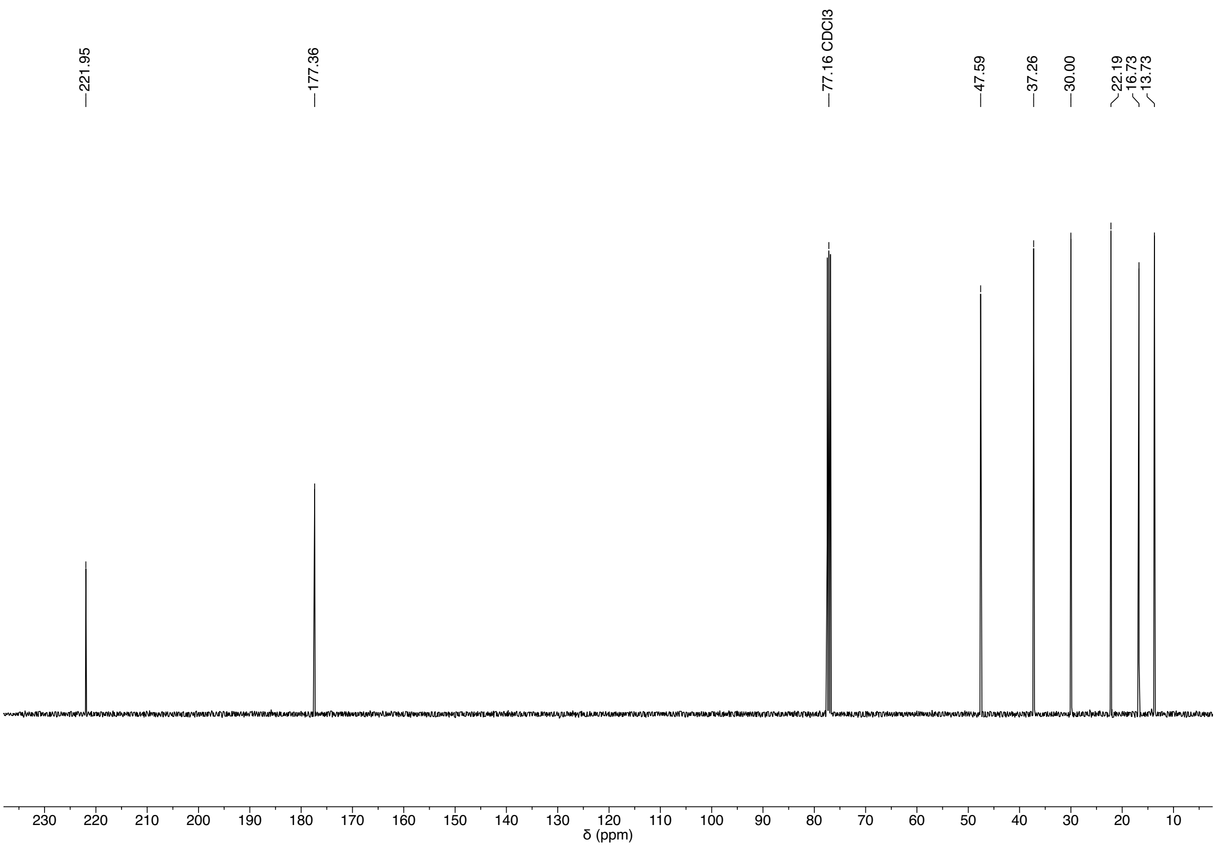

Figure S3: ^{13}C NMR (100 MHz, $CDCl_3$) of 2-(butylthiocarbonothioylthio)propanoic acid.

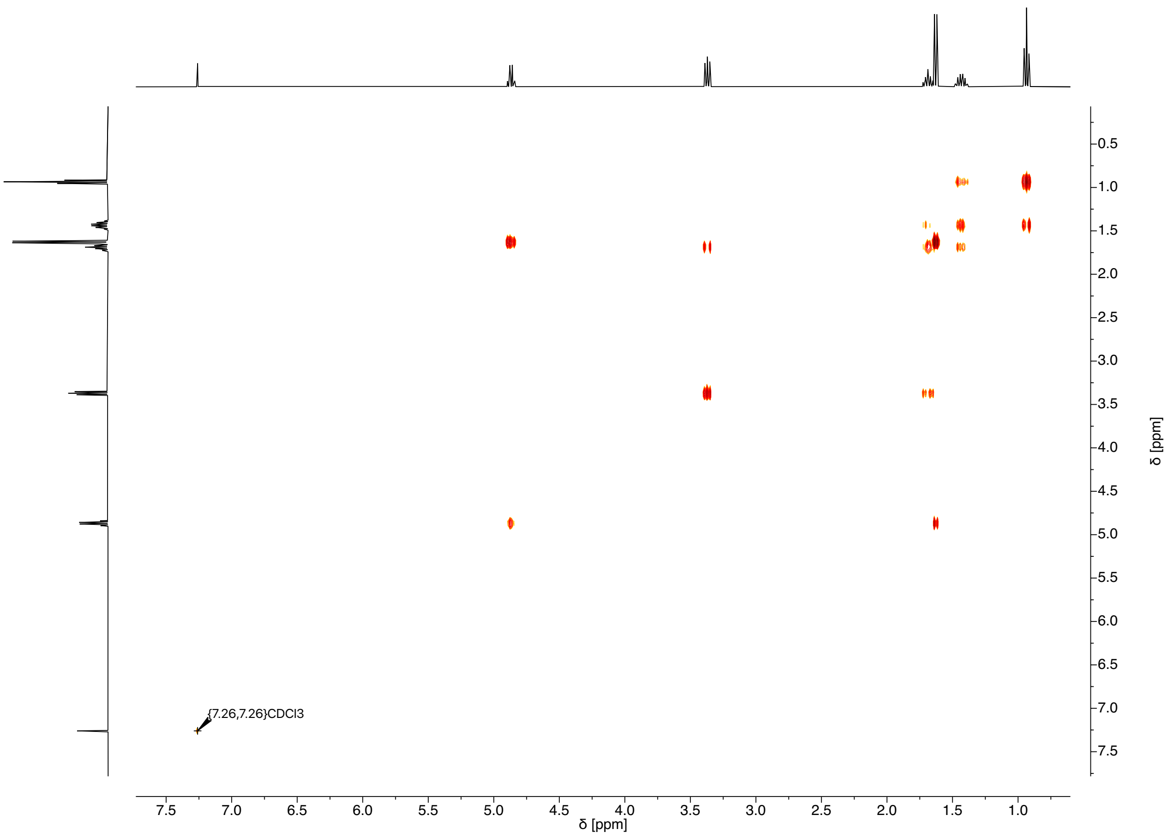

Figure S4: ^{1}H,^{1}H COSY spectrum of 2-(butylthiocarbonothioylthio)propanoic acid.

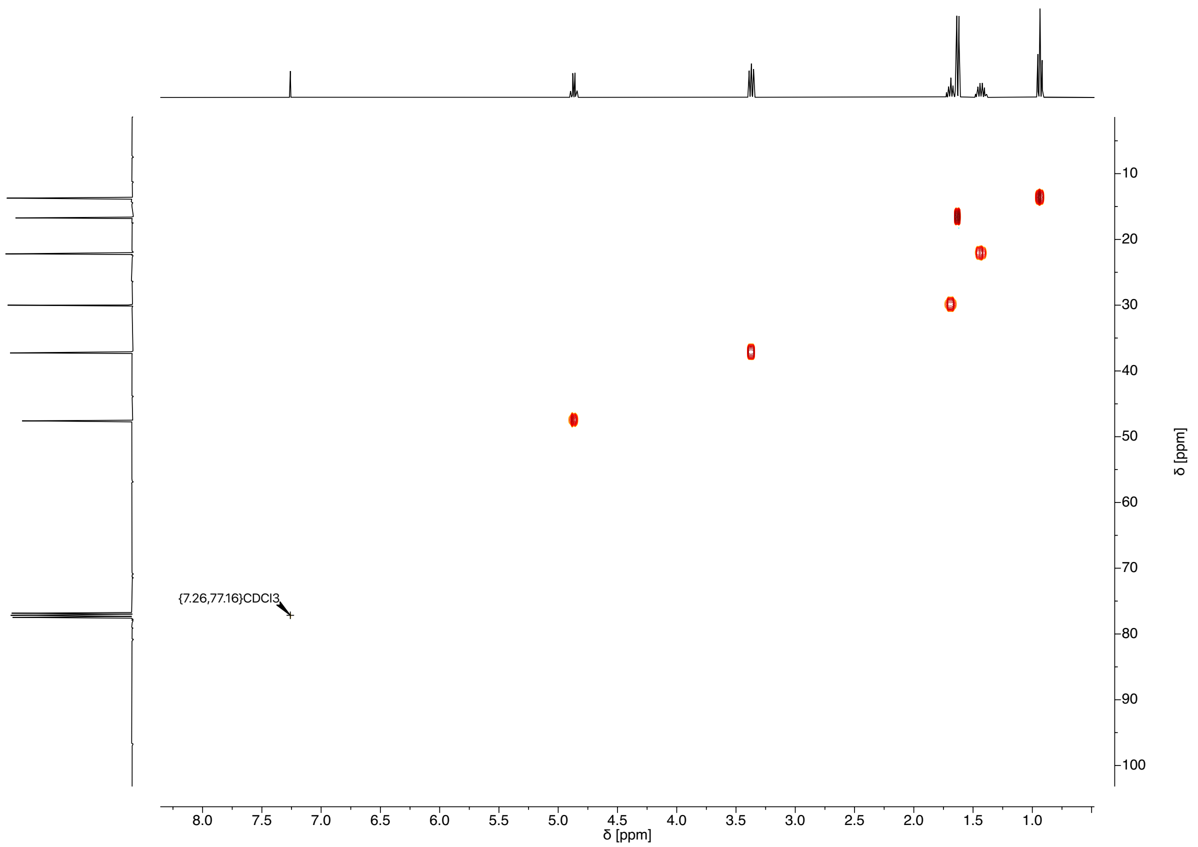

Figure S5: $^{1}H,^{13}C$ HSQC spectrum of 2-(butylthiocarbonothioylthio)propanoic acid.

Polymerization of *N,N*-dimethylacrylamide with PABTC

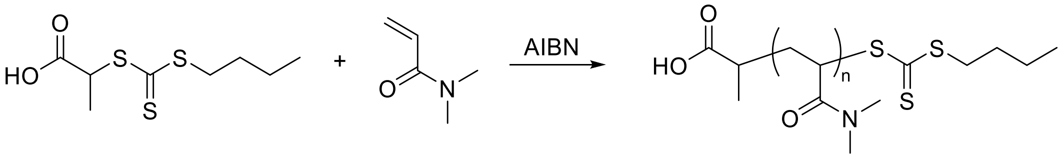

The polymerization was carried out as previously reported.[1] For a degree of polymerization of 25 repeating units, *N*,*N*-dimethylacrylamide (519 µL, 5.04 mmol), AIBN (6.6 mg, 0.04 mmol) and PABTC (48.1 mg, 0.20 mmol) were dissolved in 2 mL DMF and the reaction mixture was subjected to three freeze-pump-thaw cycles. The solution was stirred at 80 °C for 3 h and poly(*N*,*N*-dimethylacrylamide) (pDMA) was isolated by repeated precipitation in cold diethyl ether as a yellow powder.

In analogy a pDMA polymer with a degree of polymerization of 50 repeating units could be prepared by increasing the *N*,*N*-dimethylacrylamide : PABTC ratio from 25 : 1 to 50 : 1.

^{1}H NMR (400 MHz, CD_3OD, *δ*): 5.21 (m, 1H, –S–C*H*–CON(CH_3)$_2$), 3.43 (m, 2H, –S–CH_2–CH_2–), 3.22 – 2.86 (m, 192H, –N–(CH_3)$_2$), 2.82 – 2.18 (m, 32H, –C*H*–CON(CH_3)$_2$), 1.90 – 1.23 (m, 64H, –CH_2–CH–CONR_2), 1.13 (t, 3H, –CH–CH_3), 0.95 (t, 3H, –CH_2–CH_3).

SEC (HFIP; PMMA standard):

- for pDMA with a degree of 25: M_n = 3297 g mol^{-1}; M_w = 3949 g mol^{-1}; PDI = 1.11.
- for pDMA with a degree of 50: M_n = 6970 g mol^{-1}; M_w = 7901 g mol^{-1}; PDI = 1.13.

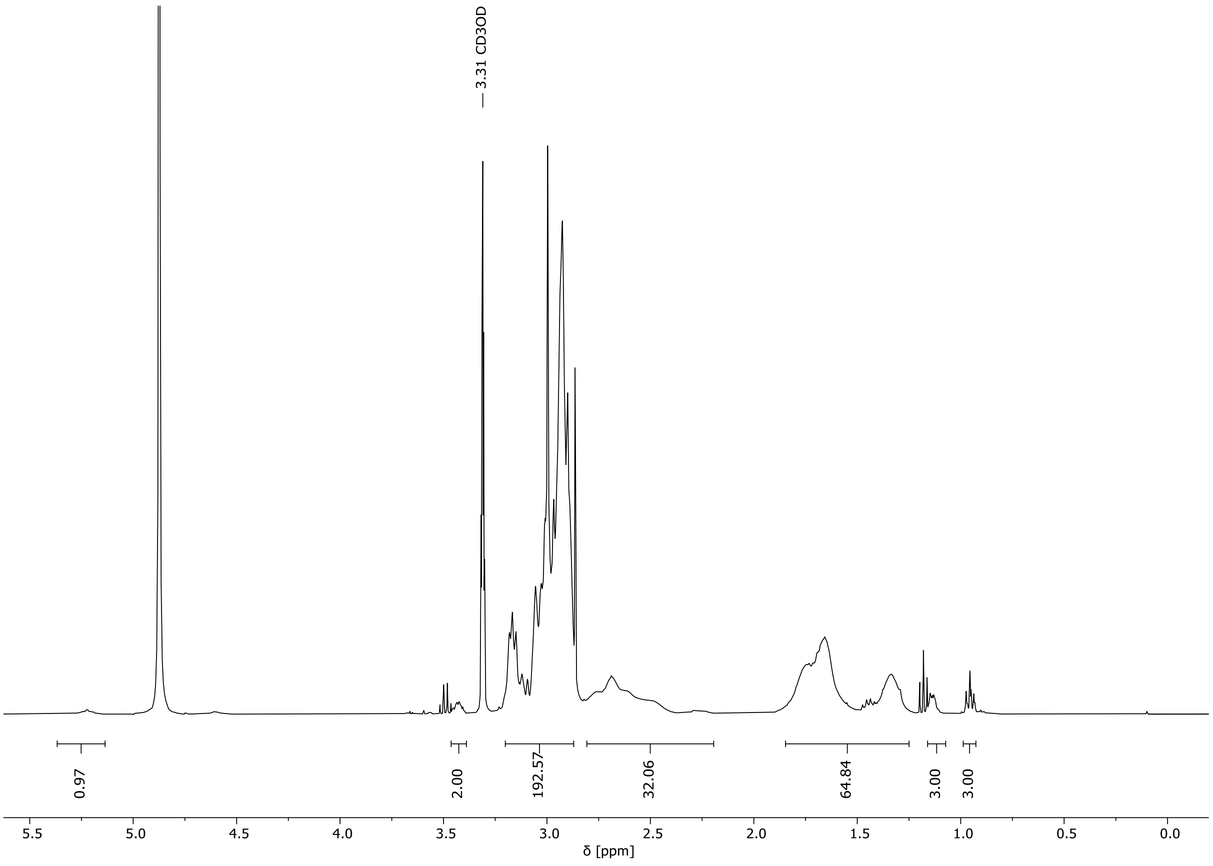

Figure S6: ^{1}H NMR (300 MHz, CD_3OD) of pDMA.

2-(pyridin-2-yldisulfanyl)ethanol

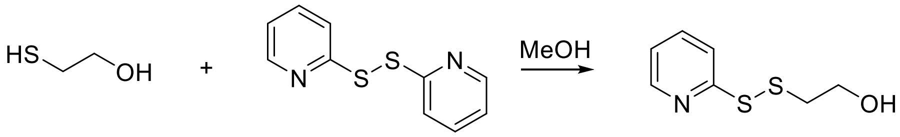

The synthesis was adapted from literature.[2] 2-Mercaptoethanol (0.52 mL, 7.4 mmol) was added dropwise to a solution of 2,2'-dipyridyl disulfide (4.92 g, 22.3 mmol) in 25 mL degassed MeOH. The reaction mixture turned yellow immediately and was stirred at room temperature for 1.5 h. The product was isolated by silica gel chromatography (chloroform/ethyl acetate = 4/1) as a yellow oil (1.21 g, 87%).

R_f=0.60 (chloroform/ethyl acetate = 2/1).

^{1}H NMR (400 MHz, $CDCl_3$): δ [ppm] = 8.54–8.50 (m, 1H, –C*H*=N–), 7.62–7.56 (m, 1H, =C*H*–CH=CH–N=), 7.43–7.38 (m, 1H, –CSR=C*H*–), 7.18–7.13 (m, 1H, –C*H*=CH–N=), 3.82–3.78 (m, 2H, –C*H*2–OH), 2.98–2.93 (m, 2H, –S–C*H*2–).

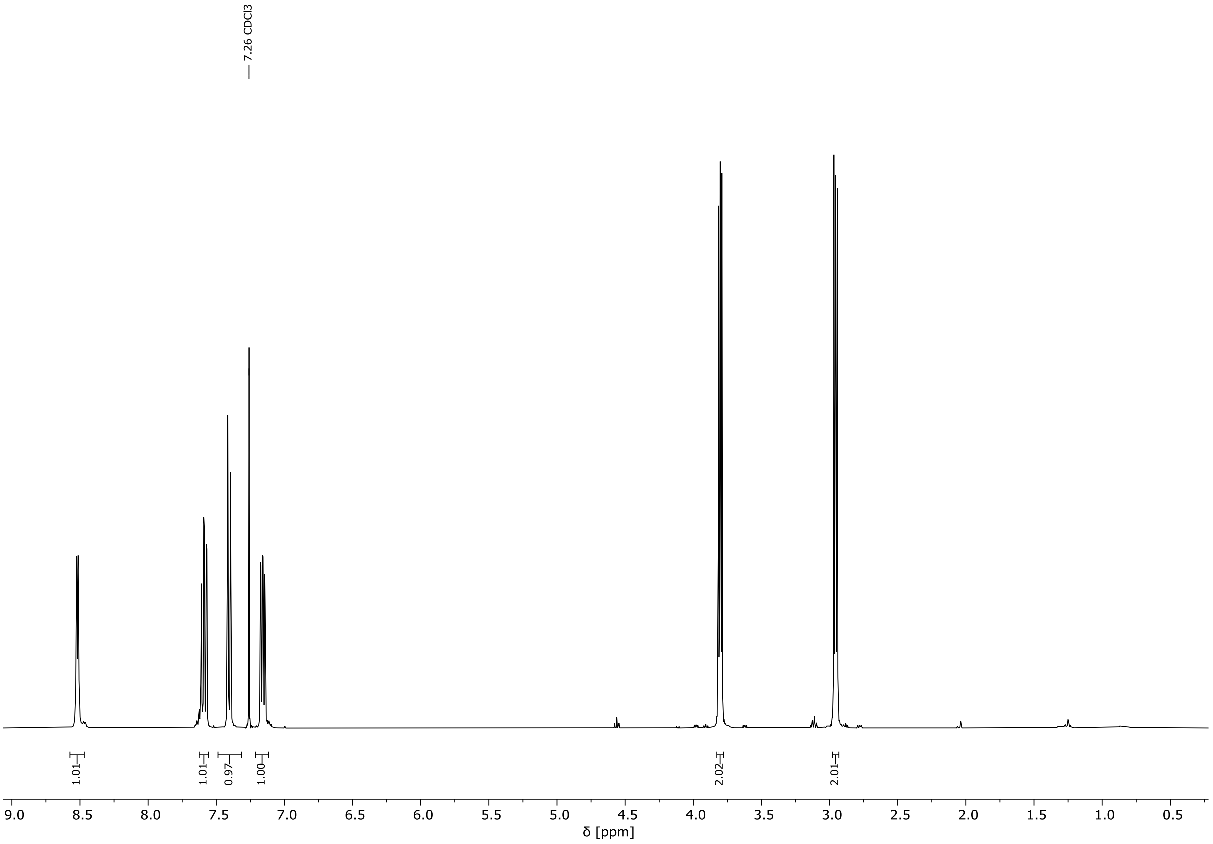

Figure S7: ^{1}H NMR (400 MHz, $CDCl_3$) of 2-(pyridin-2-yldisulfanyl)ethanol.

4-nitrophenyl (2-(pyridin-2-yldisulfanyl)ethyl) carbonate

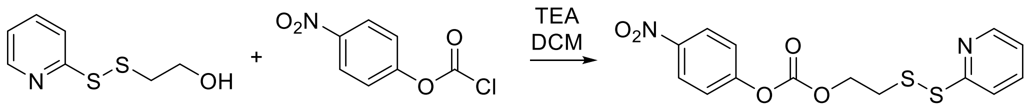

Triethylamine (1.1 mL, 7.8 mmol) and 2-(pyridin-2-yldisulfaneyl)ethan-1-ol (0.49 g, 2.6 mmol) were dissolved in 15 mL dry DCM and *p*-nitrophenyl chloroformate (0.63 g, 3.1 mmol) in 3 mL dry DCM was added dropwise over 1.5 h under vigorous stirring. After 24 h of stirring, another 0.1 eq of 4-nitrophenyl chloroformate (0.05 g, 0.3 mmol) were added and stirring was continued for 24 h. The product was isolated by silica gel chromatography (chloroform/methanol = 100/1) as a yellowish oil (0.92 g, 99%).

R_f=0.50 (petroleum ether/ethyl acetate = 3/1).

^{1}H NMR (400 MHz, $CDCl_3$): δ [ppm] = 8.53–8.49 (m, 1H, –C*H*=N–), 8.31–8.25 (m, 2H, –CNO_2=C*H*–), 7.72–7.64 (m, 2H, =C*H*–CH=CH–N= and –CSR=C*H*–), 7.41–7.35 (m, 2H, –C*H*=COR–), 7.18–7.12 (m, 1H, –C*H*=CH–N=), 4.57 (t, *J* = 6.4 Hz, 2H, –CH_2–O–), 3.17 (t, *J* = 6.4 Hz, 2H, –S–CH_2–).

^{13}C NMR (100 MHz, $CDCl_3$): δ [ppm] = 159.14, 155.50, 152.34, 149.76 (–*C*H=N–), 145.60, 137.51 (=*C*H–CH=CH–N=), 125.48 (–CNO_2=*C*H–), 121.89 (–*C*H=C–O–), 121.34 (–*C*H=CH–N=), 120.48 (–CSR=*C*H–), 66.81 (–*C*H_2–O–), 36.94 (–S–*C*H_2–).

ESI-MS (m/z): $[M+H]^+$ 353.03, found 353.03; $[M+Na]^+$ 375.01, found 375.01.

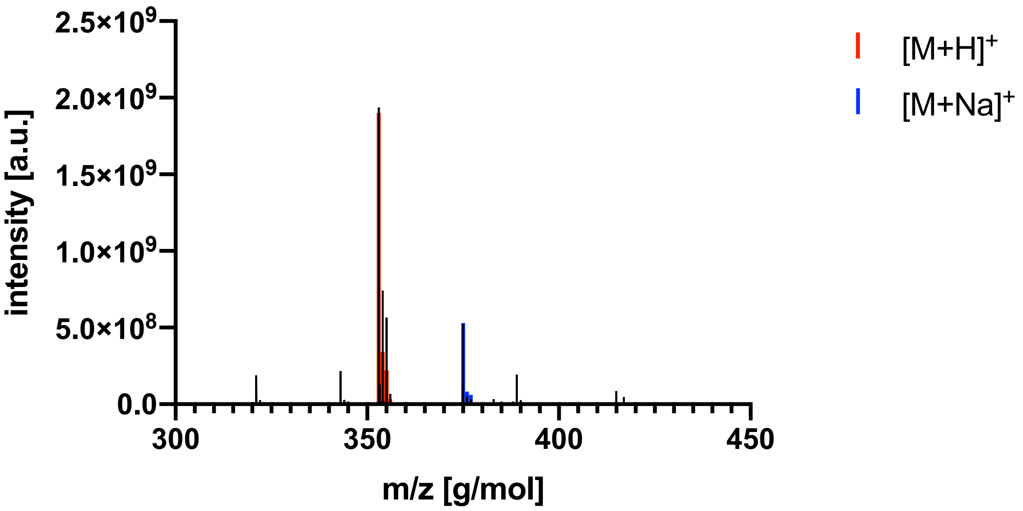

Figure S8: ESI-MS of 4-nitrophenyl (2-(pyridin-2-yldisulfanyl)ethyl) carbonate.

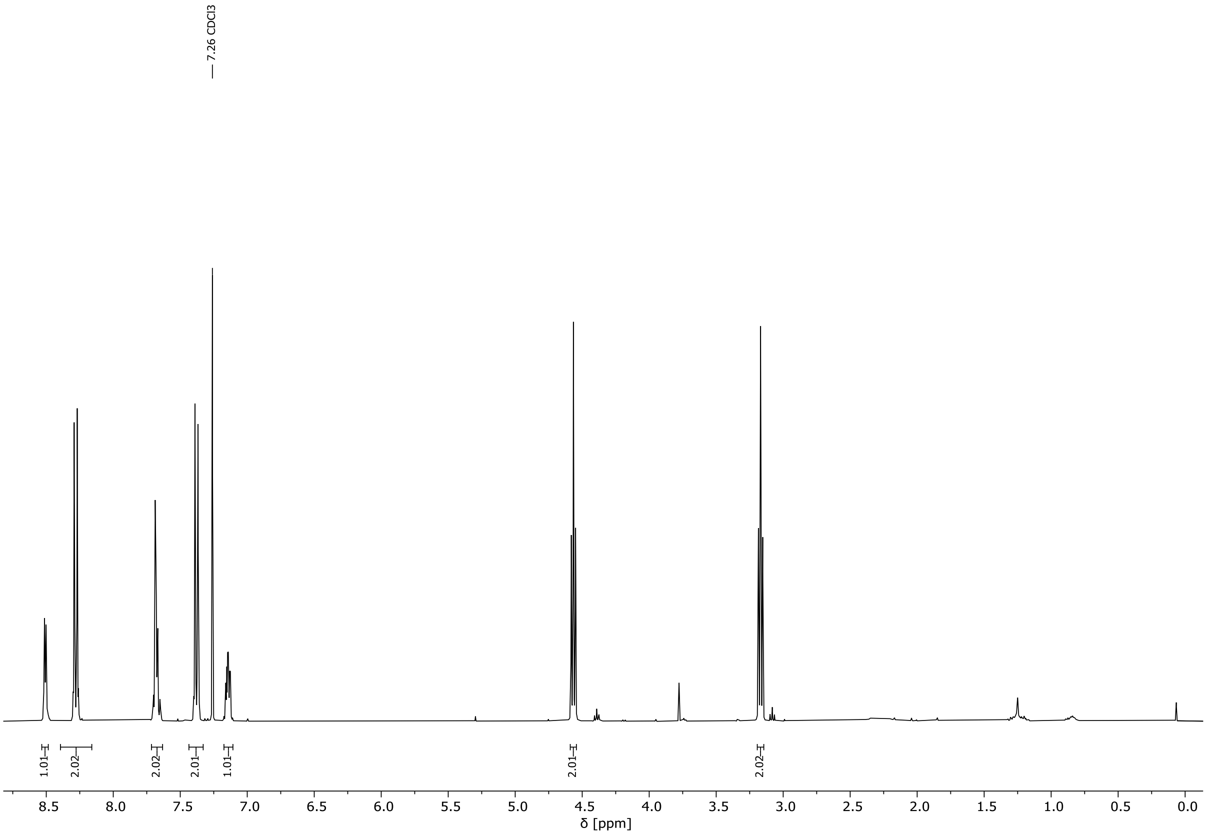

Figure S9: ^{1}H NMR (400 MHz, $CDCl_3$) of 4-nitrophenyl (2-(pyridin-2-yldisulfanyl)ethyl) carbonate.

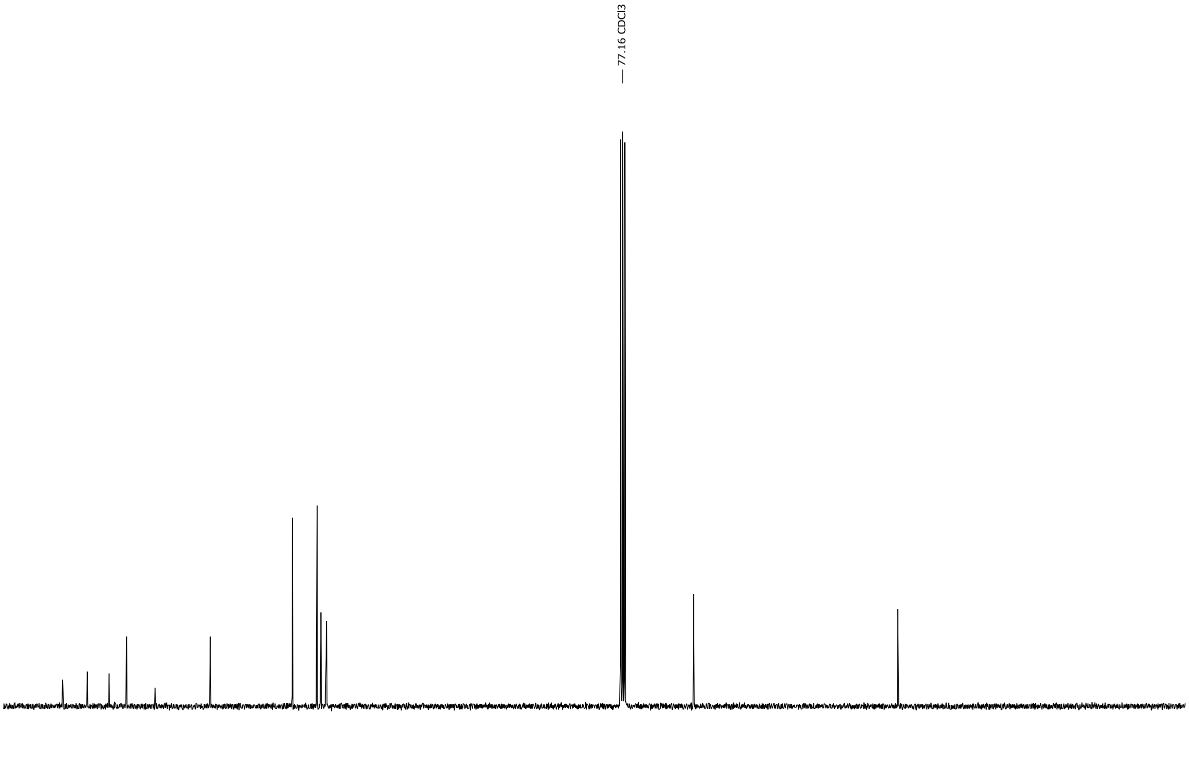

Figure S10: ^{13}C NMR (100 MHz, $CDCl_3$) of 4-nitrophenyl (2-(pyridin-2-yldisulfanyl)ethyl) carbonate.

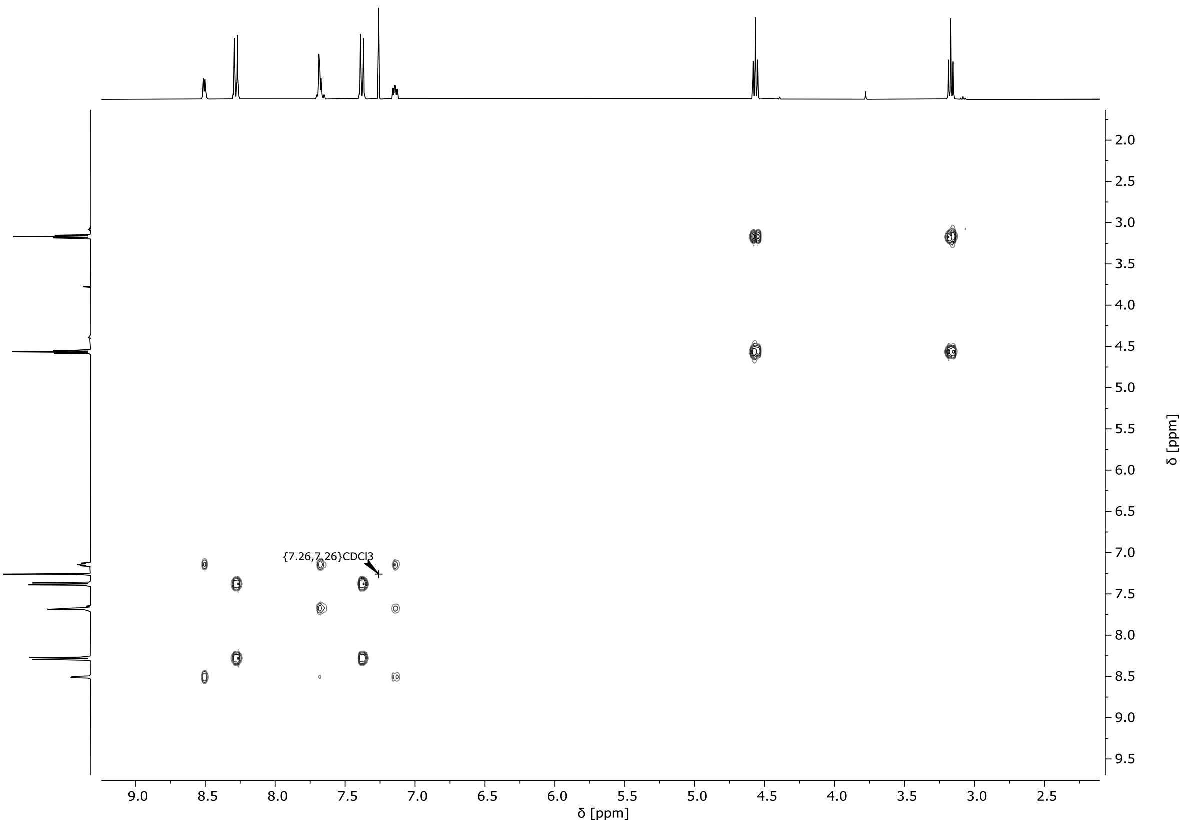

Figure S11: $^{1}H,^{1}H$ COSY spectrum of 4-nitrophenyl (2-(pyridin-2-yldisulfanyl)ethyl) carbonate.

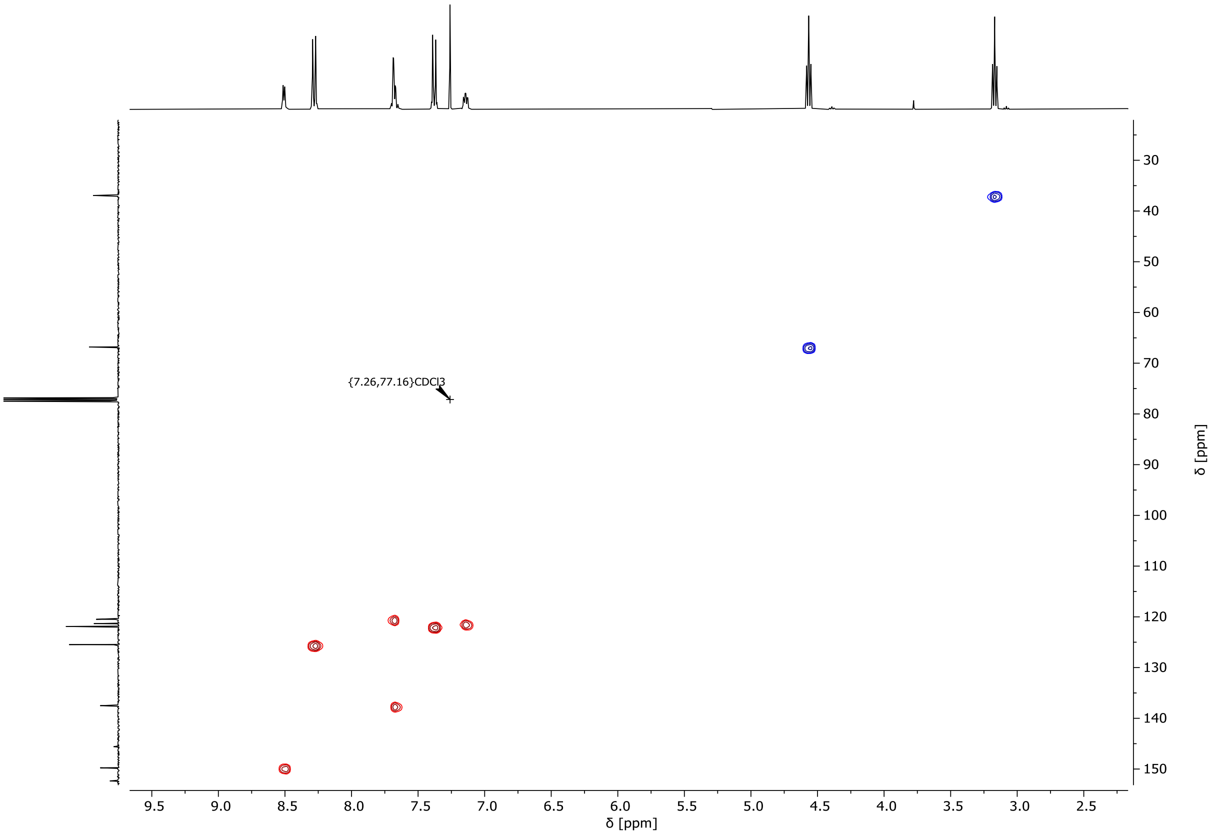

Figure S12: $^{1}H,^{13}C$ HSQC spectrum of 4-nitrophenyl (2-(pyridin-2-yldisulfanyl)ethyl) carbonate.

2-(pyridin-2-yldisulfaneyl)ethyl benzylcarbamate

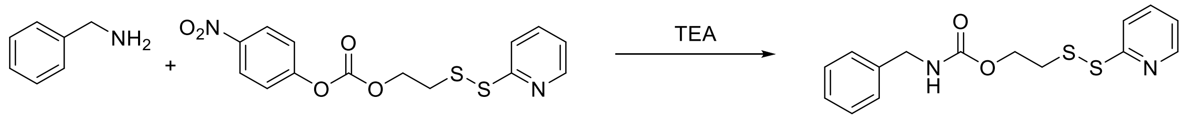

Benzyl amine (47.3 µL, 434 µmol) and triethylamine (90.8 µL, 651 µmol) were added to a solution of 4-nitrophenyl (2-(pyridin-2-yldisulfanyl)ethyl) carbonate (76.5 mg, 217 µmol) in 1.5 mL dry DCM and was stirred at rt overnight. The reaction mixture was applied on basic aluminium oxide and the product was isolated with a subsequent column chromatography (cyclohexane/ethyl acetate = 3/1) as a colorless solid (64.0 mg, 92%).

R_f=0.30 (petroleum ether/ethyl acetate = 3/1).

^{1}H NMR (300 MHz, $CDCl_3$): δ [ppm] = 8.44 (d, J = 4.8 Hz, 1H, –N=C*H*–), 7.68 (d, J = 8.1 Hz, 1H, –S–CR=C*H*–), 7.60 (t, J = 7.3 Hz, 1H, –S–CR=CH–C*H*=), 7.41 – 7.22 (m, 5H, $-C_6H_5$), 7.12 – 7.02 (m, 1H, –N=CH–C*H*=), 5.40 – 5.02 (m, 1H, –N*H*–), 4.42 – 4.26 (m, 4H, $-O-CH_2-$ and $-CH_2-C_6H_5$), 3.04 (t, J = 6.3 Hz, 2H, $-S-CH_2-$).

^{13}C DEPT NMR (75 MHz, $CDCl_3$): δ [ppm] = 149.71, 137.03, 128.71, 127.57, 120.81, 119.82, 62.83, 45.10, 38.02.

ESI-MS (m/z): $[M+H]^+$ 321.07, found 321.09; $[M+Na]^+$ 343.05, found 343.06.

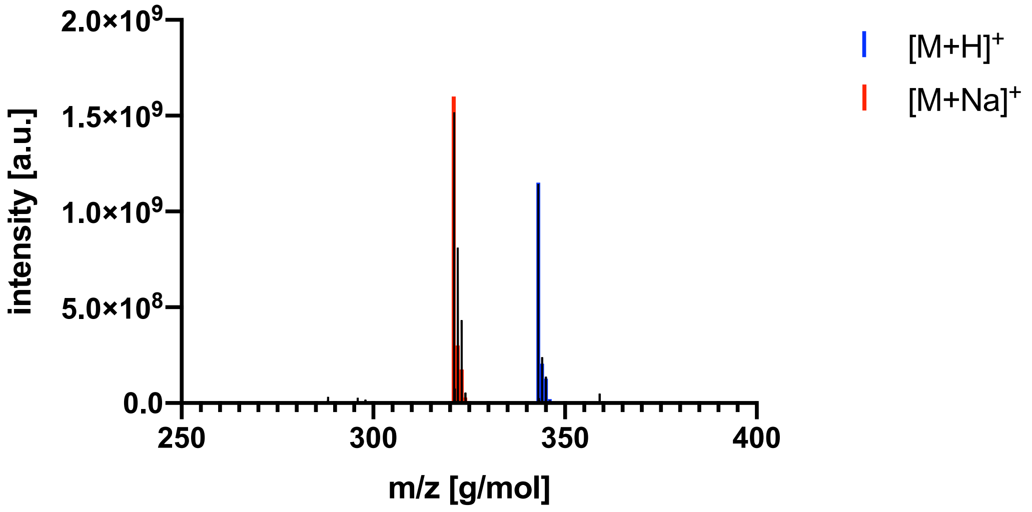

Figure S13: ESI-MS of 2-(pyridin-2-yldisulfaneyl)ethyl benzylcarbamate.

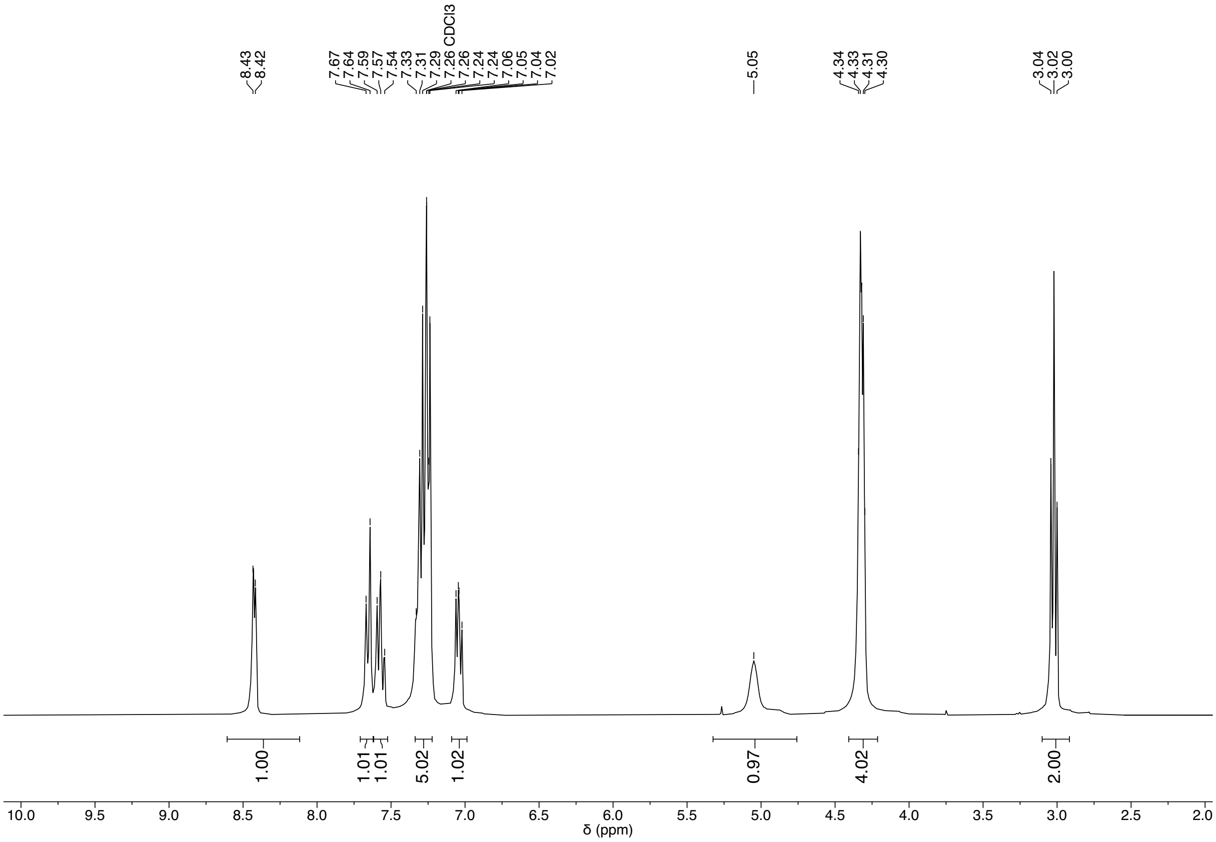

Figure S14: ^{1}H NMR (300 MHz, $CDCl_3$) of 2-(pyridin-2-yldisulfaneyl)ethyl benzylcarbamate.

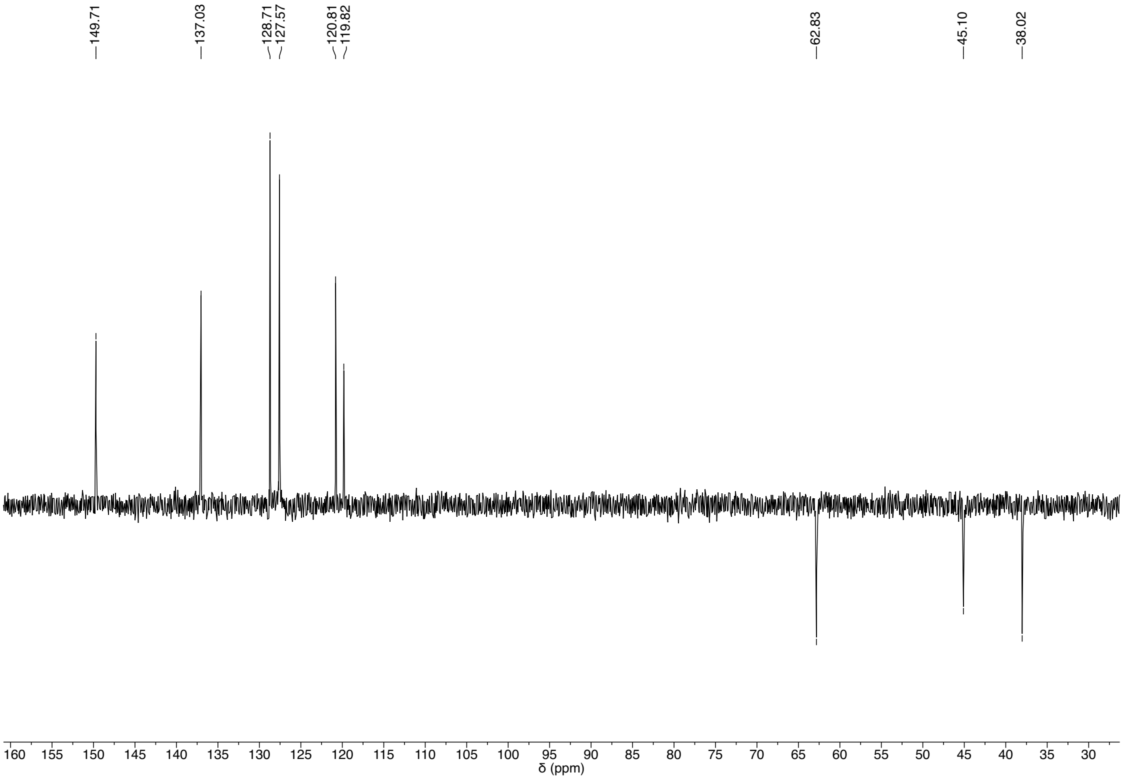

Figure S15: ^{13}C DEPT NMR (75 MHz, $CDCl_3$) of 2-(pyridin-2-yldisulfaneyl)ethyl benzylcarbamate.

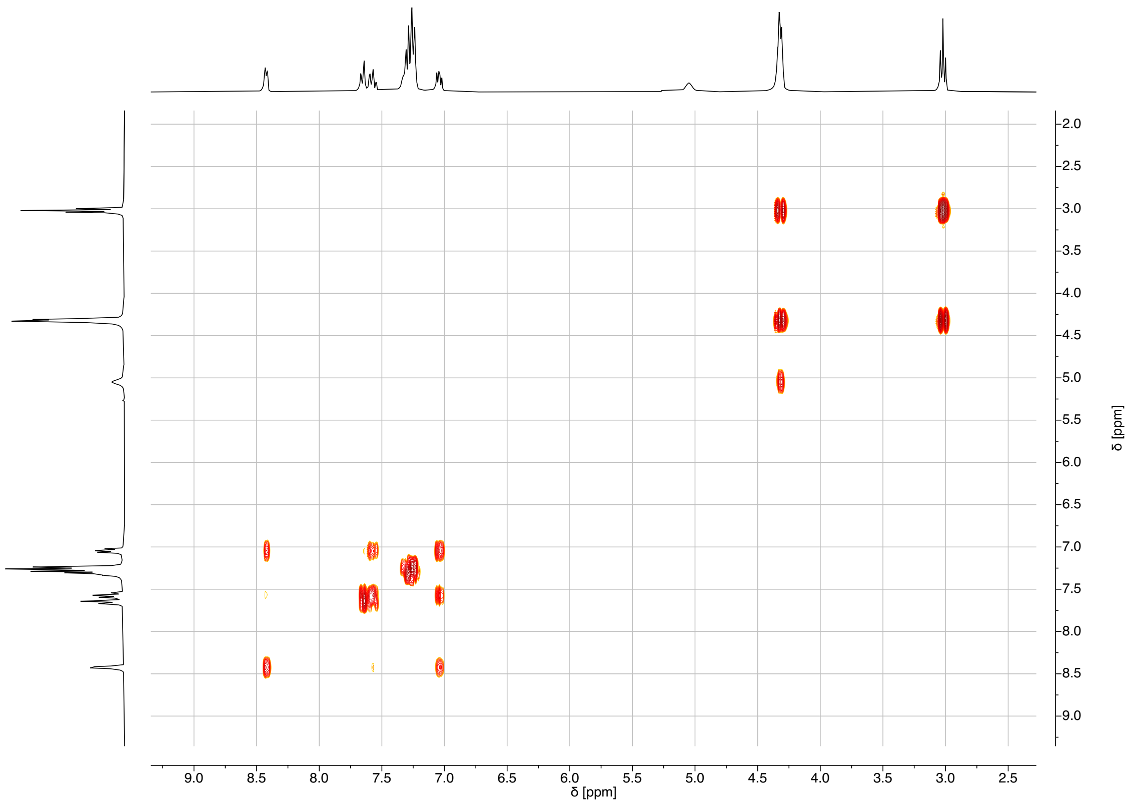

Figure S16: ^{1}H,^{1}H COSY spectrum of 2-(pyridin-2-yldisulfaneyl)ethyl benzylcarbamate.

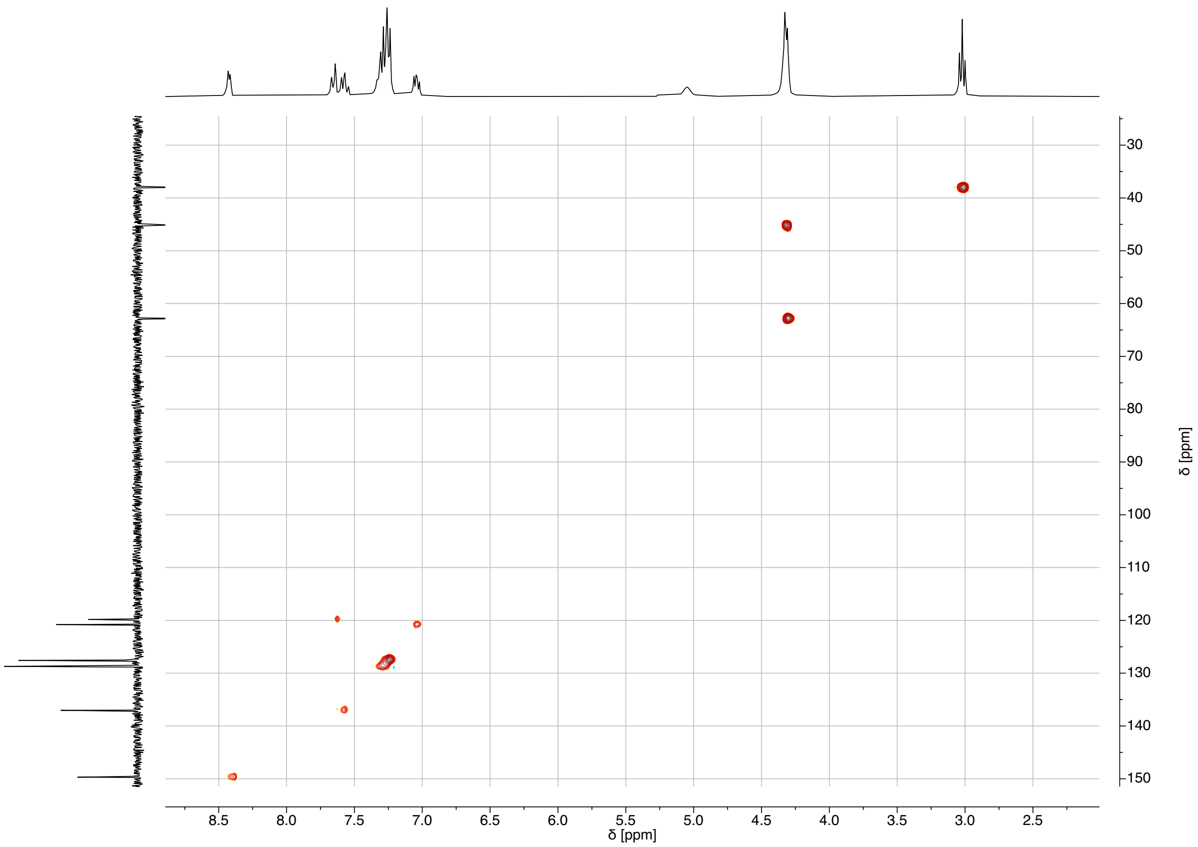

Figure S17: ^{1}H,^{13}C HSQC spectrum of 2-(pyridin-2-yldisulfaneyl)ethyl benzylcarbamate.

pDMA-NHBz derived from 2-(pyridin-2-yldisulfaneyl)ethyl benzylcarbamate

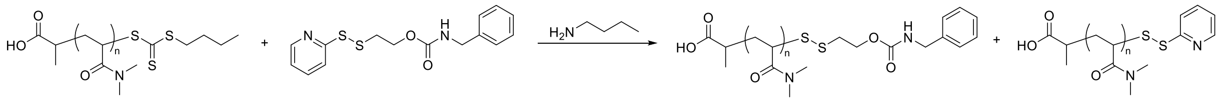

pDMA (10.0 mg, 4.1 µmol, DP 25), *n*-butylamine (2.04 µL, 20.7 µmol) and 2-(pyridin-2-yldisulfaneyl)ethyl benzylcarbamate (6.6 mg, 20.7 µmol) were dissolved in 0.3 mL dry DMSO and stirred overnight at room temperature. The colorless polymer was isolated by repeated precipitation in diethyl ether.

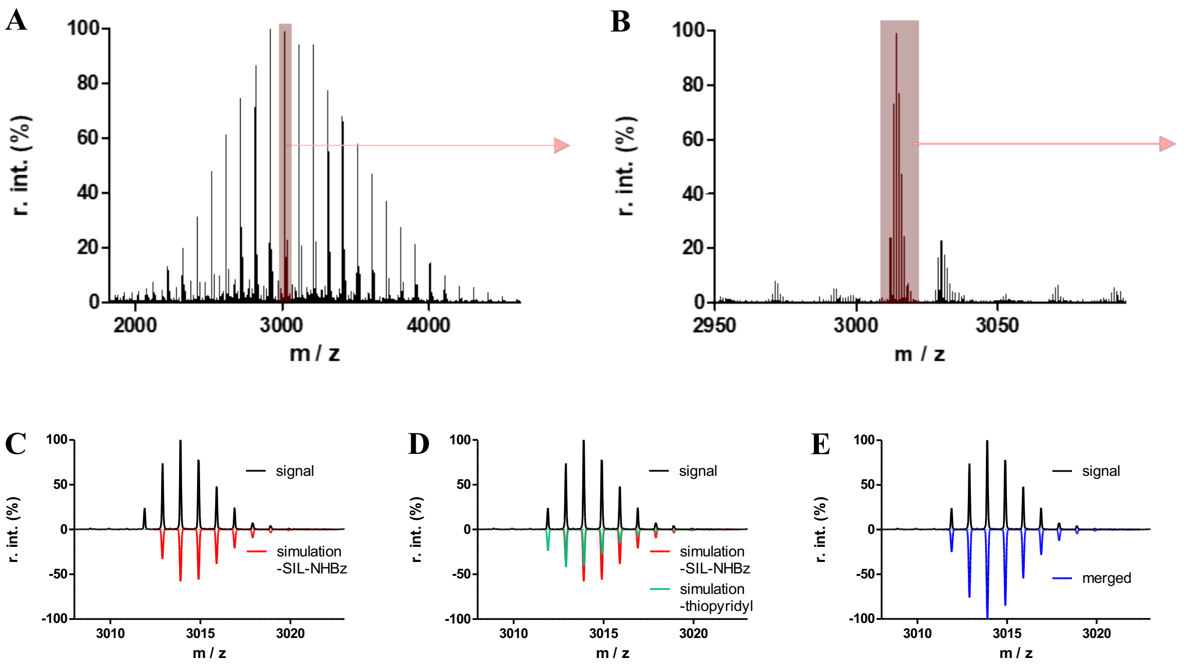

Figure S18: MALDI-ToF MS data of pDMA-NHBz derived from 2-(pyridin-2-yldisulfaneyl)ethyl benzylcarbamate. **A:** Full polymer mass range. **B:** Zoomed mass range of DP with highest relative intensity. **C:** Comparison of the signal of DP with highest relative intensity and corresponding simulated isotope pattern of the expected product. **D:** Comparison of the signal of DP with highest relative intensity and corresponding simulated isotope patterns of the expected product SIL-NHBz and the thiopyridyl byproduct. Both polymers own nearly identical overlapping isotopic distributions deviating by only one mass unit. **E:** Comparison of the signal of DP with highest relative intensity and merged corresponding simulated isotope patterns of the expected product and its byproduct.

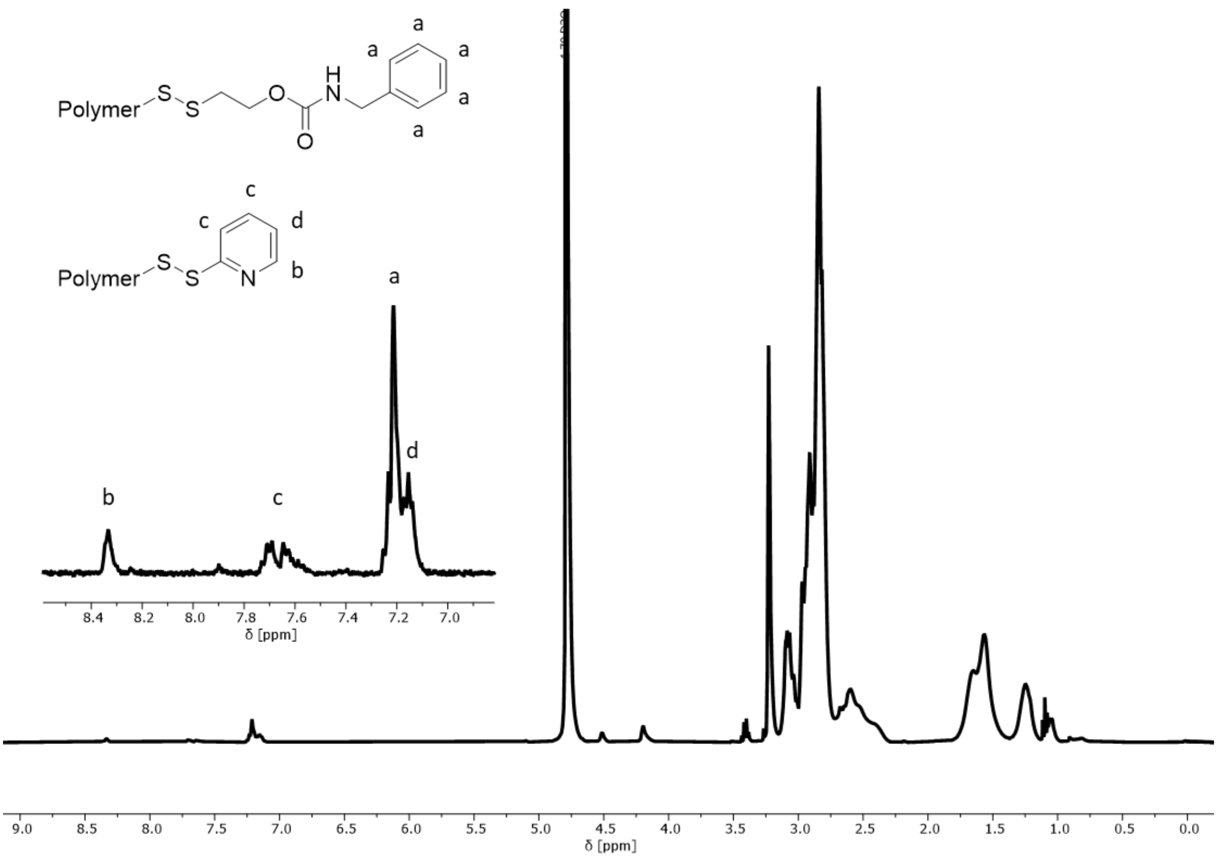

Figure S19: ^{1}H NMR (400 MHz, D_2O) of pDMA-NHBz derived from 2-(pyridin-2-yldisulfaneyl)ethyl benzylcarbamate. Zoomed region demonstrates assignment of aromatic proton signals of benzyl amine as well as still remaining pyridine thiol moiety.

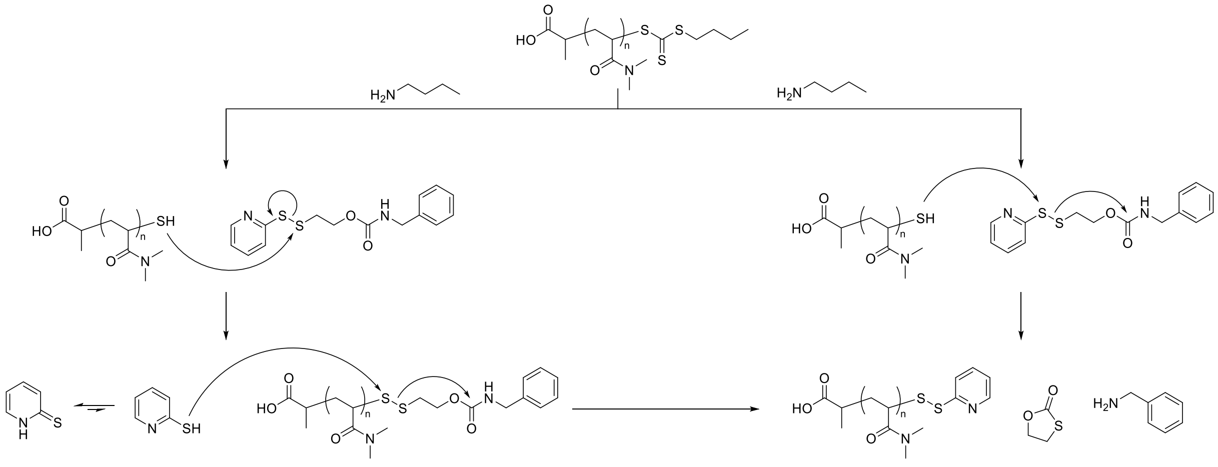

Figure S20: Estimated mechanisms for formation of self-immolative motif and pyridine thiol capped pDMA.

Potassium 4-methylbenzenesulfinate

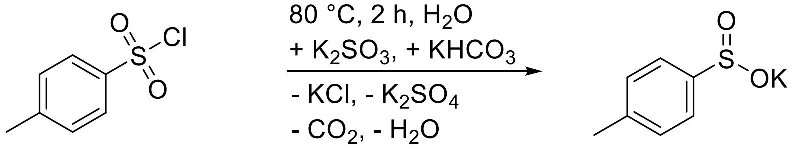

3-toluenesulfonyl chloride (28.59 g, 150 mmol) was added in small portions to a solution of potassium bicarbonate (30.03 g, 300 mmol) and potassium sulfite (79.14 g, 450 mmol) in water (118 mL) at 80 °C. The mixture was further stirred at the same temperature for 2 h, afterwards water was removed in vacuo at 60 °C. The resulting solid was suspended in 300 mL methanol and filtered. Evaporation of methanol gave potassium 4-methylbenzenesulfinate (26.44 g (94%), 136.10 mmol, 91%) as a colorless solid.

^{1}H NMR (300 MHz, D_2O): δ [ppm] = 7.54 (d, J = 6 Hz, 2H, Ar-H); 7.37 (d, J = 6 Hz, 2H, Ar-H); 2.39 (s, 3H, -CH_3).

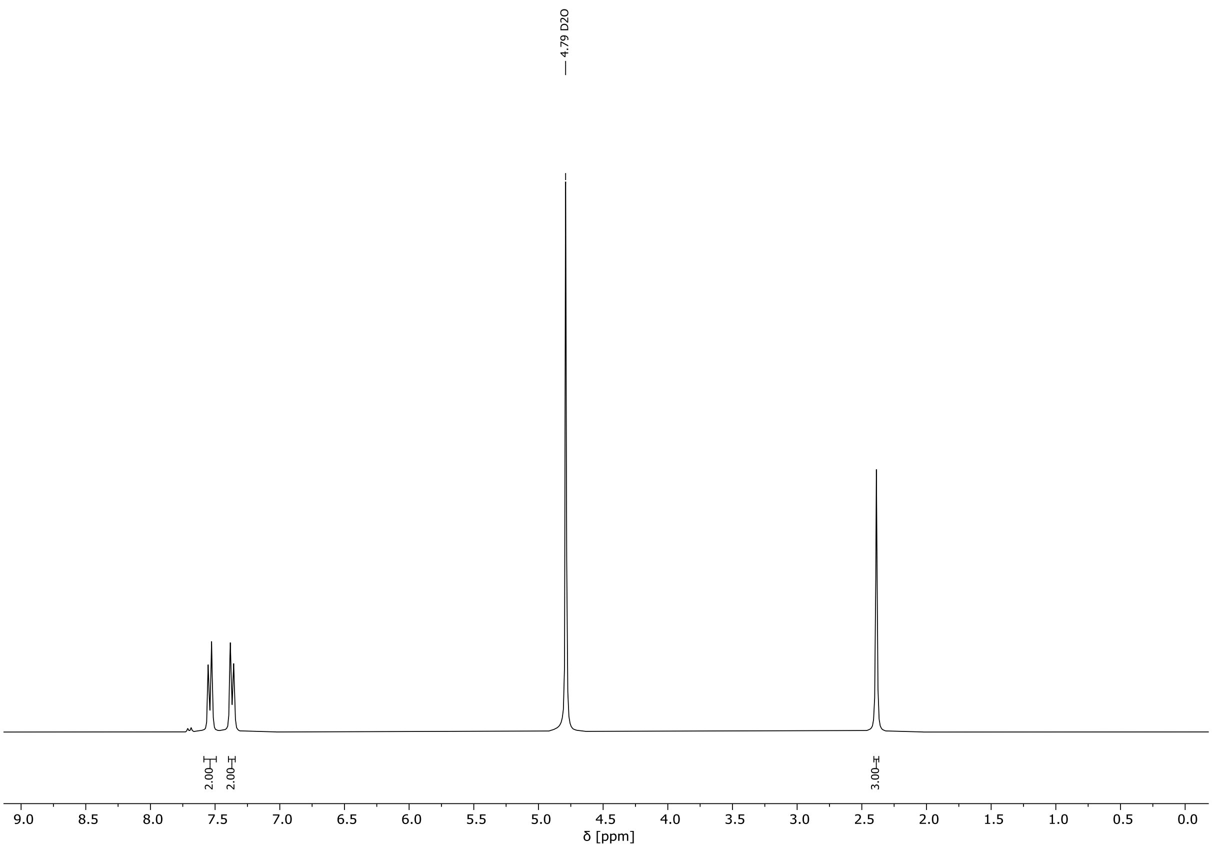

Figure S21: ^{1}H NMR (300 MHz, D_2O) of potassium 4-methylbenzenesulfinate.

Potassium 4-methylbenzenesulfonothioate

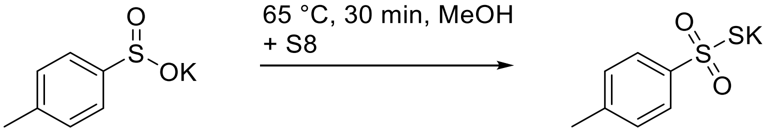

Potassium 4-methylbenzenesulfinate (10.33 g, 50 mmol, 1.00 eq) and sulfur (1.57 g, 49 mmol, 0.98 eq) were suspended in methanol (300 mL). The yellow suspension was refluxed for 30 min to a clear solution, cooled down and evaporated. The colorless residue was triturated with ethanol and again evaporated to yield potassium 4-methylbenzenesulfonothioate (11.30 g (97%), 48.44 mmol, 97%) as a colorless solid.

^{1}H NMR (300 MHz, D_2O): δ [ppm] = 7.82 (d, J = 9 Hz, 2H, Ar-H); 7.38 (d, J = 9 Hz, 2H, Ar-H); 2.41 (s, 3H, -CH_3).

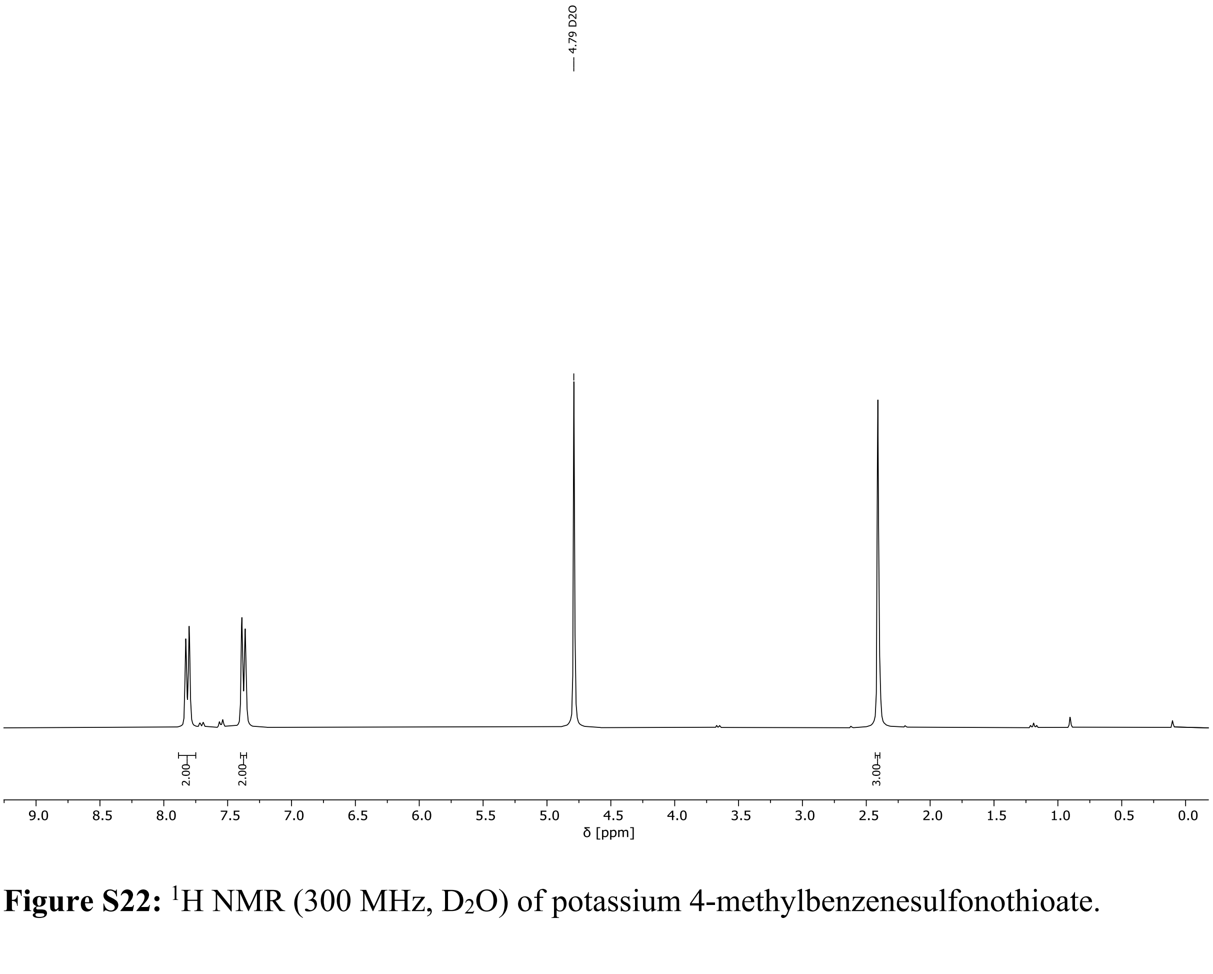

Figure S22: ^{1}H NMR (300 MHz, D_2O) of potassium 4-methylbenzenesulfonothioate.

2-bromoethyl (4-nitrophenyl) carbonate

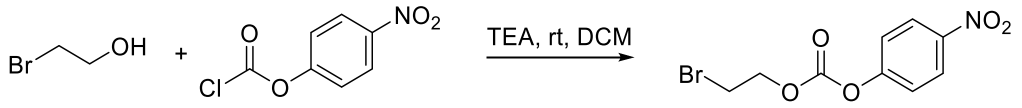

Bromoethanol (1.69 g, 13.5 mmol) and triethylamine (2.27 mL, 16.3 mmol) were dissolved in 50 mL dry DCM and *p*-nitrophenyl chloroformate (3.00 g, 14.9 mmol) in 10 mL dry DCM were added dropwise. The solution was stirred for four days at room temperature and the solvent was removed under reduced pressure. The residue was taken up in ethyl acetate and the precipitate was separated by filtration and washed with ethyl acetate. The solvent was removed and column chromatography (cyclohexane/ethyl acetate = 6/1) gave the product as a slightly yellow oil (3.22 g, 82%).

^{1}H NMR (300 MHz, $CDCl_3$): δ [ppm] = 8.29 (d, 2H), 7.40 (d, 2H), 4.60 (t, J = 6.0 Hz, 2H), 3.62 (t, J = 6.0 Hz, 2H).

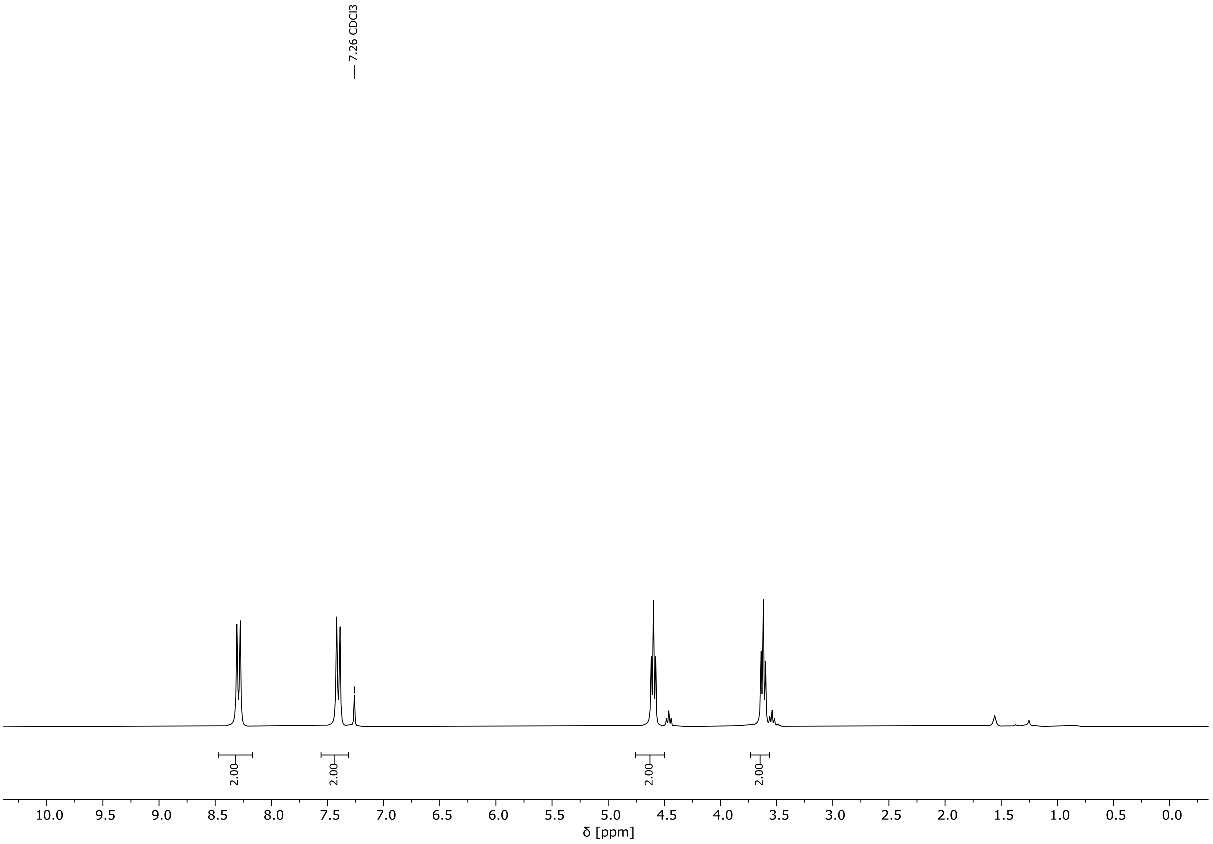

Figure S23: ^{1}H NMR (300 MHz, $CDCl_3$) of 2-bromoethyl (4-nitrophenyl) carbonate.

***S*-(2-(((4-nitrophenoxy)carbonyl)oxy)ethyl) 4-methylbenzenesulfonothioate**

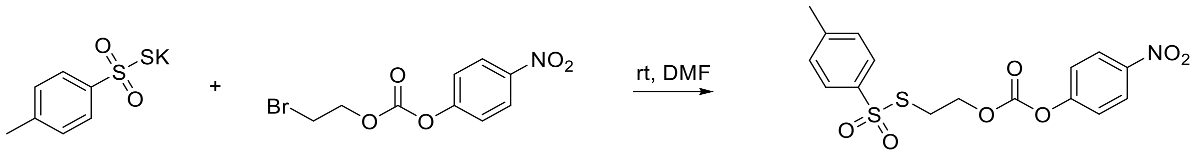

2-bromoethyl (4-nitrophenyl) carbonate (2.50 g, 8.6 mmol) and potassium 4-methylbenzene-sulfonothioate (2.73 g, 12.1 mmol) were dissolved in 30 mL dry DMF at room temperature until the bromide was consumed completely. The solvent was removed under reduced pressure and the residue was taken up in acetone. The precipitate was separated by filtration and washed with acetone. The solvent was removed and column chromatography (cyclohexane/ethyl acetate = 5/1) gave the product as a white solid (2.93 g, 86%).

^{1}H NMR (700 MHz, $CDCl_3$): δ [ppm] = 8.28 (d, 2H), 7.85 (d, 2H), 7.46 – 7.30 (m, 4H), 4.48 (t, J = 6.4 Hz, 2H), 3.34 (t, J = 6.4 Hz, 2H), 2.45 (s, 3H).

^{13}C NMR (175 MHz, $CDCl_3$): δ [ppm] = 155.41, 152.22, 145.69, 145.52, 141.67, 130.22, 127.28, 125.49, 121.90, 66.72, 33.88, 21.83.

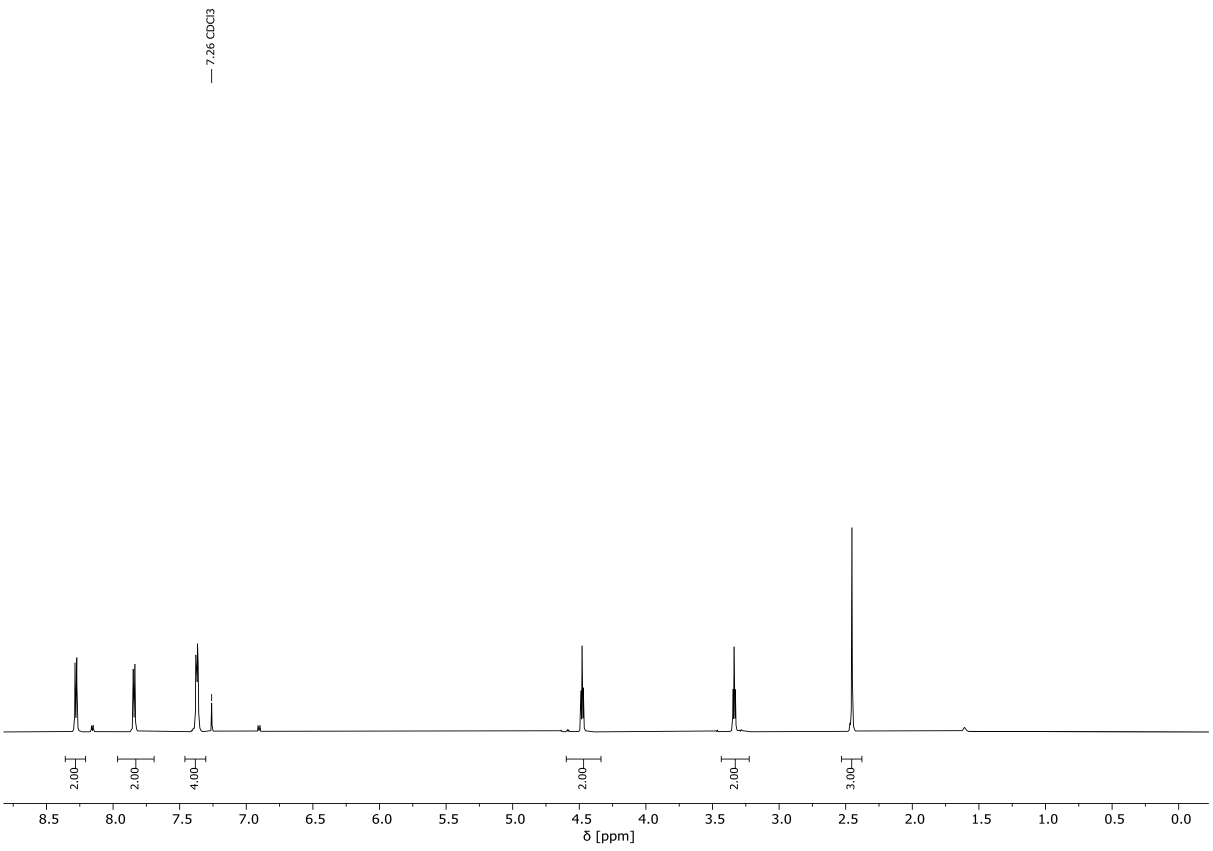

Figure S24: ^{1}H NMR (700 MHz, $CDCl_3$) of *S*-(2-(((4-nitrophenoxy)carbonyl)oxy)ethyl) 4-methylbenzenesulfonothioate.

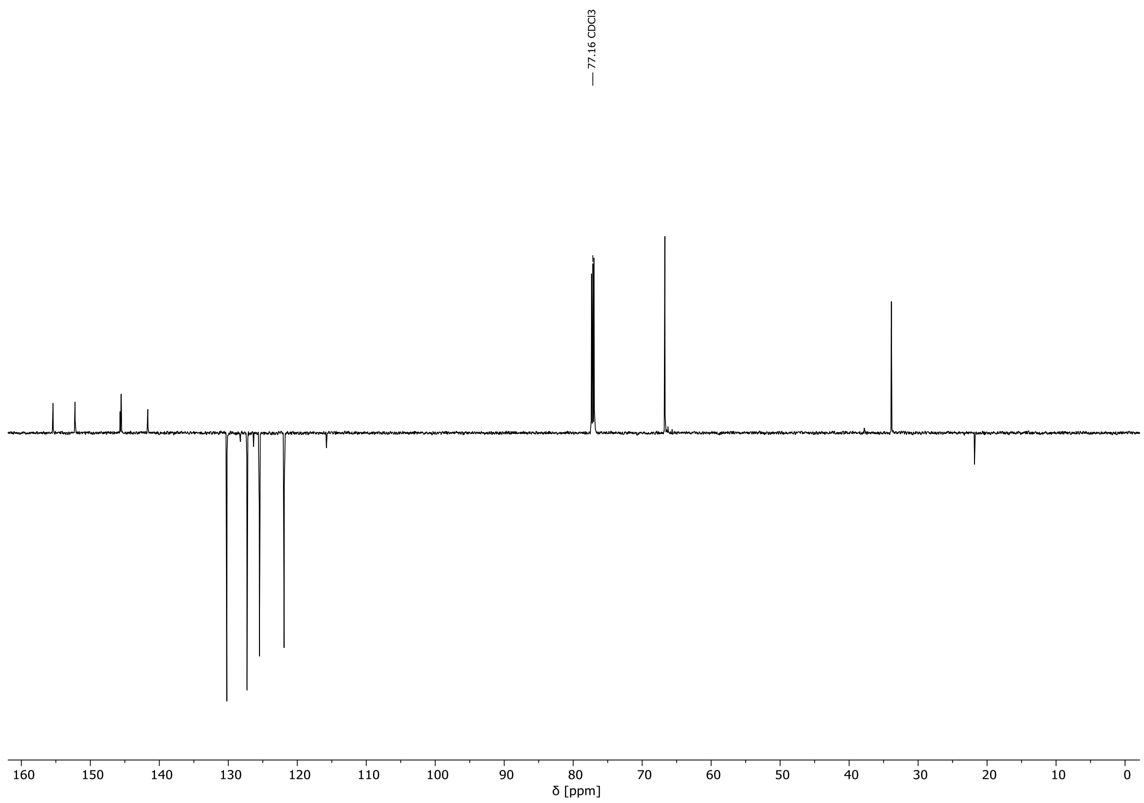

Figure S25: ^{13}C NMR (175 MHz, $CDCl_3$) of *S*-(2-(((4-nitrophenoxy)carbonyl)oxy)ethyl) 4-methylbenzenesulfonothioate.

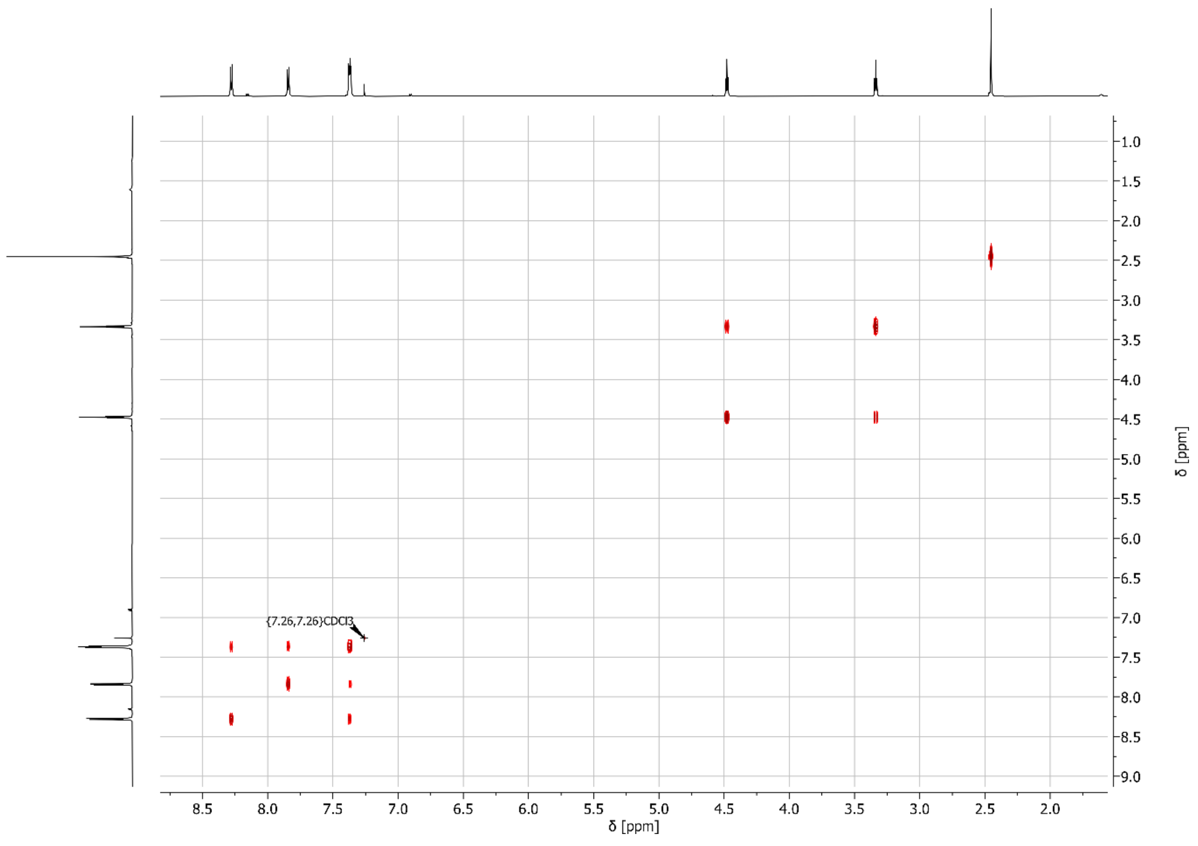

Figure S26: $^1H,^1H$ COSY spectrum of *S*-(2-(((4-nitrophenoxy)carbonyl)oxy)ethyl) 4-methyl-benzenesulfonothioate.

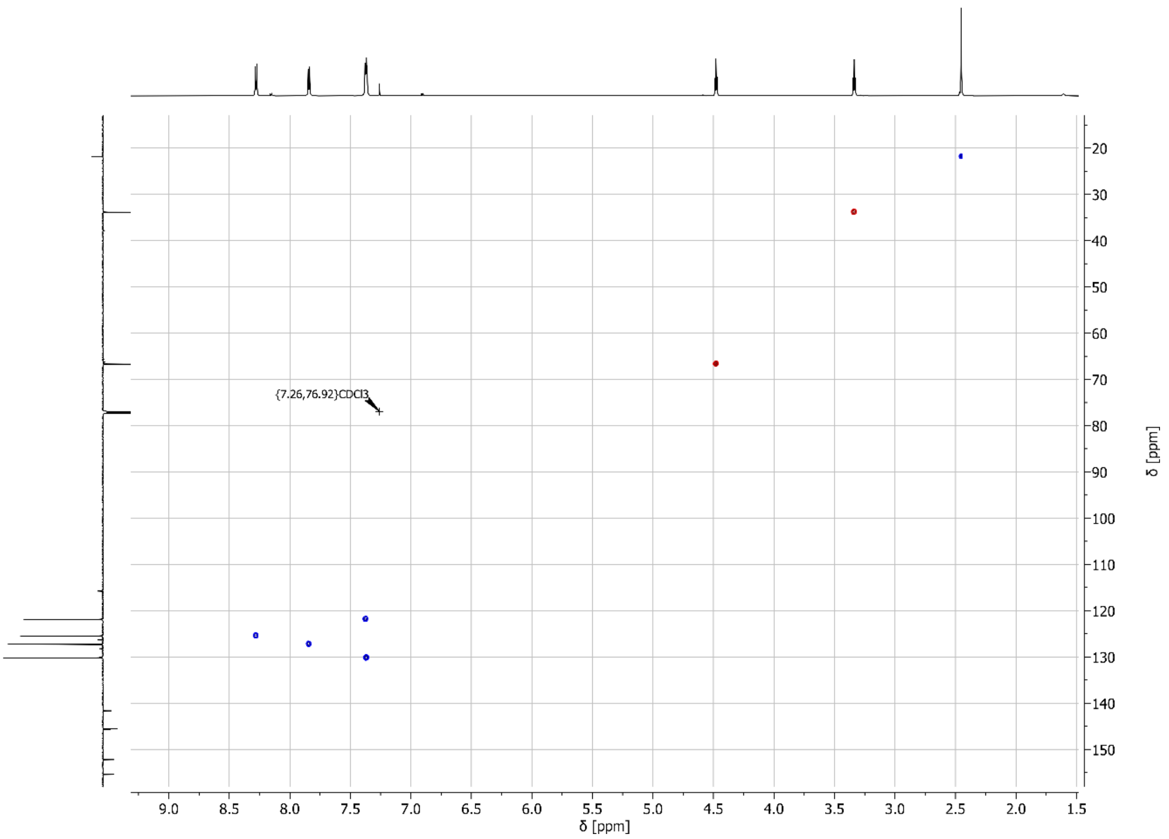

Figure S27: ^{1}H,^{13}C HSQC spectrum of *S*-(2-(((4-nitrophenoxy)carbonyl)oxy)ethyl) 4-methyl-benzenesulfonothioate.

S-(2-(((benzyloxy)carbonyl)oxy)ethyl) 4-methylbenzenesulfonothioate

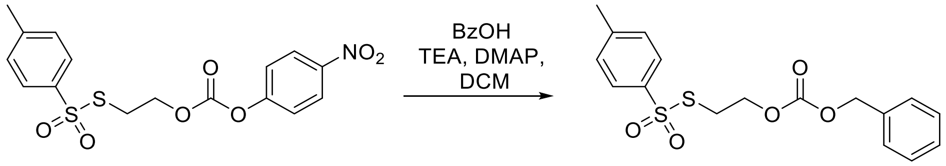

S-(2-(((4-nitrophenoxy)carbonyl)oxy)ethyl) 4-methylbenzenesulfonothioate (100 mg, 252 µmol), benzyl alcohol (39.2 µL, 377 µmol), 4-dimethylaminopyridine (3.1 mg, 25 µmol) and triethylamine (52.6 µL, 377 µmol) were dissolved in 1 mL dry DCM and were stirred at room temperature overnight. The solution was diluted with additional DCM and extracted four times with water. The organic layer was dried with Na_2SO_4 and the solvent was removed under reduced pressure to give the product as a yellow oil (82.2 mg, 89%).

R_f=0.60 (cyclohexane/ethyl acetate = 2/1).

^{1}H NMR (400 MHz, $CDCl_3$): δ [ppm] = 7.90 – 7.74 (m, 2H), 7.38 – 7.35 (m, 5H), 7.34 – 7.30 (m, 2H), 5.14 (s, 2H), 4.30 (t, J = 6.6 Hz, 2H), 3.24 (t, J = 6.6 Hz, 2H), 2.44 (s, 3H).

^{13}C NMR (100 MHz, $CDCl_3$): δ [ppm] = 154.58, 145.27, 141.65, 134.92, 130.09, 128.77, 128.73, 128.46, 127.18, 70.05, 65.46, 34.07, 21.76.

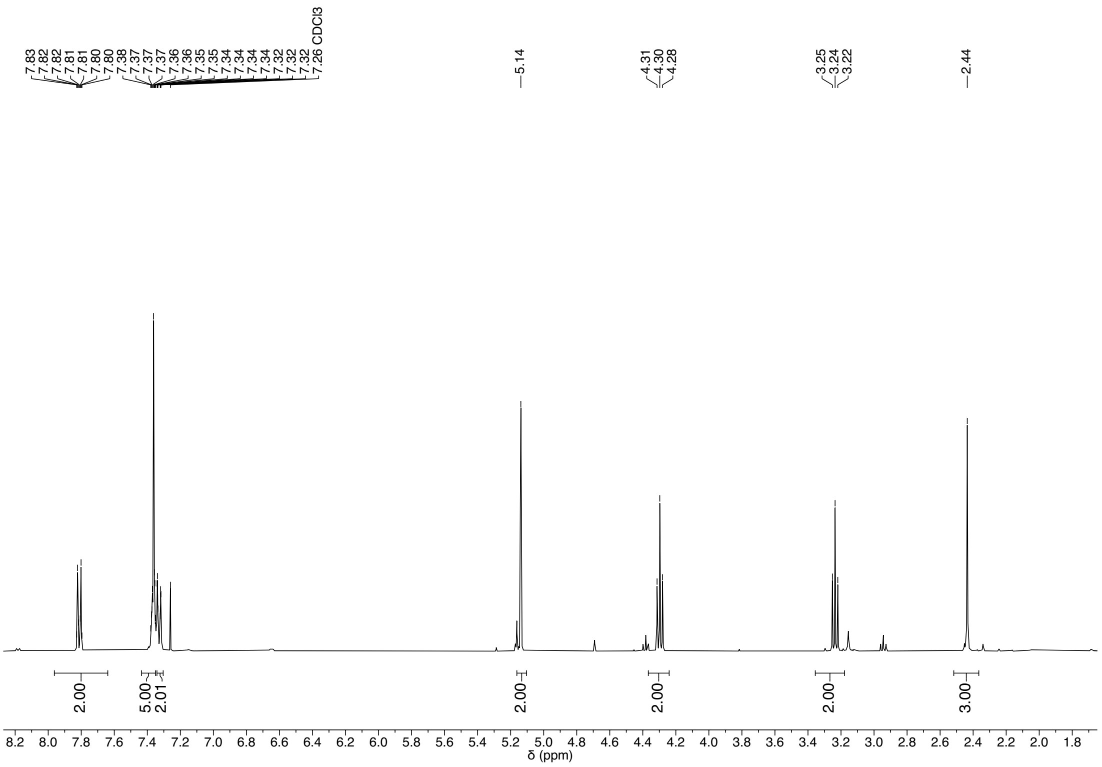

Figure S28: ^{1}H NMR (400 MHz, $CDCl_3$) of *S*-(2-(((benzyloxy)carbonyl)oxy)ethyl) 4-methylbenzenesulfonothioate.

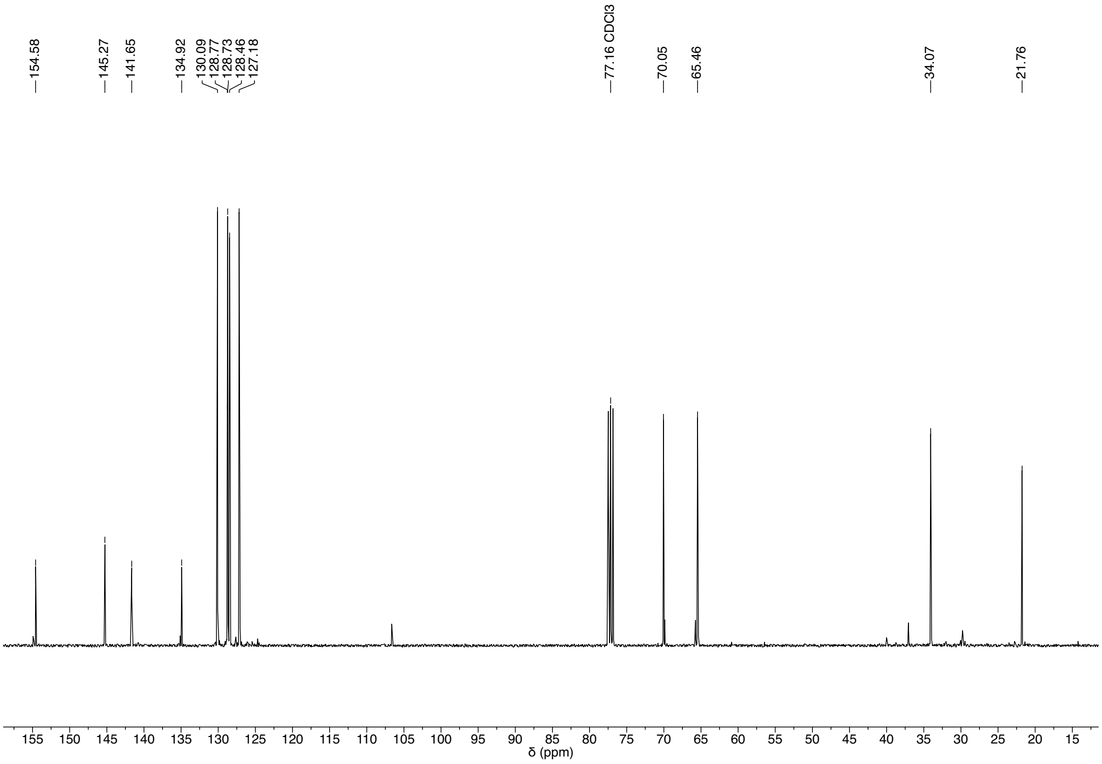

Figure S29: ^{13}C NMR (100 MHz, $CDCl_3$) of *S*-(2-(((benzyloxy)carbonyl)oxy)ethyl) 4-methylbenzenesulfonothioate.

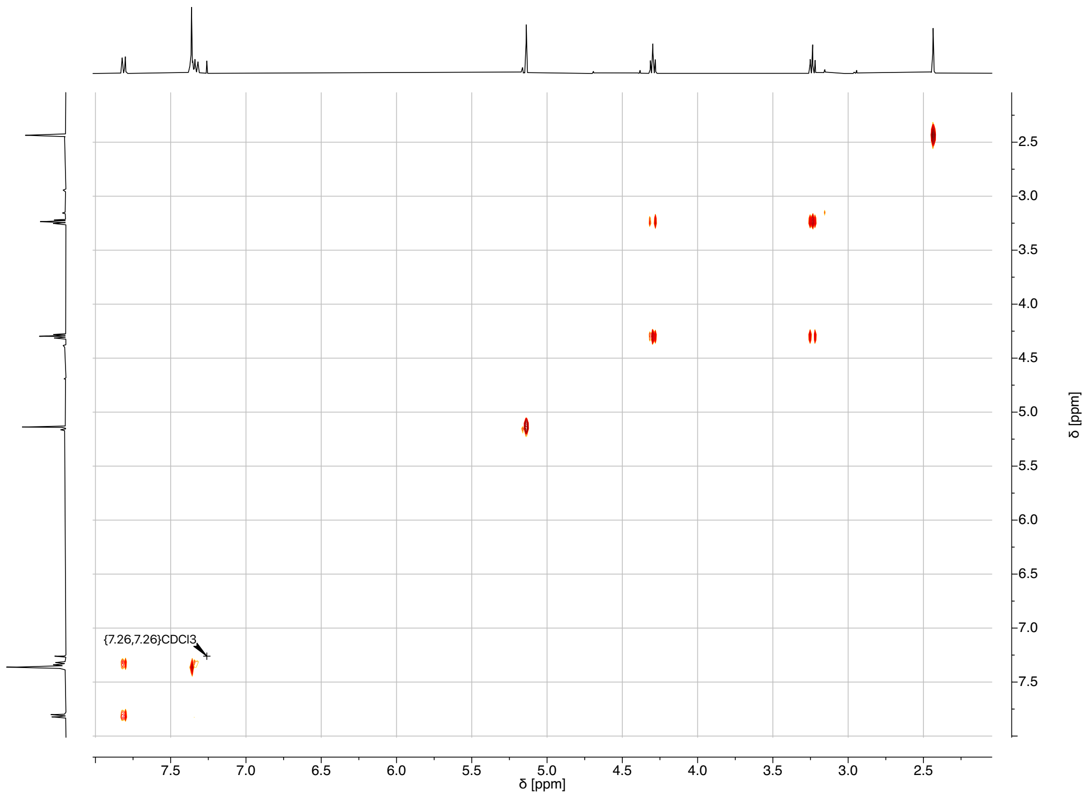

Figure S30: $^{1}H,^{1}H$ COSY spectrum of *S*-(2-(((benzyloxy)carbonyl)oxy)ethyl) 4-methylbenzenesulfonothioate.

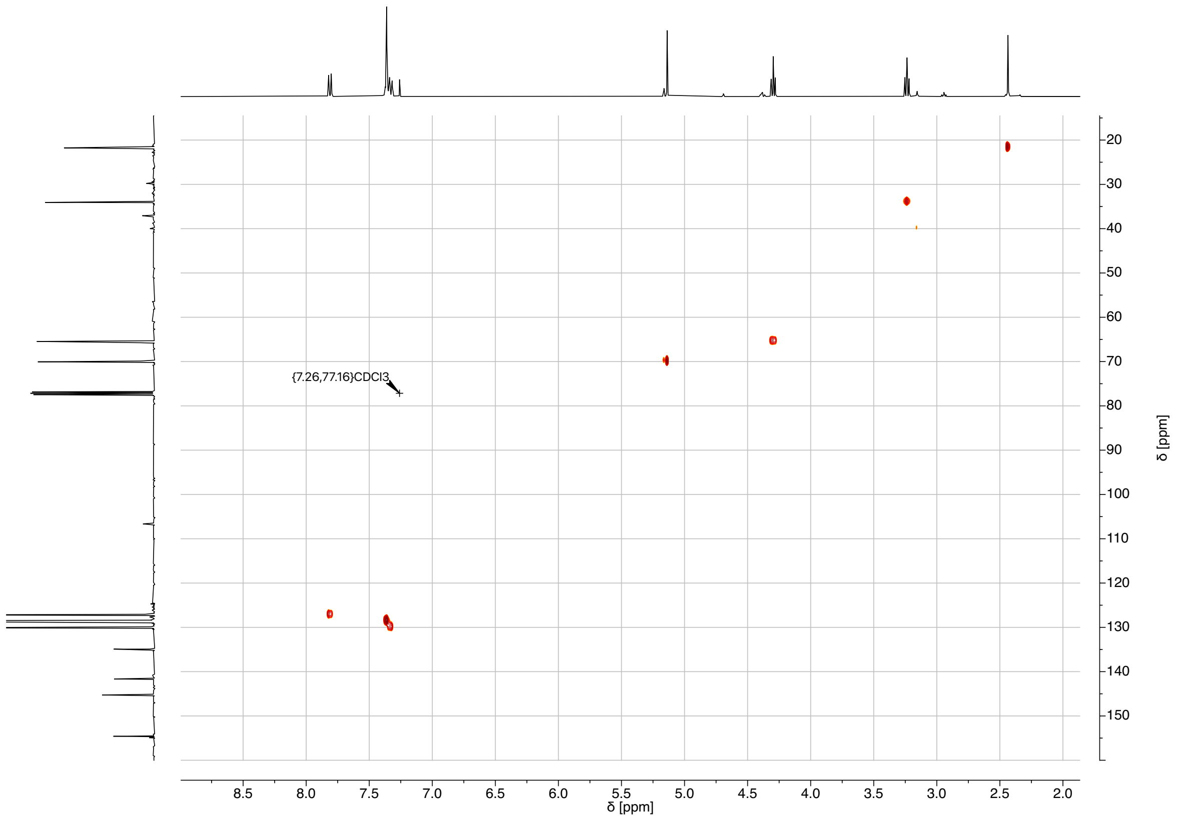

Figure S31: $^{1}H,^{13}C$ HSQC spectrum of *S*-(2-(((benzyloxy)carbonyl)oxy)ethyl) 4-methylbenzenesulfonothioate.

S-(2-((benzylcarbamoyl)oxy)ethyl) 4-methylbenzenesulfonothioate

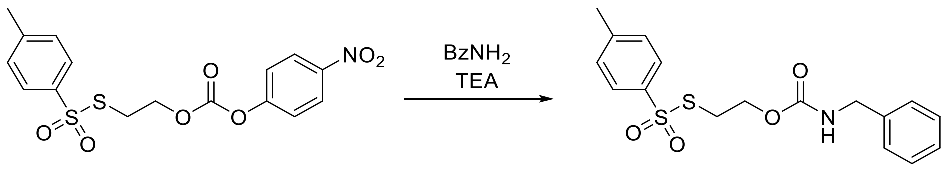

S-(2-(((4-nitrophenoxy)carbonyl)oxy)ethyl) 4-methylbenzenesulfonothioate (150 mg, 377 µmol), benzylamine (52 µL, 472 µmol) and triethylamine (79 µL, 566 µmol) were dissolved in 3 mL dry DCM and were stirred at room temperature overnight. 4-Nitrophenol was separated with basic aluminium oxide and the product (74 mg, 54%) was isolated by subsequent column chromatography (cyclohexane/ethyl acetate = 4/1).

R_f=0.25 (cyclohexane/ethyl acetate = 3/1).

^{1}H NMR (500 MHz, $CDCl_3$): δ [ppm] = 7.82 (d, J = 8.0 Hz, 2H), 7.39 – 7.27 (m, 7H), 5.14 (t, J = 6.1 Hz, 1H), 4.33 (d, J = 6.1 Hz, 2H), 4.24 (t, J = 6.4 Hz, 2H), 3.24 (t, J = 6.4 Hz, 2H), 2.44 (s, 3H).

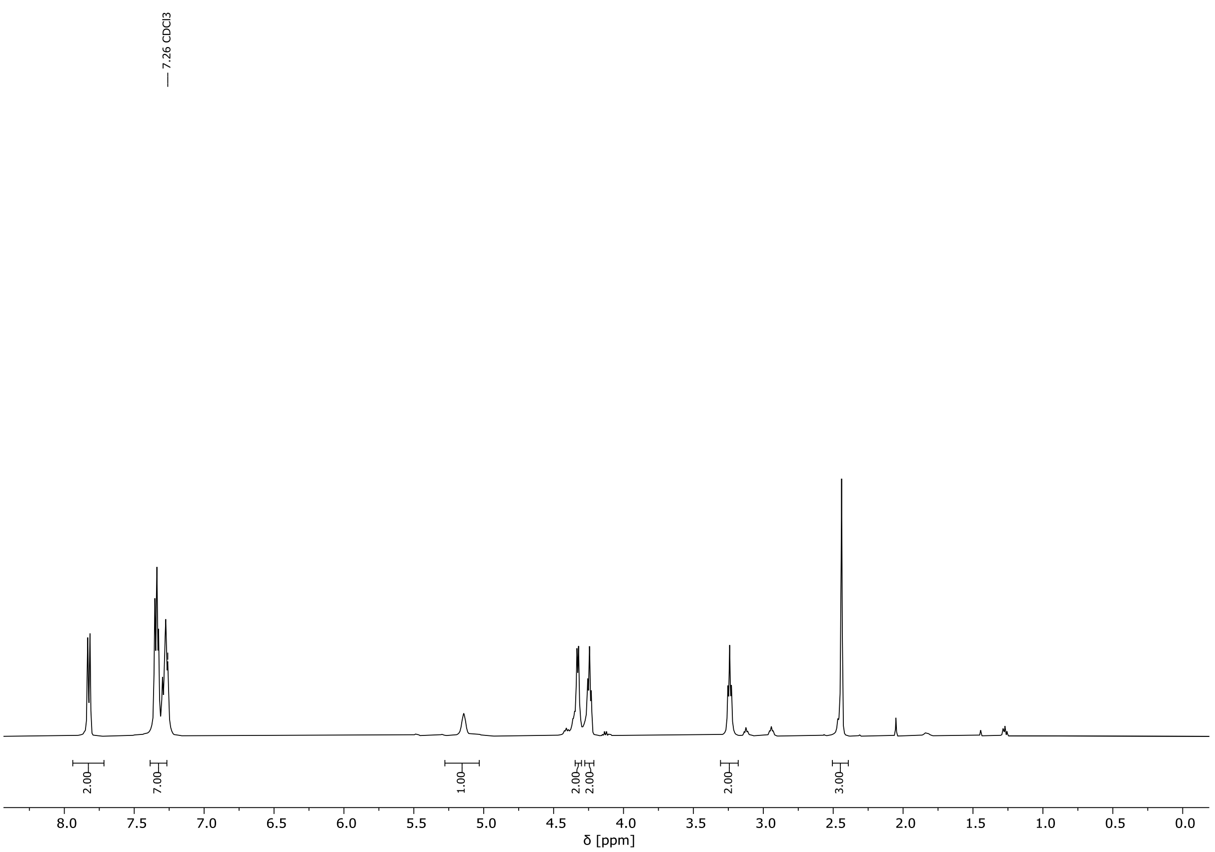

Figure S32: ^{1}H NMR (500 MHz, $CDCl_3$) of *S*-(2-((benzylcarbamoyl)oxy)ethyl) 4-methylbenzenesulfonothioate.

S-(2-((dibenzylcarbamoyl)oxy)ethyl) 4-methylbenzenesulfonothioate

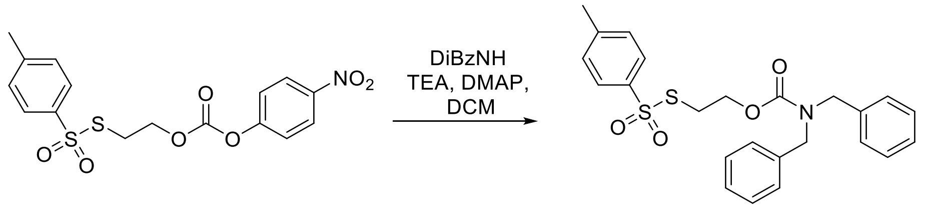

S-(2-(((4-nitrophenoxy)carbonyl)oxy)ethyl) 4-methylbenzenesulfonothioate (100 mg, 252 µmol), dibenzylamine (72.6 µL, 377 µmol), 4-dimethylaminopyridine (3.1 mg, 25 µmol) and triethylamine (52.6 µL, 377 µmol) were dissolved in 3 mL dry DCM and refluxed for three days. The solution was diluted with additional DCM and extracted three times with 1 M HCl, three times with saturated $NaHCO_3$ and three times with water. The organic layer was dried with Na_2SO_4 and the solvent was removed under reduced pressure to give the product as a slightly yellow oil (95.3 mg, 83%).

R_f=0.58 (cyclohexane/ethyl acetate = 2/1).

^{1}H NMR (400 MHz, $CDCl_3$): δ [ppm] = 7.99 – 7.80 (m, 2H), 7.45 – 7.16 (m, 12H), 4.61 – 4.34 (m, 6H), 3.42 – 3.26 (m, 2H), 2.52 (s, 3H).

^{13}C NMR (100 MHz, $CDCl_3$): δ [ppm] = 156.08, 145.12, 141.85, 137.09, 130.06, 128.74, 128.19, 127.60, 127.13, 63.13, 49.69 and 49.13, 35.03, 21.76.

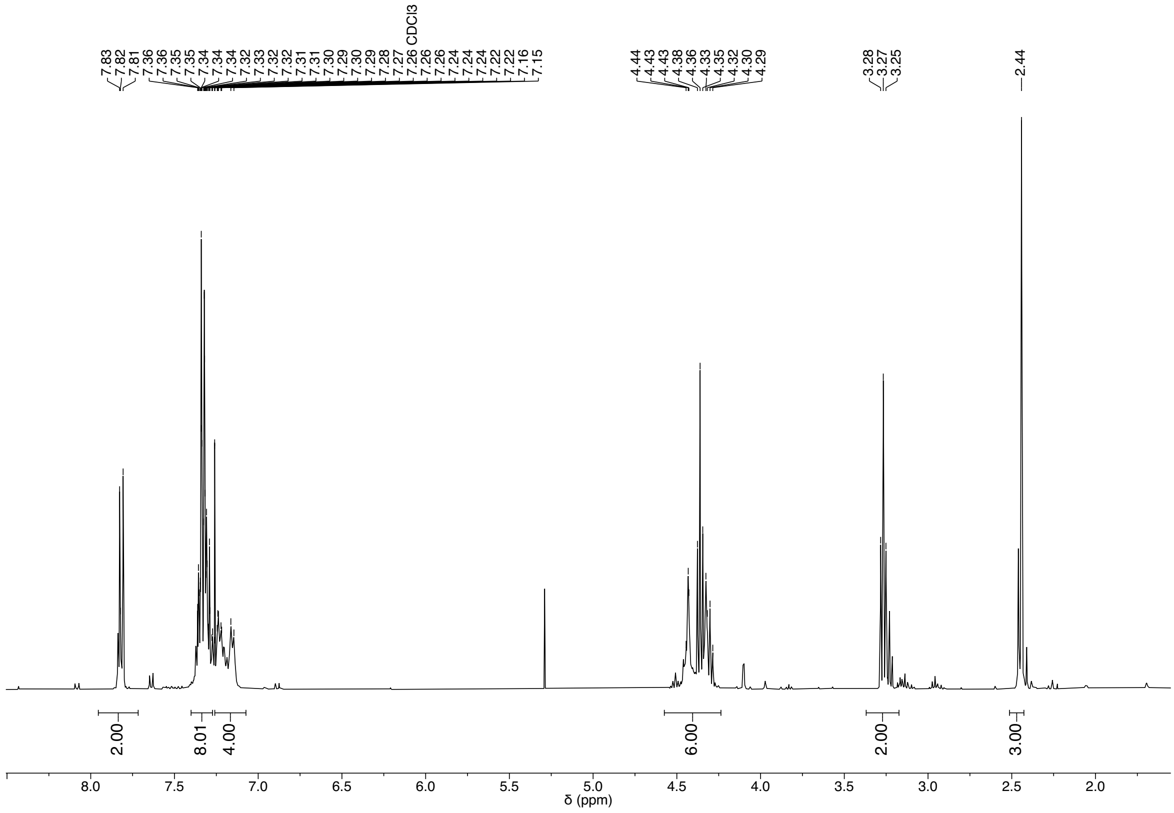

Figure S33: ^{1}H NMR (400 MHz, $CDCl_3$) of *S*-(2-((dibenzylcarbamoyl)oxy)ethyl) 4-methylbenzenesulfonothioate.

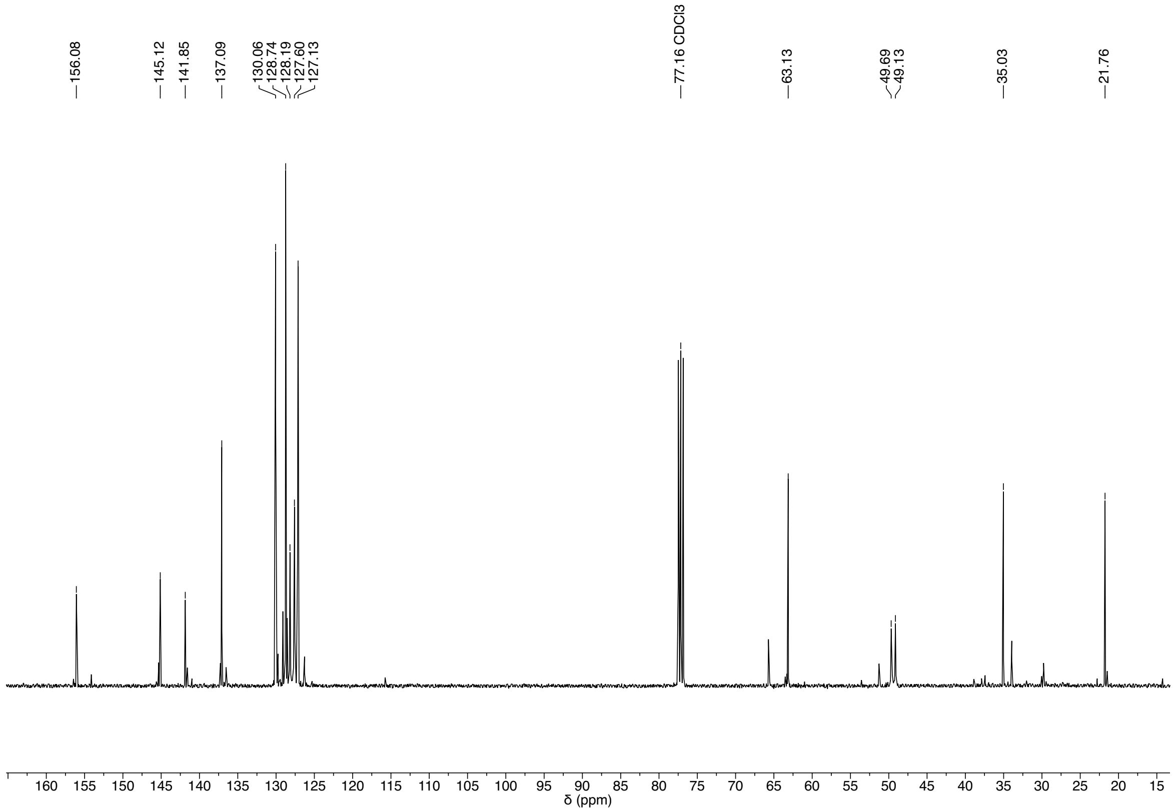

Figure S34: ^{13}C NMR (100 MHz, $CDCl_3$) of *S*-(2-((dibenzylcarbamoyl)oxy)ethyl) 4-methylbenzenesulfonothioate.

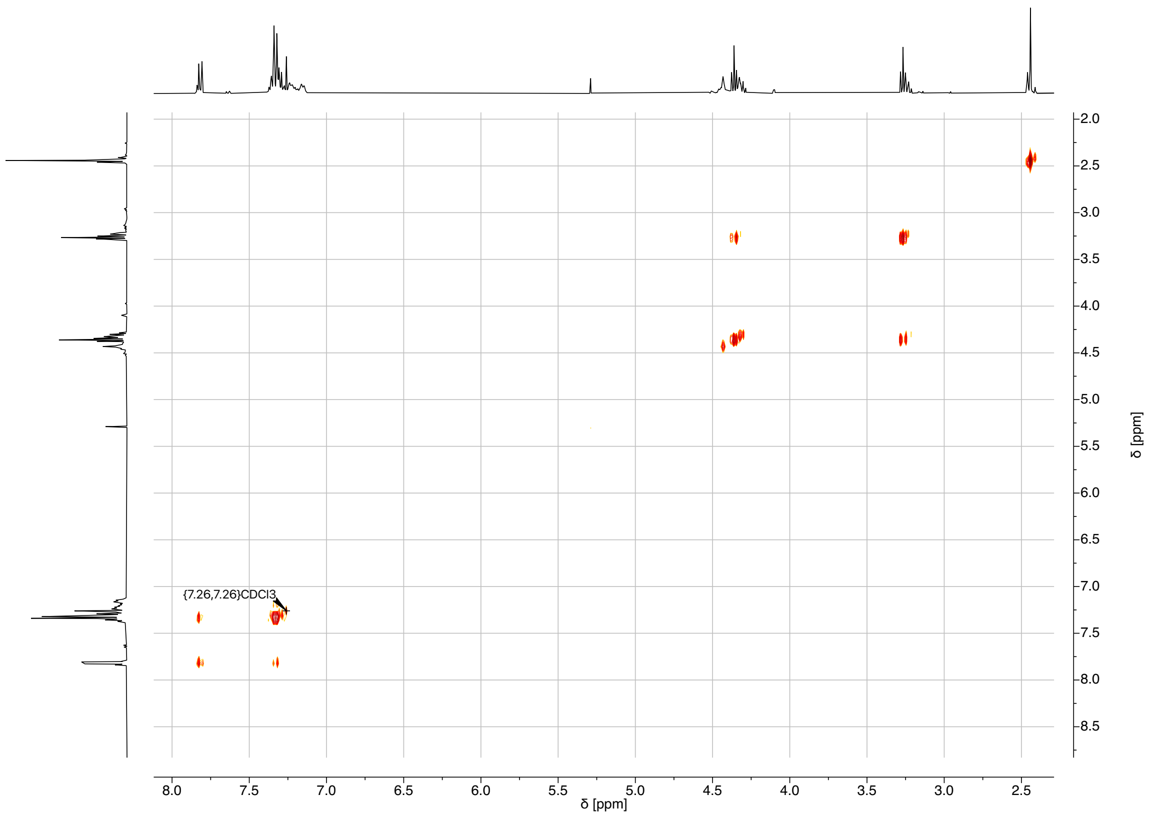

Figure S35: $^{1}H,^{1}H$ COSY spectrum of *S*-(2-((dibenzylcarbamoyl)oxy)ethyl) 4-methylbenzenesulfonothioate.

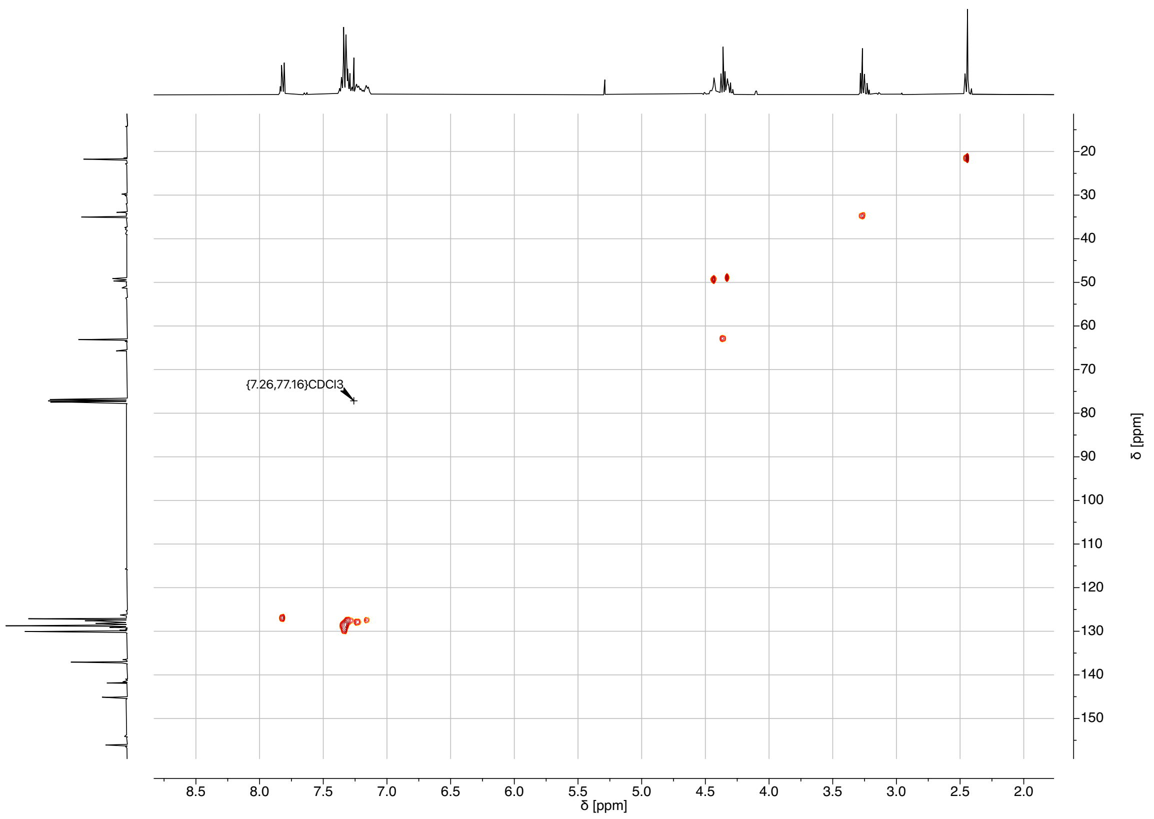

Figure S36: $^{1}H,^{13}C$ HSQC spectrum of *S*-(2-((dibenzylcarbamoyl)oxy)ethyl) 4-methylbenzenesulfonothioate.

pDMA-NHBz

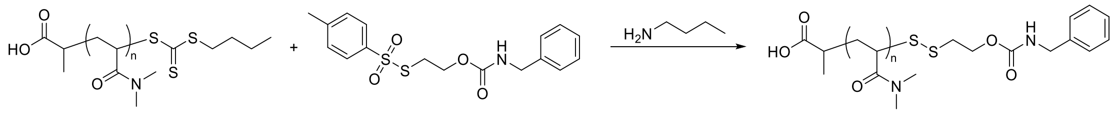

pDMA (10.6 mg, 4.4 µmol, DP 25), *n*-butylamine (2.2 µL, 21.9 µmol) and *S*-(2-((benzylcarbamoyl)oxy)ethyl) 4-methylbenzenesulfonothioate (8.0 mg, 21.9 µmol) were dissolved in 1 mL dry DMSO and stirred for 5 days at room temperature. Repeated precipitation in cold diethyl ether gave the product as a colorless powder in quantitative yields.

SEC (HFIP; PMMA standard): M_n = 4287 g mol^{-1}; M_w = 4855 g mol^{-1}; PDI = 1.13.

pDMA-OBz

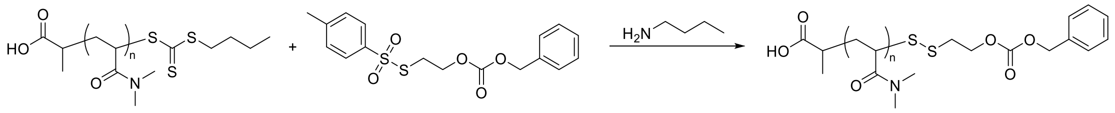

pDMA (20.0 mg, 6.2 µmol, DP 25), *n*-butylamine (3.1 µL, 31.2 µmol) and *S*-(2-(((benzyloxy)carbonyl)oxy)ethyl) 4-methylbenzenesulfonothioate (11.4 mg, 31.2 µmol) were dissolved in 0.5 mL dry DMSO and stirred for 3 days at room temperature. Repeated precipitation in cold diethyl ether gave the product as a colorless powder in quantitative yields.

SEC (HFIP; PMMA standard): M_n = 4005 g mol^{-1}; M_w = 4631 g mol^{-1}; PDI = 1.16.

pDMA-NDiBz

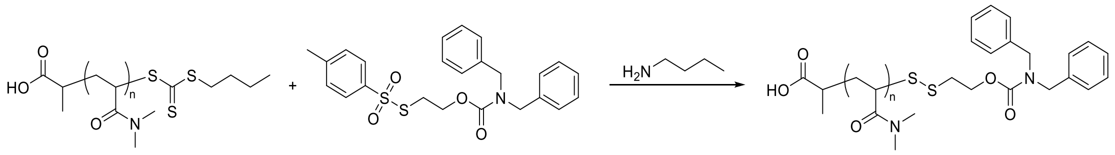

pDMA (20.0 mg, 6.2 µmol, DP 25), , *n*-butylamine (3.1 µL, 31.2 µmol) and *S*-(2-((dibenzylcarbamoyl)oxy)ethyl) 4-methylbenzenesulfonothioate (14.2 mg, 31.2 µmol) were dissolved in 0.5 mL dry DMSO and stirred for 3 days at room temperature. Repeated precipitation in cold diethyl ether gave the product as a colorless powder in quantitative yields.

SEC (HFIP; PMMA standard): M_n = 4138 g mol^{-1}; M_w = 4845 g mol^{-1}; PDI = 1.17.

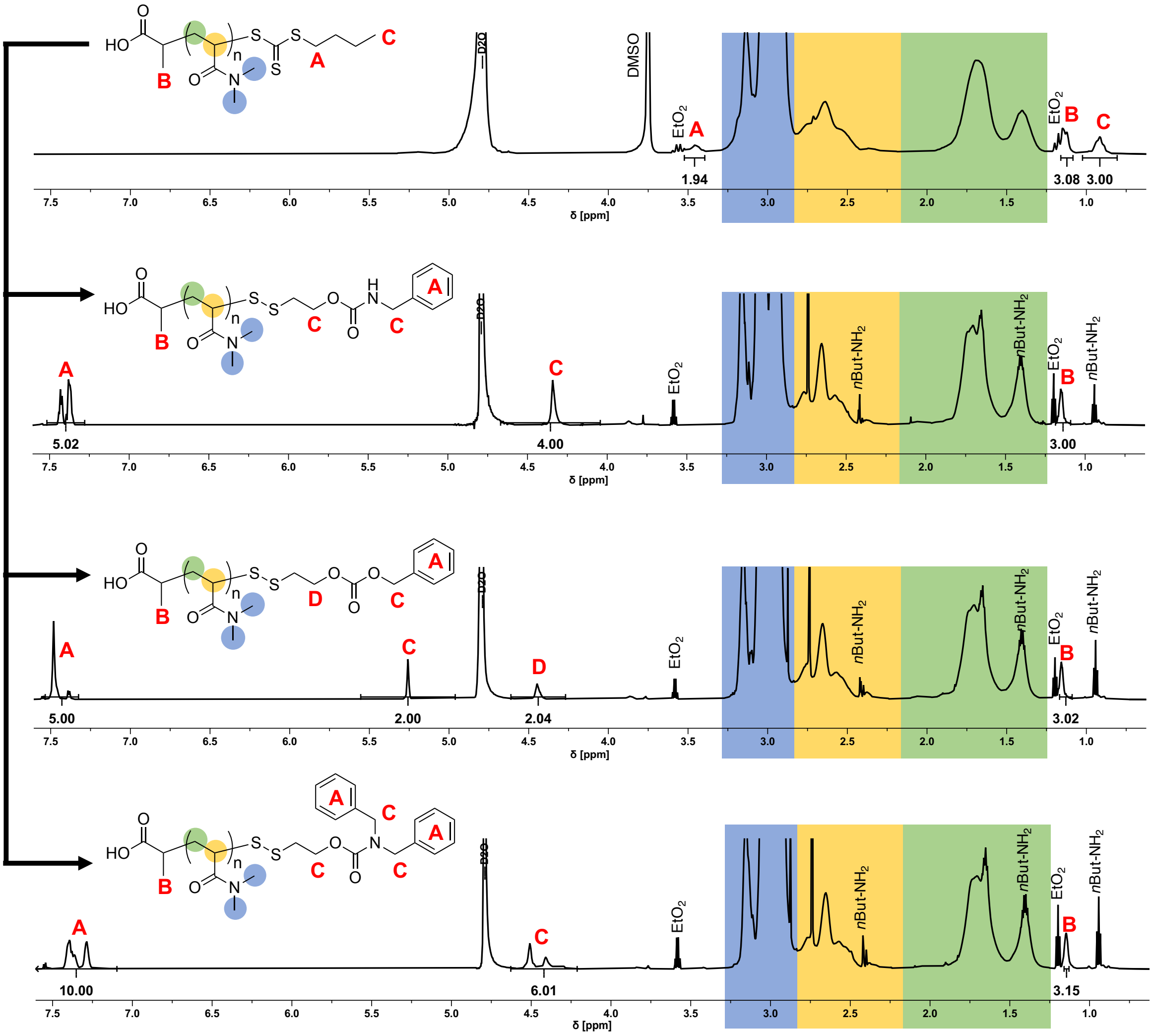

Figure S37: ^{1}H NMR spectra for the end group analyses of RAFT-derived pDMA before and after self-immolative end group modification affording pDMA-NHBz, pDMA-OBz and pDMA-NDiBz.

1-(4-(aminomethyl)benzyl)-2-butyl-1*H*-imidazo[4,5-c]quinolin-4-amine (IMDQ)

IMDQ was synthesized as previously described.[3]

***S*-(2-(((4-((4-amino-2-butyl-1*H*-imidazo[4,5-*c*]quinolin-1-yl)methyl)benzyl)carbamoyl)-oxy)ethyl) 4-methylbenzenesulfonothioate (SIL-IMDQ)**

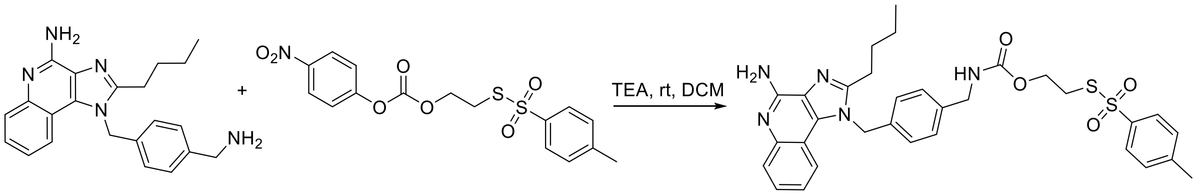

IMDQ (50.0 mg, 139 µmol), *S*-(2-(((4-nitrophenoxy)carbonyl)oxy)ethyl) 4-methylbenzene-sulfonothioate (66.3 mg, 167 µmol) and triethylamine (29.1 µL, 209 µmol) were dissolved in 1.5 mL dry DCM and stirred at room temperature for 20 h. The product (60.0 mg, 70%) was isolated by column chromatography (ethyl acetate/methanol = 8/1).

R_f=0.09 (ethyl acetate).

^{1}H NMR (700 MHz, $CDCl_3$): δ [ppm] = 7.80 – 7.71 (m, 3H), 7.63 (d, 1H), 7.37 (t, 1H), 7.28 (d, *J* = 7.9 Hz, 2H), 7.18 (d, *J* = 8.0 Hz, 2H), 7.07 (t, 1H), 6.97 (d, *J* = 7.9 Hz, 2H), 5.86 (s, 2H), 5.64 (s, 2H), 5.28 (s, 1H), 4.26 (d, *J* = 6.0 Hz, 2H), 4.19 (t, *J* = 6.3 Hz, 2H), 3.18 (t, *J* = 6.4 Hz, 2H), 2.81 (t, *J* = 7.9 Hz, 2H), 2.38 (s, 3H), 1.76 (quint, *J* = 7.6 Hz, 2H), 1.40 (sext, *J* = 7.4 Hz, 2H), 0.89 (t, *J* = 7.4 Hz, 3H).

^{13}C NMR (175 MHz, $CDCl_3$): δ [ppm] = 155.87, 154.21, 151.14, 145.12, 143.96, 141.82, 138.38, 134.70, 134.05, 130.02, 128.38, 127.24, 127.12, 126.67, 126.39, 125.93, 122.44, 119.81, 115.04, 62.58, 48.64, 44.56, 34.96, 29.98, 27.17, 22.57, 21.70, 13.84.

ESI-MS (m/z): $[M+H]^+$ 618.22, found 618.20.

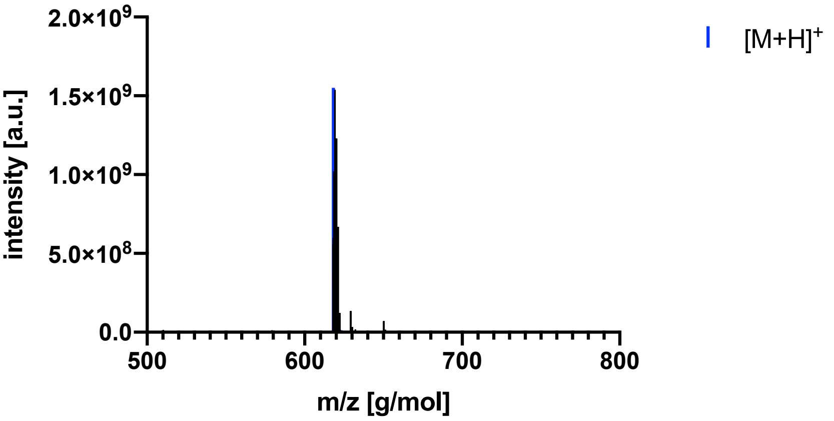

Figure S38: ESI-MS of SIL-IMDQ.

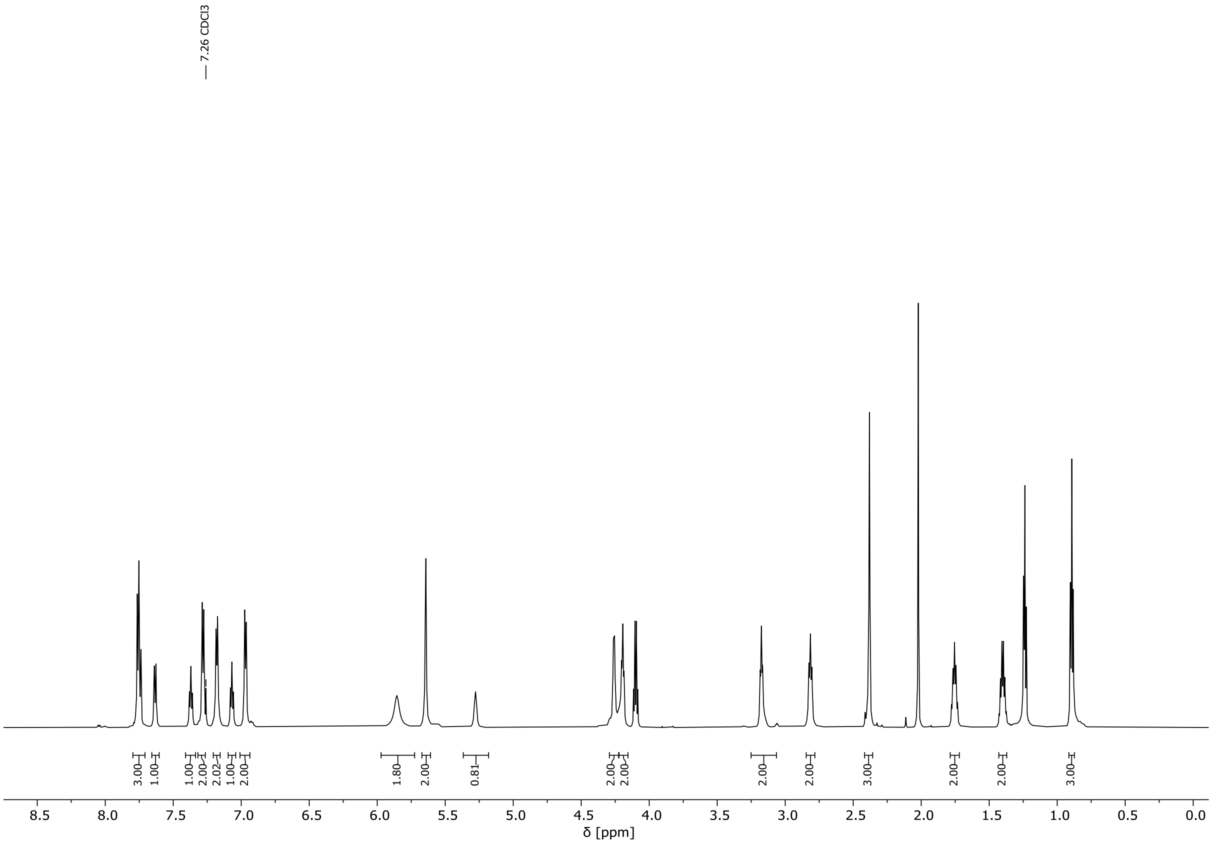

Figure S39: ^{1}H NMR (700 MHz, $CDCl_3$) of SIL-IMDQ.

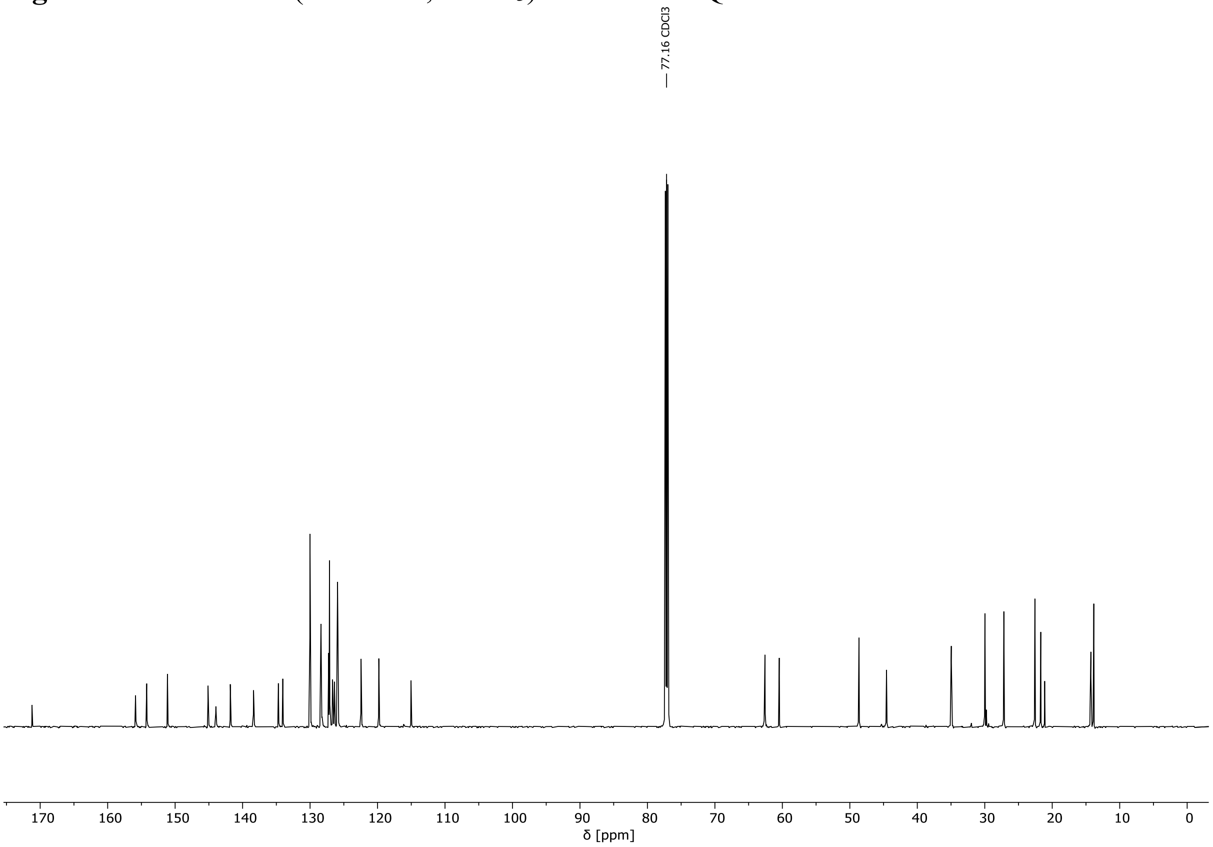

Figure S40: ^{13}C NMR (175 MHz, $CDCl_3$) of SIL-IMDQ.

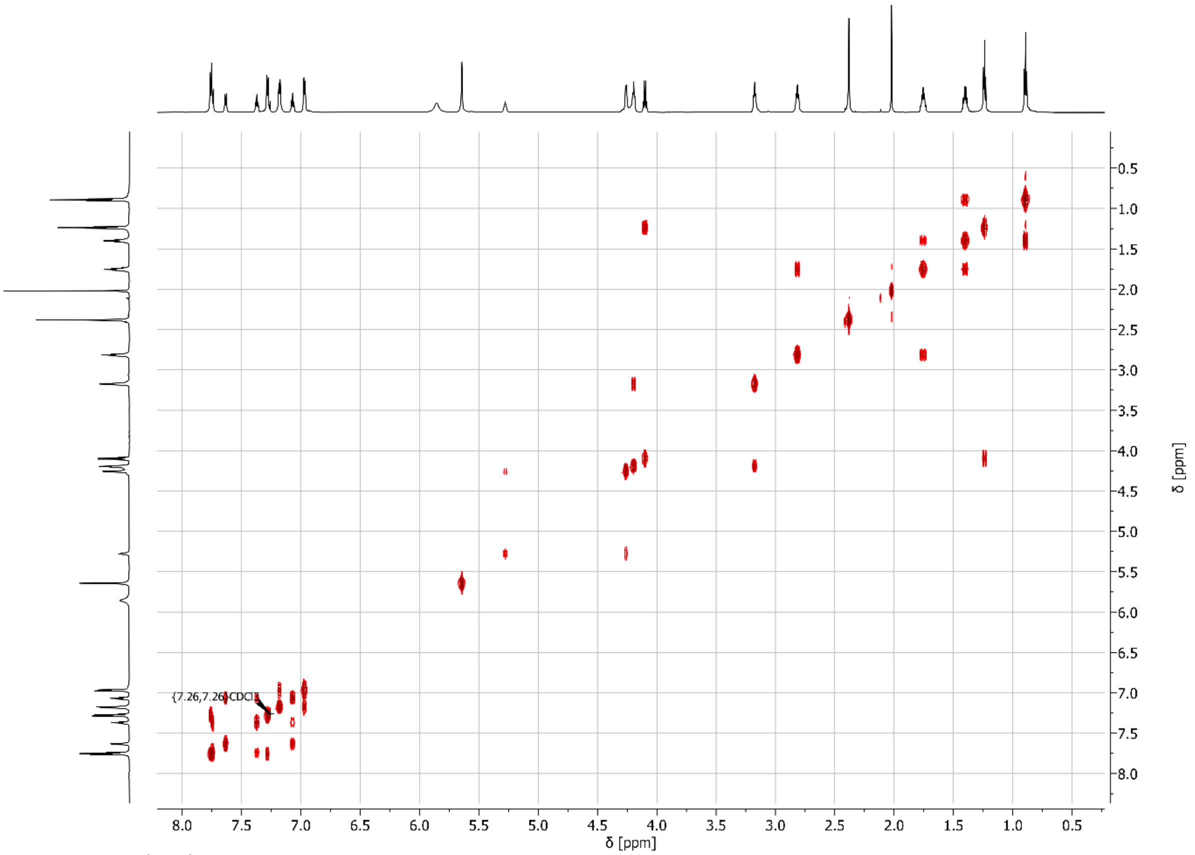

Figure S41: $^{1}H,^{1}H$ COSY spectrum of SIL-IMDQ.

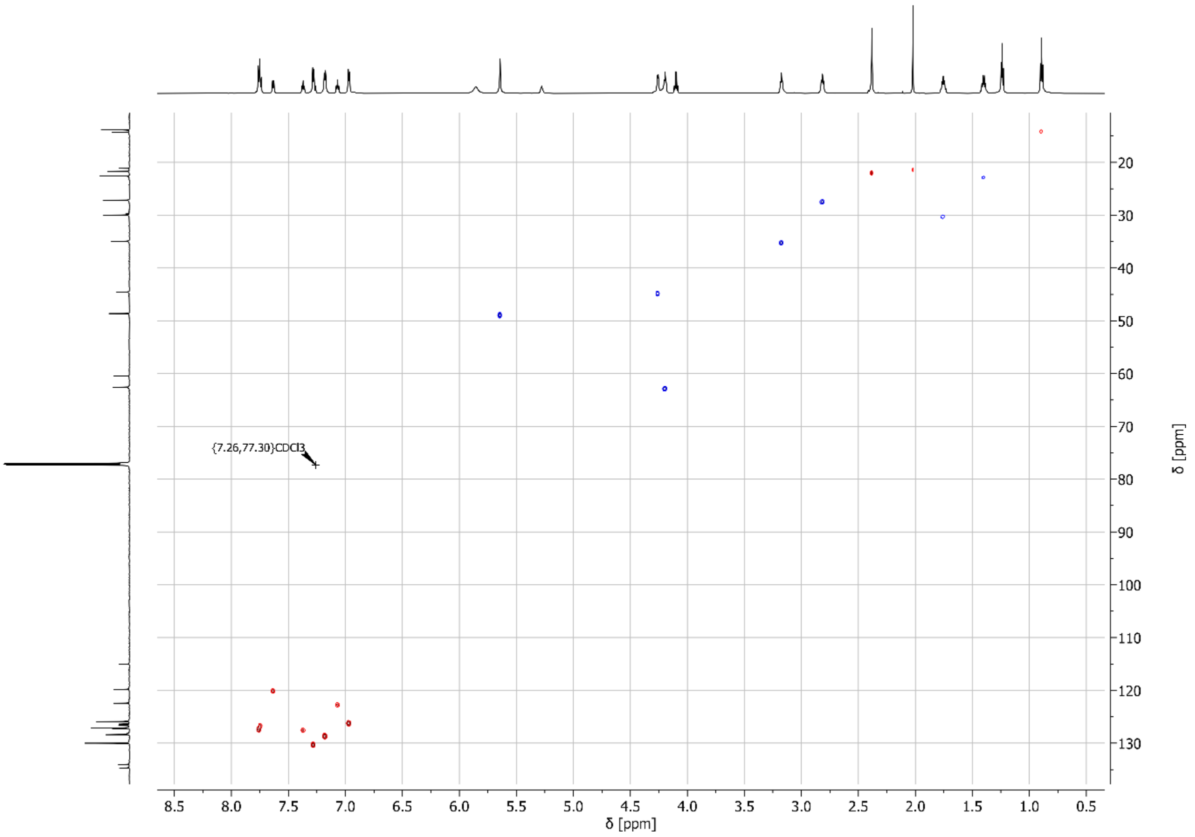

Figure S42: $^{1}H,^{13}C$ HSQC spectrum of SIL-IMDQ.

N-(4-((4-amino-2-butyl-1H-imidazo[4,5-c]quinolin-1-yl)methyl)benzyl)-4-((2,5-dioxo-2,5-dihydro-1H-pyrrol-1-yl)methyl)cyclohexane-1-carboxamide (mal-IMDQ)

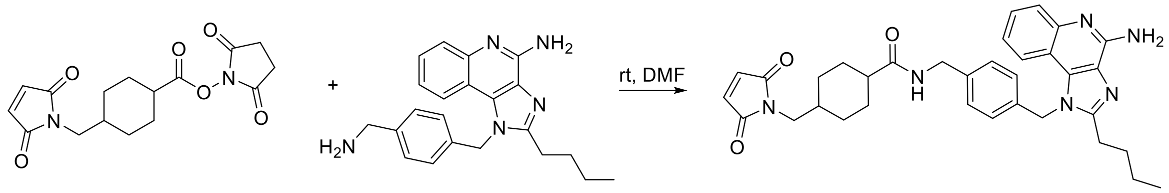

SMCC (50.0 mg, 150 µmol) and IMDQ (51.2 mg, 142 µmol) were dissolved in 2.5 mL dry DMF and stirred at room temperature for 3 h. The solvent was removed and column chromatography (chloroform/methanol = 20/1 – 10/1/0.05 NH_3) gave the product as a faint beige solid (69 mg, 84%).

R_f=0.23 (chloroform/methanol/ammonia = 10/1/0.05).

^{1}H NMR (300 MHz, $CDCl_3$): δ [ppm] = 7.78 (d, J = 8.4 Hz, 1H), 7.66 (d, J = 8.2 Hz, 1H), 7.41 (t, J = 7.7 Hz, 1H), 7.18 (d, J = 7.8 Hz, 2H), 7.10 (t, J = 7.6 Hz, 1H), 6.97 (d, J = 7.8 Hz, 2H), 6.67 (s, 2H), 5.94 – 5.79 (m, 2H), 5.68 (s, 2H), 4.36 (d, J = 5.8 Hz, 2H), 3.32 (d, J = 6.8 Hz, 2H), 2.84 (t, J = 7.8 Hz, 2H), 2.06 – 1.94 (m, 1H), 1.94 – 1.06 (m, 13H), 0.91 (t, J = 7.3 Hz, 3H).

^{13}C NMR (75 MHz, $CDCl_3$): δ [ppm] = 175.57, 171.10, 138.75, 134.09, 128.60, 125.95, 119.91, 51.38, 48.74, 45.24, 43.72, 42.88, 36.41, 30.01, 29.88, 28.95, 27.23, 22.61, 13.88.

ESI-MS (m/z): $[M+H]^+$ 579.31, found 579.32.

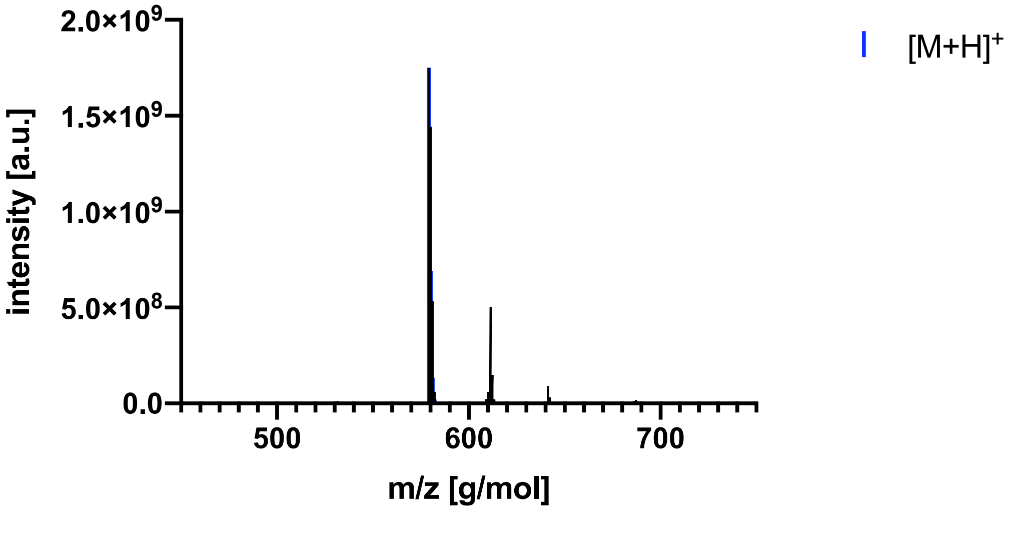

Figure S43: ESI-MS of mal-IMDQ.

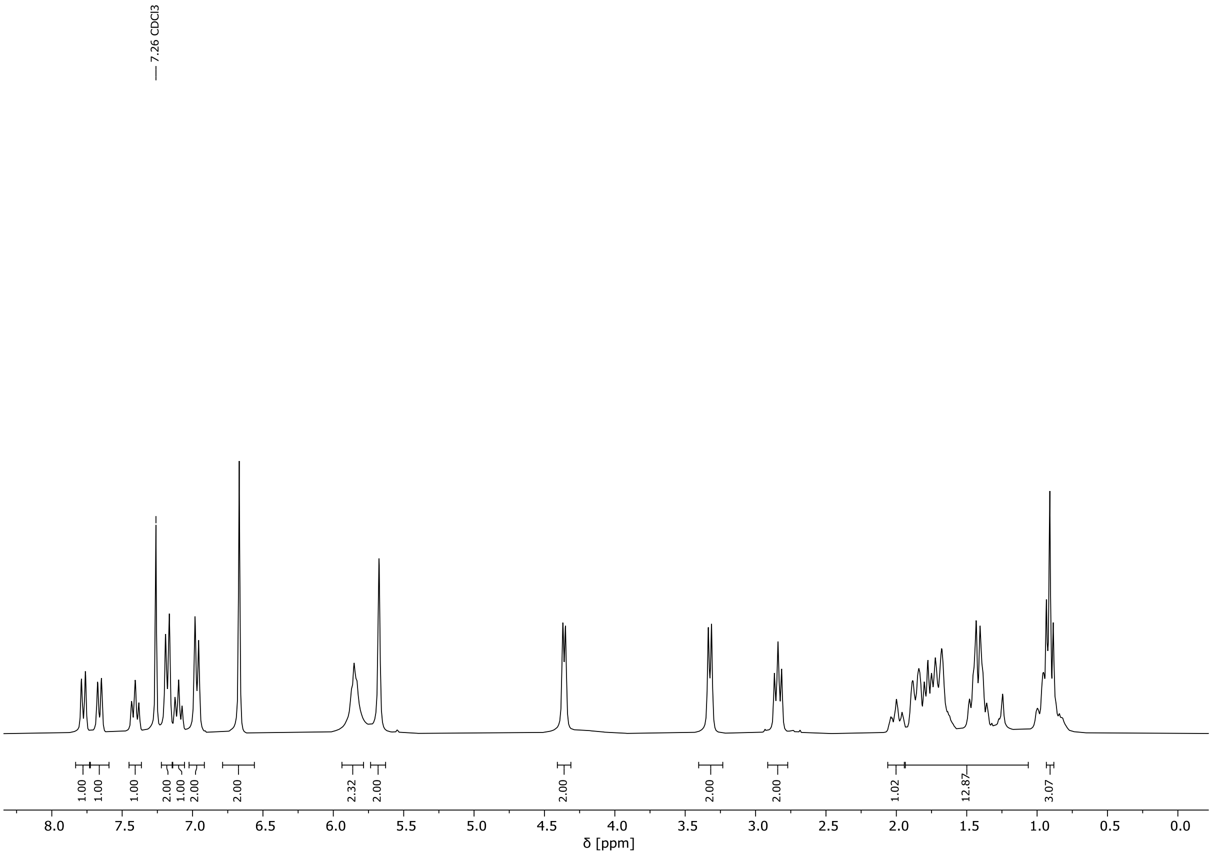

Figure S44: ^{1}H NMR (300 MHz, $CDCl_3$) of mal-IMDQ.

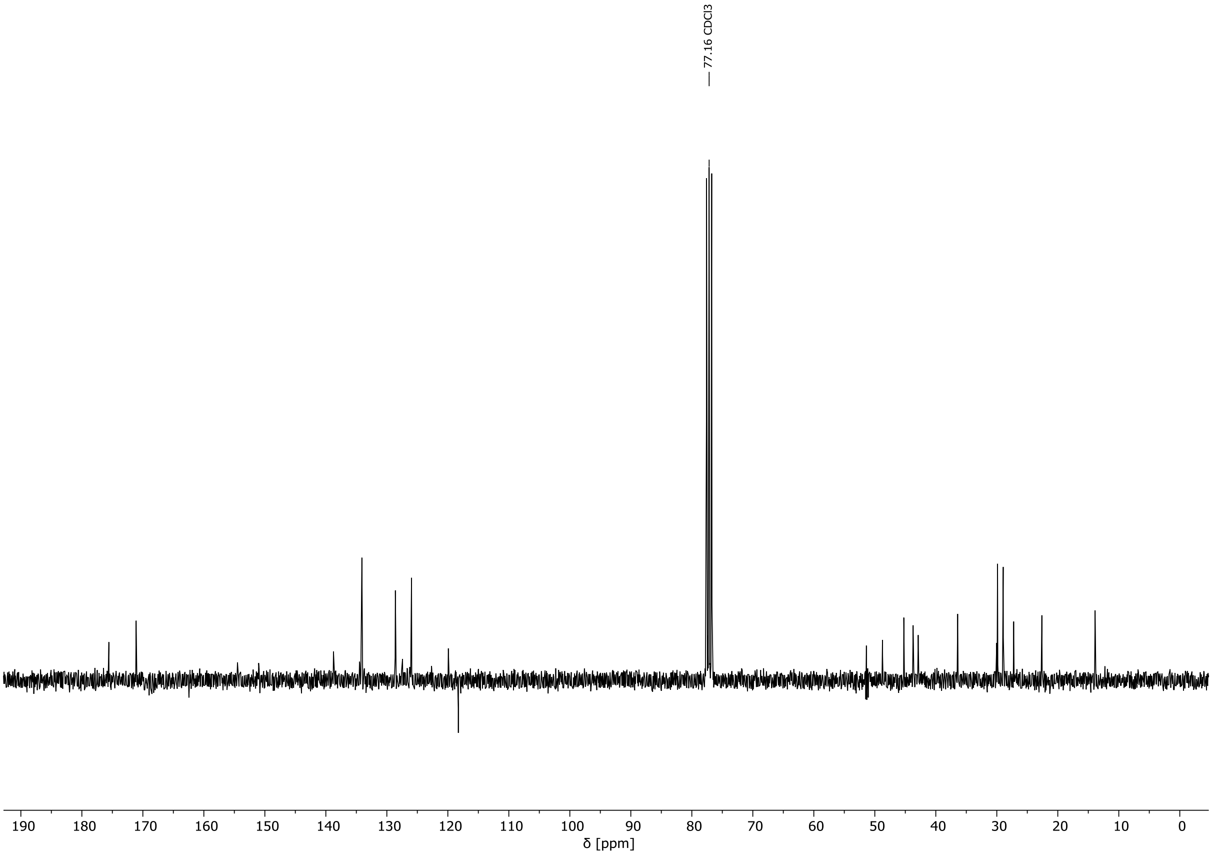

Figure S45: ^{13}C NMR (75 MHz, $CDCl_3$) of mal-IMDQ.

2-(6-(dimethylamino)-3-(dimethyliminio)-3H-xanthen-9-yl)-5-((5-(((2-(tosylthio)ethoxy)-carbonyl)amino)pentyl)carbamoyl)-benzoate (TAMRA-SIL-Ts)

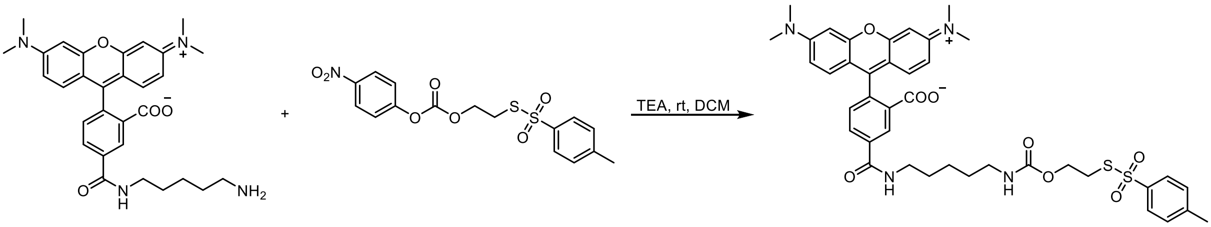

TAMRA cadaverine (5 mg, 9.72 µmol), S-(2-(((4-nitrophenoxy)carbonyl)oxy)ethyl) 4-methylbenzenesulfonothioate (7.73 mg, 19.44 µmol) and triethylamine (4.04 µL, 29.16 µmol) were dissolved in 300 µL dry DMSO and stirred at room temperature overnight. The reaction mixture was diluted with water (DMSO <10%) and lyophilized. Impurities were separated by using a column packed with basic AlOx and then the product was further purified using column chromatography (cyclohexane/ethyl acetate = 1/1 → 0/1). Pure product was eluted by dichloromethane/methanol = 5/1.

Solvents were removed, the residue was taken up in 1 mL of dry DMSO and the compounds concentration was determined using a TAMRA-SIL-Ts solution of known concentration as calibration.

MALDI-TOF-MS (m/z): $[M+H]^+$ 773.27, found 773.40.

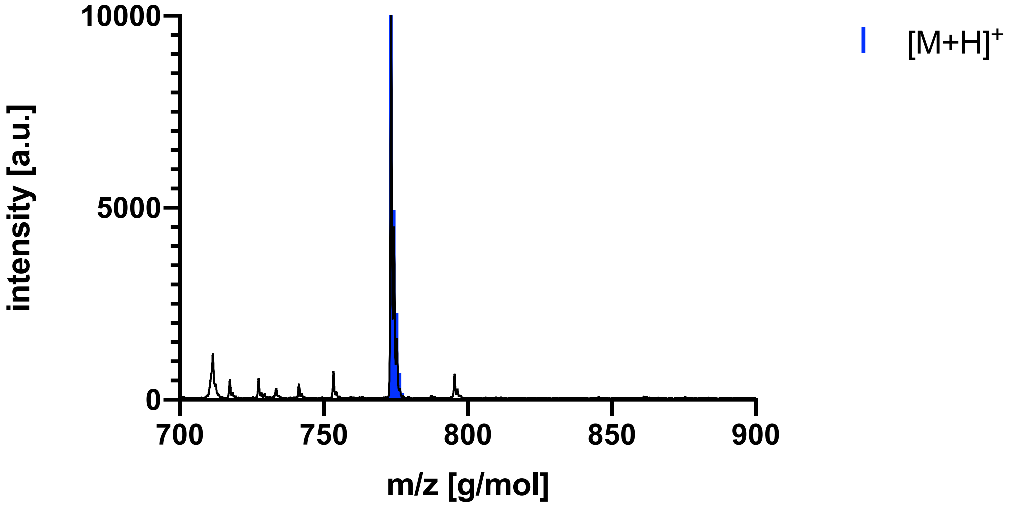

Figure S46: MALDI-TOF-MS of TAMRA-SIL-Ts.

pDMA-SIL-TAMRA

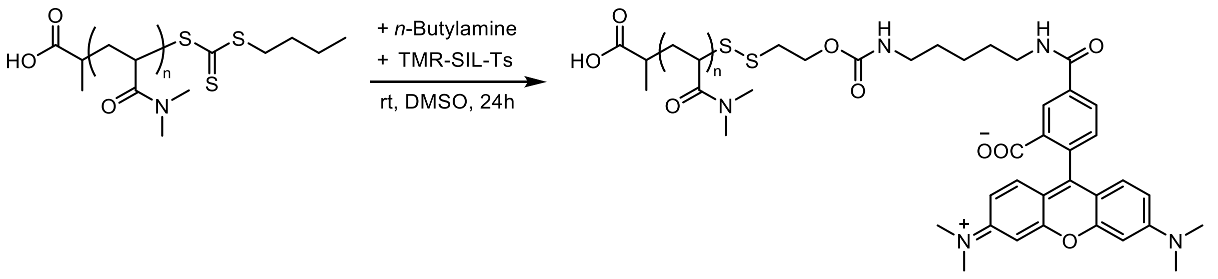

pDMA (5 mg, 0.96 µmol, DP 50) was dissolved in dry DMSO and *n*-butylamine (0.5 µL, 5 µmol) as well as TAMRA-SIL-Ts (359.5 µL of 2.58 mg/mL stock-solution, 1.2 µmol) were added. The reaction was left to stir overnight. The next day, it was diluted with MilliQ water to a volume of 5 ml, dialysed against MilliQ water for three days and lyophilized overnight affording the product as a pink powder in quantitative yields.

SEC (HFIP; PMMA standard): M_n = 8478 g mol^{-1}; M_w = 9779 g mol^{-1}; PDI = 1.15.

Cholesteryl 2-(((butylthio)carbonothioyl)thio)propanoate (Chol-PABTC)

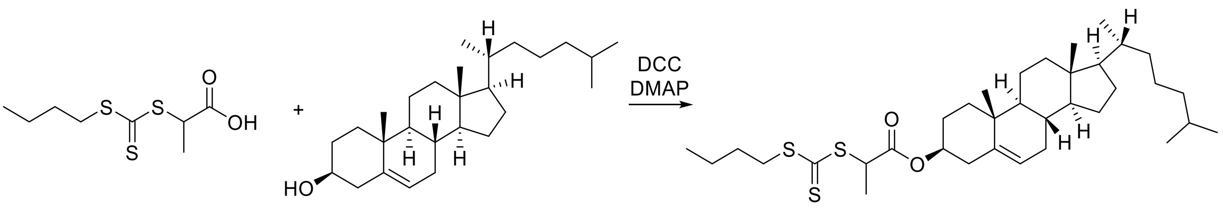

PABTC (203 mg, 0.841 mmol), 4-dimethylaminopyridine (10.8 mg, 0.088 mmol) and cholesterol (358 mg, 0.925 mmol) were dissolved in 10 mL dry DCM and cooled to 0 °C. DCC (182 mg, 0.883 mmol) in 10 mL dry DCM was added dropwise over the period of 1 h. The solution was stirred another hour at 0 °C before it was stirred at room temperature overnight. The mixture was filtered, washed with DCM and the solvent was removed under reduced pressure. Column chromatography (cyclohexane/ethyl acetate = 5/1) gave the product in quantitative yields as a yellow solid (510 mg, quant.).

R_f=0.88 (cyclohexane/ethyl acetate = 5/1).

^{1}H NMR (400 MHz, $CDCl_3$): δ [ppm] = 5.39 – 5.35 (m, 1H), 4.76 (q, J = 7.3 Hz, 1H), 4.71 – 4.55 (m, 1H), 3.36 (t, J = 7.4 Hz, 2H), 2.33 (t, J = 6.8 Hz, 2H), 2.07 – 1.92 (m, 2H), 1.91 – 1.76 (m, 3H), 1.72 – 1.60 (m, J = 7.5 Hz, 3H), 1.59 (d, 3H), 1.56 – 1.43 (m, 6H), 1.42 – 1.03 (m, 14H), 1.02 (s, 3H), 1.00 – 0.95 (m, 2H), 0.93 (t, J = 7.4 H, 3H), 0.91 (d, J = 6.3 Hz 3H), 0.86 (d, J = 6.6 Hz, 6H), 0.67 (s, 3H).

^{13}C NMR (100 MHz, $CDCl_3$): δ [ppm] = 222.23, 170.64, 139.52, 123.02, 75.68, 56.80, 56.25, 50.1, 48.49, 42.43, 39.84, 39.65, 37.97, 37.03, 36.71, 36.31, 35.93, 32.04, 31.96, 30.09, 28.37, 28.15, 27.69, 27.05, 24.42, 23.96, 22.97, 22.71, 22.20, 21.16, 19.46, 18.85, 17.06, 13.75, 11.99.

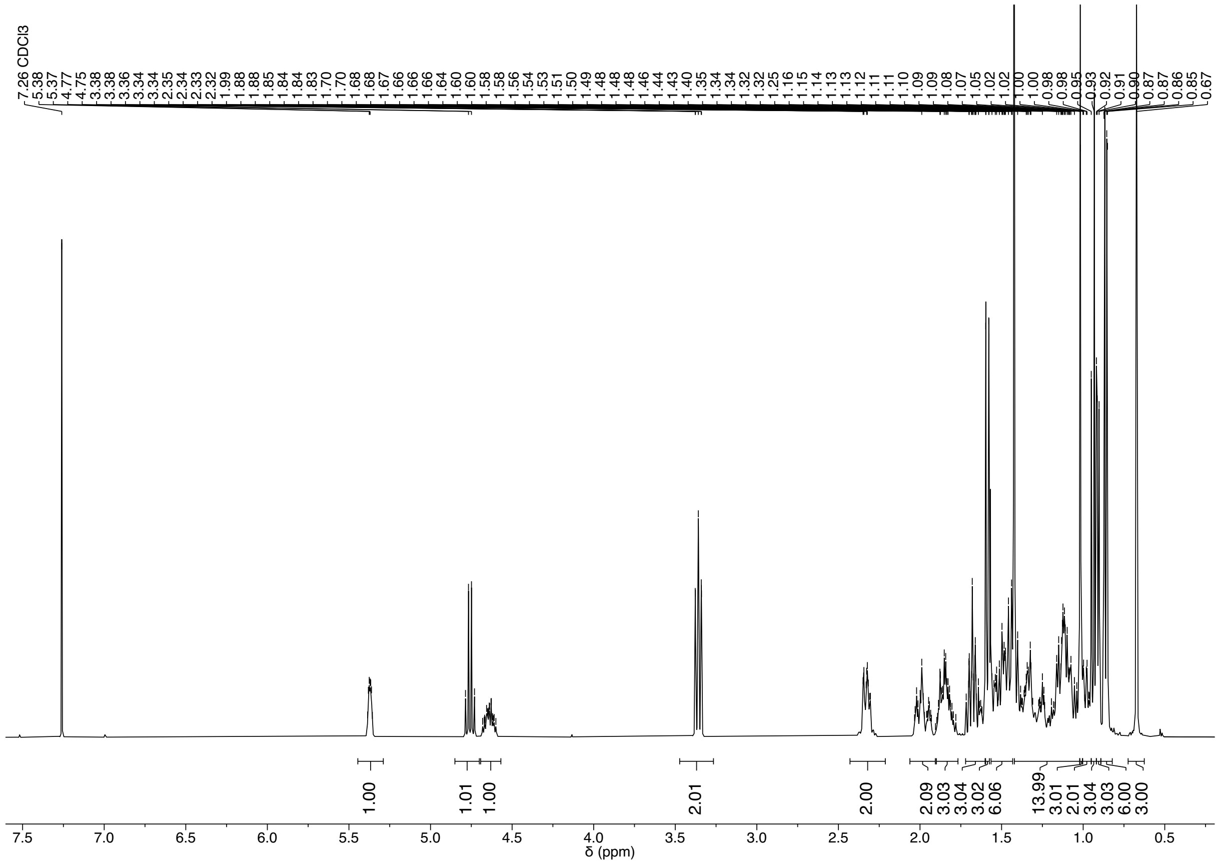

Figure S47: ^{1}H NMR (400 MHz, $CDCl_3$) of cholesteryl 2-(((butylthio)carbonothioyl)thio)propanoate.

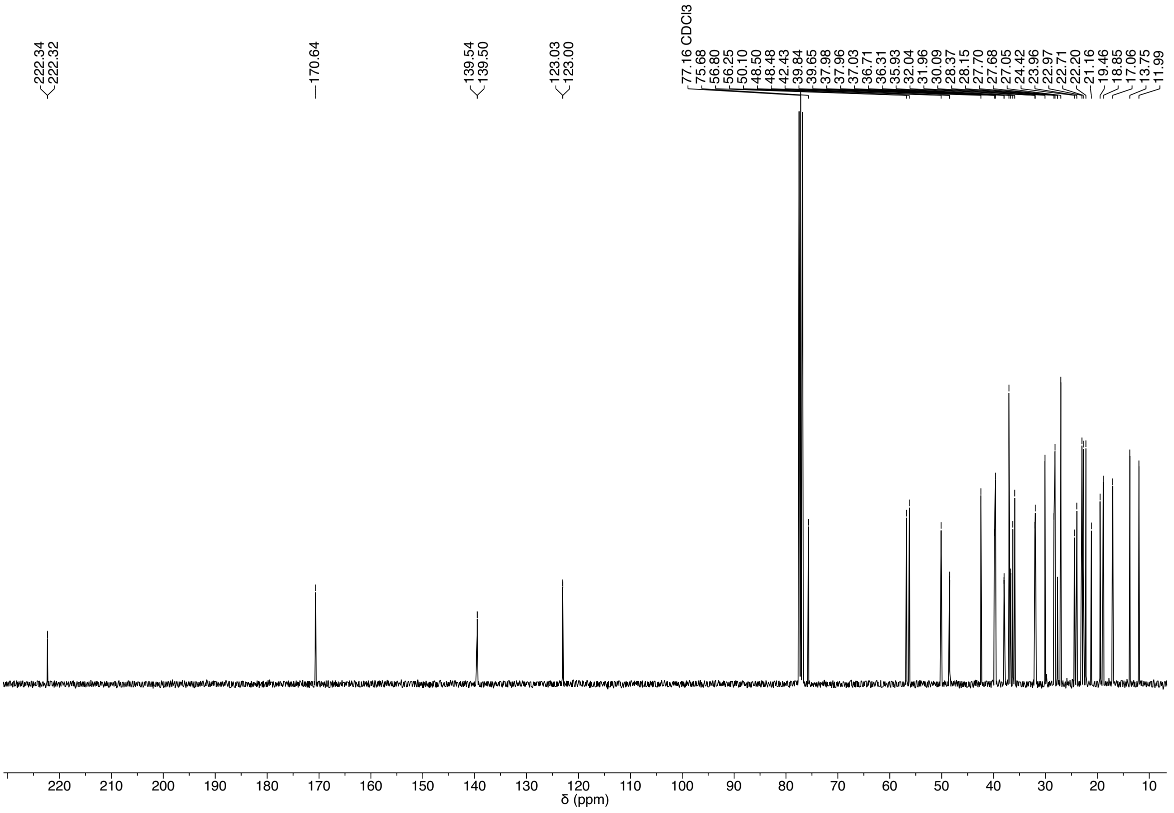

Figure S48: ^{13}C NMR (100 MHz, $CDCl_3$) of cholesteryl 2-(((butylthio)carbonothioyl)thio)propanoate.

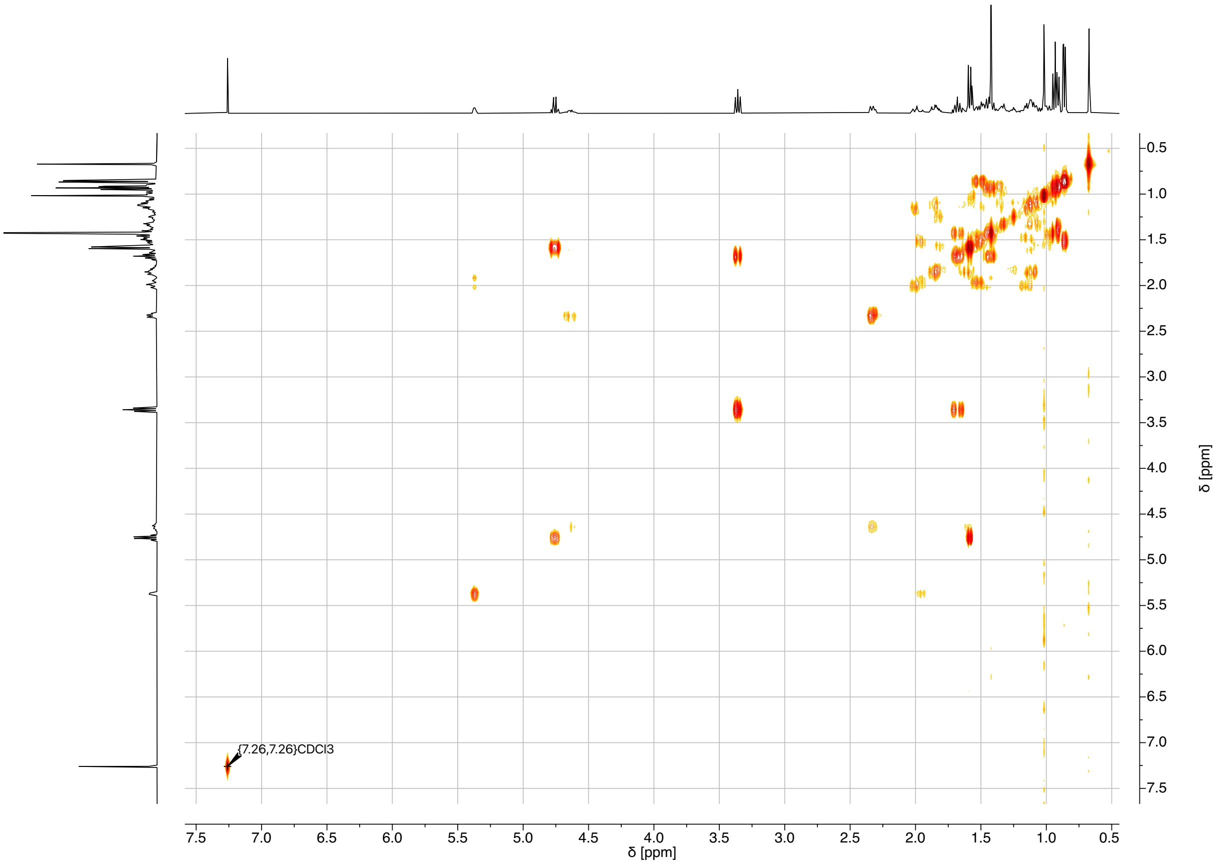

Figure S49: $^{1}H,^{1}H$ COSY spectrum of cholesteryl 2-(((butylthio)carbonothioyl)thio)propanoate.

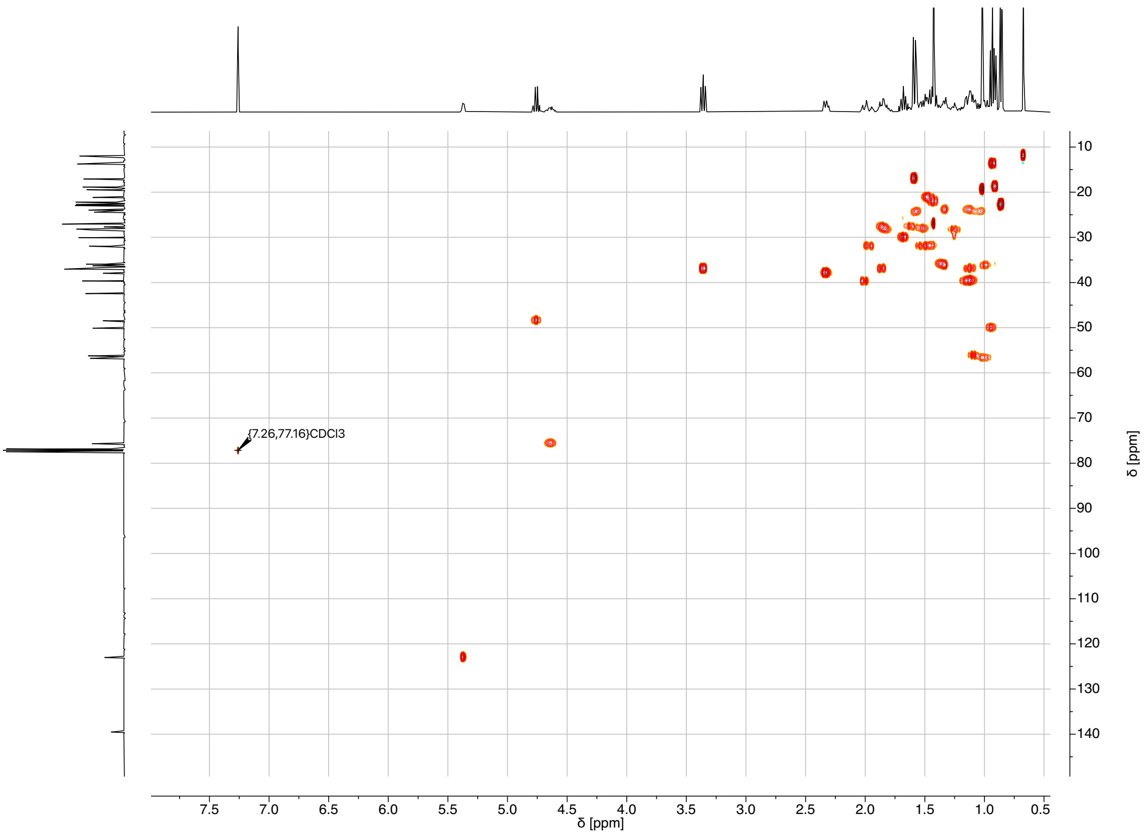

Figure S50: $^{1}H,^{13}C$ HSQC spectrum of cholesteryl 2-(((butylthio)carbonothioyl)thio)propanoate.

Polymerization of *N,N*-dimethylacrylamide with (Chol-PABTC)

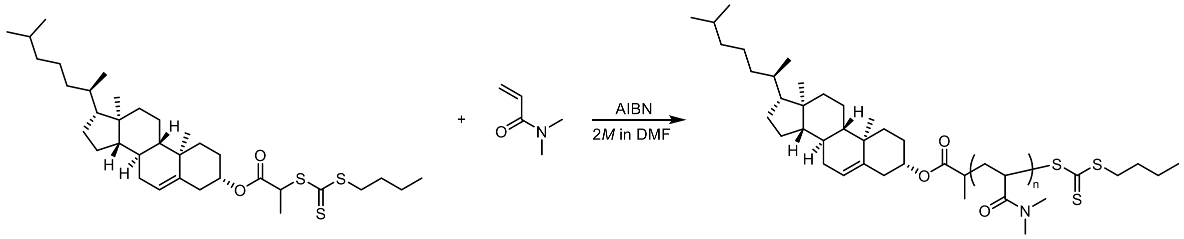

For a degree of polymerization of 50 repeating units, cholesteryl 2-(((butylthio)carbonothioyl)thio)propanoate (21.4 mg, 35.25 µmol), *N,N*-dimethylacrylamide (181.2 µl, 1762.5 µmol) and AIBN (1 mg, 6.09 µmol) were dissolved in a 1 mL mixture of 1:1 dioxane and DMF. After 4 freeze-pump-thaw cycles, the reaction mixture was suspended into a preheated oil bath and stirred at 75°C for 5 hours. The product was precipitated in cold diethyl ether repeatedly and lyophilized over the weekend.

SEC (HFIP; PMMA standard): M_n = 10,572 g mol^{-1}; M_w = 11,976 g mol^{-1}; PDI = 1.13.

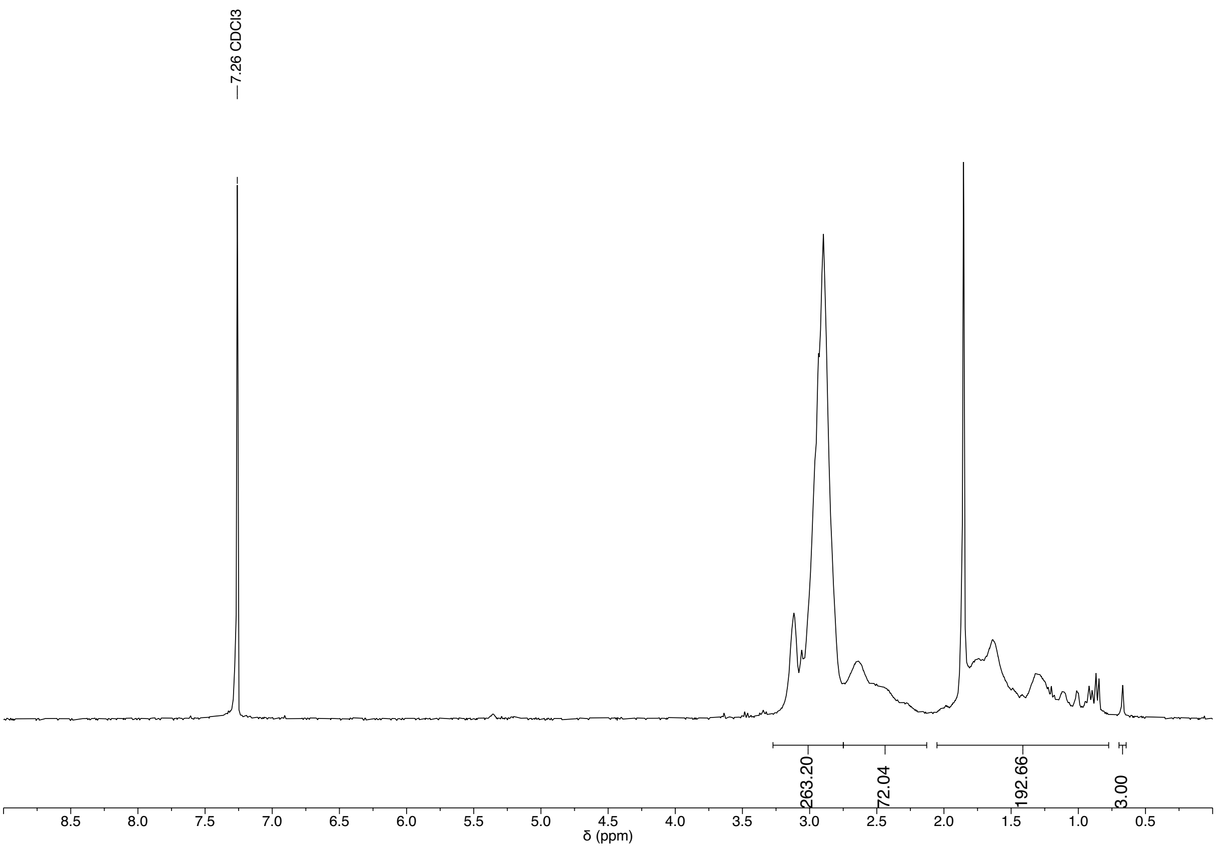

Figure S51: ^{1}H NMR (300 MHz, $CDCl_3$) of Chol-pDMA.

Chol-pDMA-SIL-TAMRA

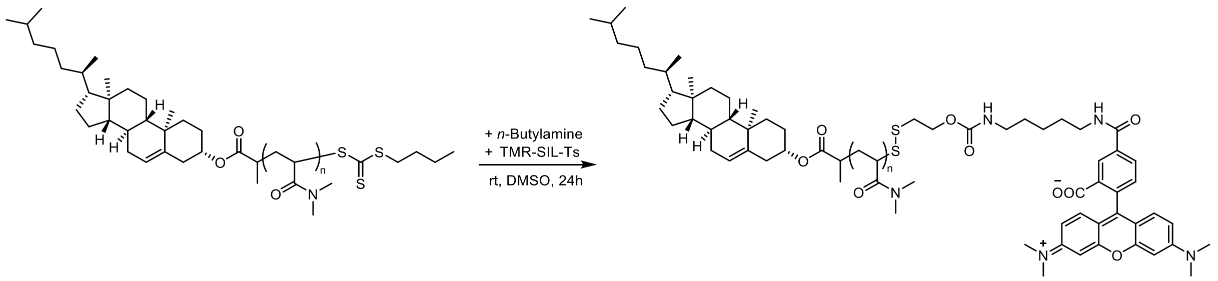

Chol-pDMA (5 mg, 0.9 µmol) was dissolved in dry DMSO and *n*-butylamine (0.5 µL, 5 µmol) as well as TAMRA-SIL-Ts (359.5 µL of 2.58 mg/mL stock-solution, 1.2 µmol) were added. The reaction was left to stir overnight. The next day, it was diluted with MilliQ water to a volume of 5 ml, dialysed against MilliQ water for three days and lyophilized overnight. The product was obtained after lyophilization as an amorphous pink solid in quantitative yields.

SEC (HFIP; PMMA standard): M_n = 11,474 g mol^{-1}; M_w = 13,605 g mol^{-1}; PDI = 1.18.

pDMA-*co*-RhoB

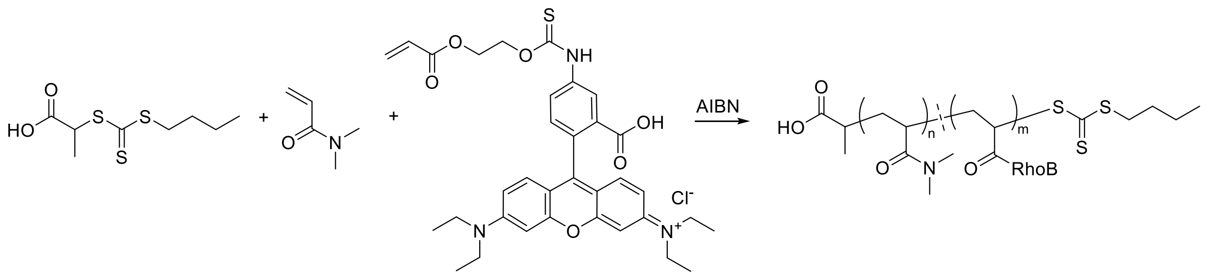

2-(butylthiocarbonothioylthio)propanoic acid (18.3 mg, 77 µmol), *N*,*N*-dimethylacrylamide (316 µL, 3067 µmol), AIBN (2.5 mg, 15 µmol) and acryloxyethyl thiocarbamoyl rhodamine B (2.5 mg, 4 µmol) were dissolved in 1.5 mL DMF. After three freeze-pump-thaw cycles, the reaction mixture was stirred for 6 h at 75 °C and subsequently repeatedly precipitated in cold ether.

^{1}H NMR (300 MHz, D_2O): δ [ppm] = 3.25 – 2.84 (m, 290H), 2.81 – 2.44 (m, 48H), 2.04 – 1.00 (m, 103H), 0.92 (t, 3H).

SEC (HFIP; PMMA standard): M_n = 6243 g mol^{-1}; M_w = 7223 g mol^{-1}; PDI = 1.16.

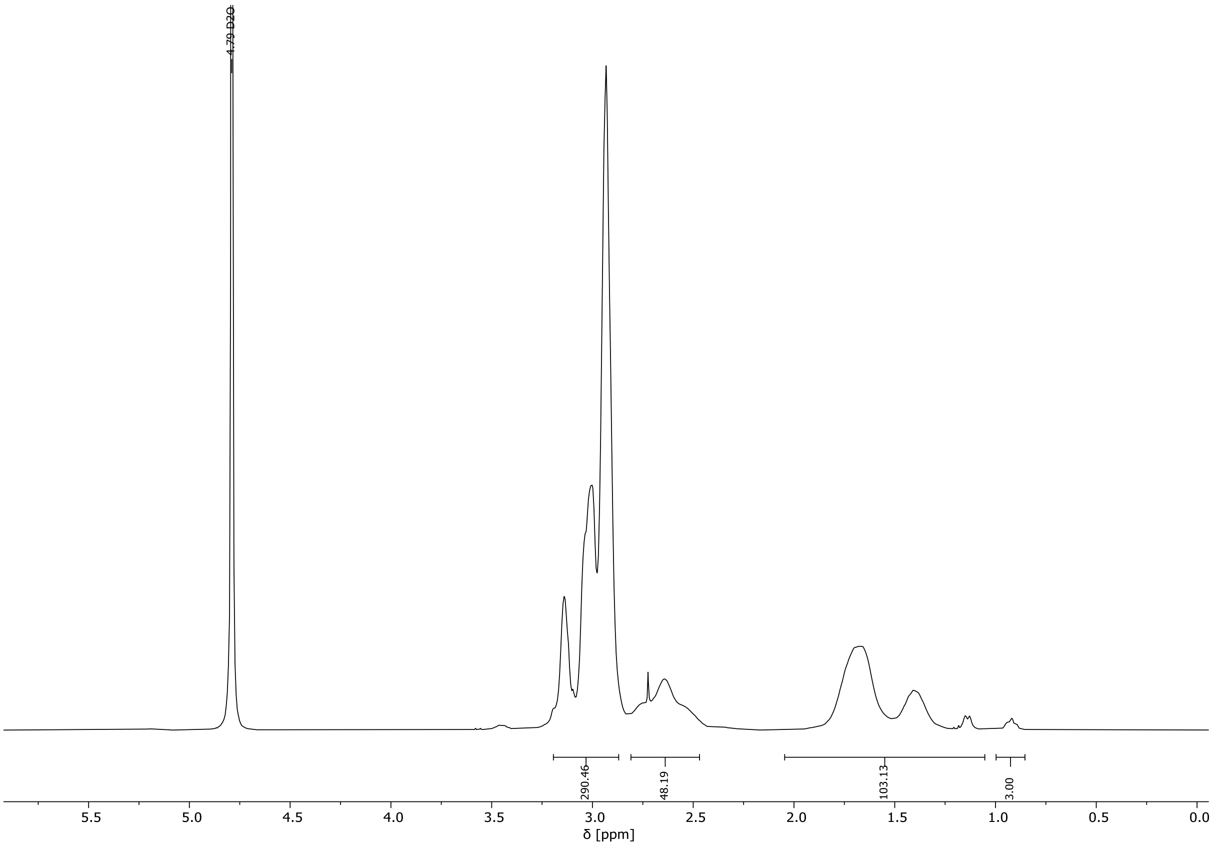

Figure S52: ^{1}H NMR (300 MHz, D_2O) of pDMA-*co*-RhoB.

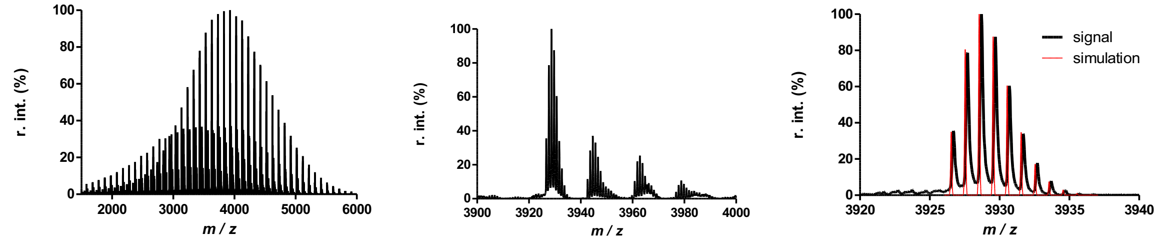

Figure S53: MALDI-ToF MS data of pDMA-*co*-RhoB. Full polymer mass range (left), zoomed mass range of DP with highest relative intensity (middle) and overlay of DP with highest relative intensity and its corresponding simulated isotope pattern in red (right).

pDMA-*co*-RhoB-SIL-IMDQ

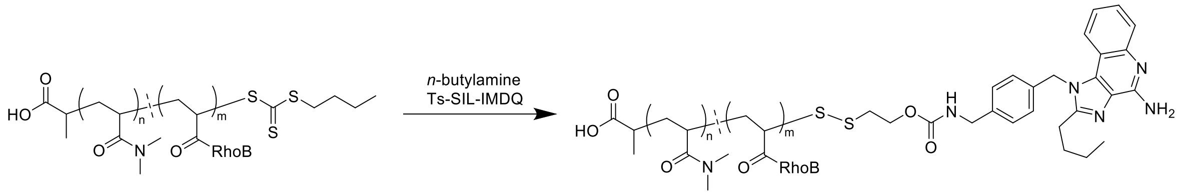

pDMA-*co*-RhoB (15 mg, 2.9 µmol) was dissolved in 158 µL degassed DMSO and 182 µL Ts-SIL-IMDQ (50 mg/mL in DMSO, 14.7 µmol) and *n*-butylamine (1.45 µL, 14.7 µmol) were added. The solution was stirred for 3 days at room temperature in the dark. Repeated dialysis against water gave the product after lyophilization as a pink solid.

SEC (HFIP; PMMA standard): M_n = 7360 g mol^{-1}; M_w = 8646 g mol^{-1}; PDI = 1.18.

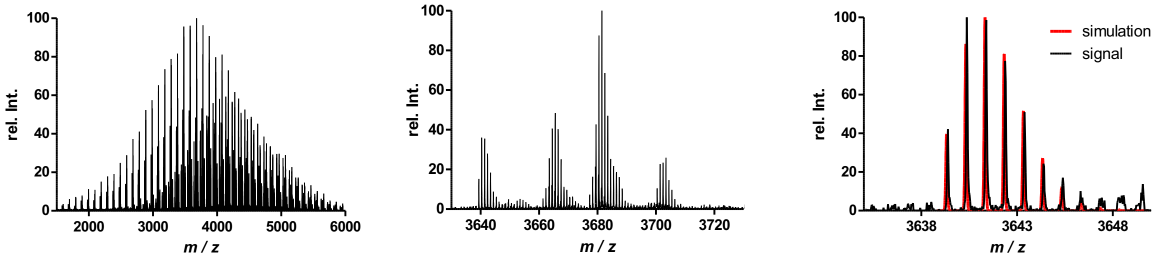

Figure S54: MALDI-ToF MS data of pDMA-*co*-RhoB-SIL-IMDQ. Full polymer mass range (left), zoomed mass range of DP with highest relative intensity (middle) and overlay of DP with highest relative intensity and its corresponding simulated isotope pattern in red (right).

pDMA-*co*-RhoB-mal-IMDQ

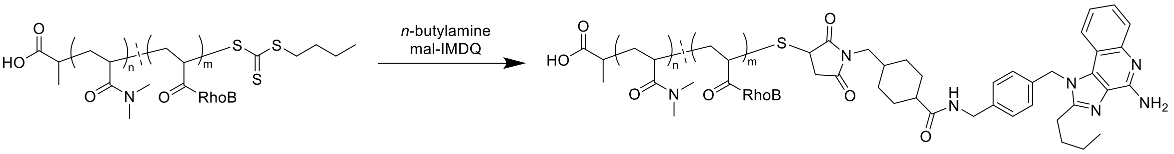

pDMA-*co*-RhoB (15 mg, 2.9 µmol) was dissolved in 500 µL degassed DMSO and *n*-butylamine (2.91 µL, 29.4 µmol) was added. The solution was stirred for 30 min and was rapidly precipitated in diethyl ether. The supernatant was removed and 340 µL mal-IMDQ (25 mg/mL in DMSO, 14.7 µmol) was added to the residue immediately under nitrogen atmosphere. The solution was stirred for 3 days at room temperature in the dark. Repeated dialysis against water gave the product after lyophilization as a pink solid.

SEC (HFIP; PMMA standard): M_n = 7583 g mol^{-1}; M_w = 9144 g mol^{-1}; PDI = 1.21.

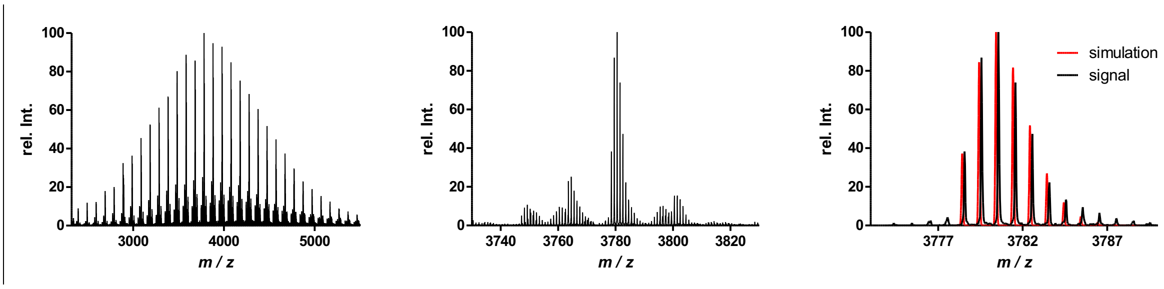

Figure S55: MALDI-ToF MS data of pDMA-*co*-RhoB-mal-IMDQ. Full polymer mass range (left), zoomed mass range of DP with highest relative intensity (middle) and overlay of DP with highest relative intensity and its corresponding simulated isotope pattern in red (right).

pDMA-*co*-RhoB-DMA

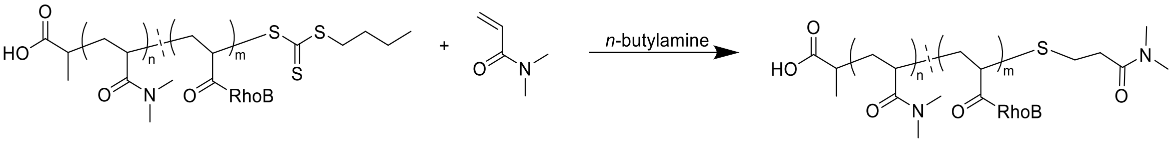

pDMA-*co*-RhoB (25 mg, 4.9 µmol), *n*-butylamine (3.64 µL, 36.8 µmol) and *N,N*-dimethylacrylamide (5.06 µL, 49.1 µmol) were dissolved in 0.5 mL degassed DMSO and the solution was stirred for three days at room temperature in the dark. Repeated dialysis against water gave the product after lyophilization as a pink solid.

SEC (HFIP; PMMA standard): M_n = 6465 g mol^{-1}; M_w = 7540 g mol^{-1}; PDI = 1.17.

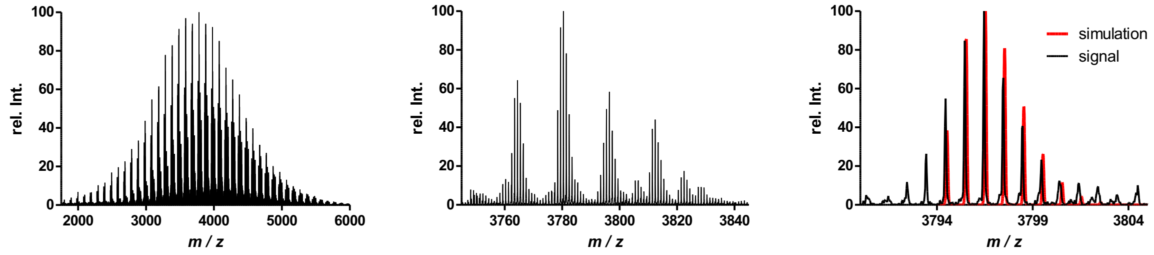

Figure S56: MALDI-ToF MS data of pDMA-*co*-RhoB-DMA. Full polymer mass range (left), zoomed mass range of DP with highest relative intensity (middle) and overlay of DP with highest relative intensity and its corresponding simulated isotope pattern in red (right).

Chol-pDMA-*co*-RhoB

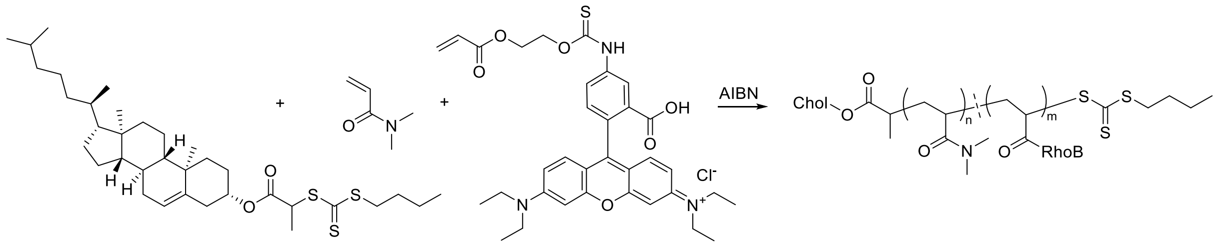

Cholesteryl 2-(((butylthio)carbonothioyl)thio)propanoate (30.4 mg, 50 µmol), *N*,*N*-dimethylacrylamide (154 µL, 1.5 mmol) and AIBN (1.7 mg, 10 µmol) were dissolved in 400 µL dioxane and acryloxyethyl thiocarbamoyl rhodamine B (1.7 mg, 2.5 µmol) in 400 µL DMF was added. After 5 freeze-pump-thaw cycles, the reaction mixture was stirred for 4.5 h at 75 °C and subsequently repeatedly precipitated in cold diethyl ether.

^{1}H NMR (300 MHz, $CDCl_3$): δ [ppm] = 3.23 – 2.71 (m, 258H), 2.68 – 2.27 (m, 53H), 1.98 – 0.79 (m, 145H), 0.67 (s, 3H).

SEC (HFIP; PMMA standard): M_n = 7126 g mol^{-1}; M_w = 7994 g mol^{-1}; PDI = 1.12.

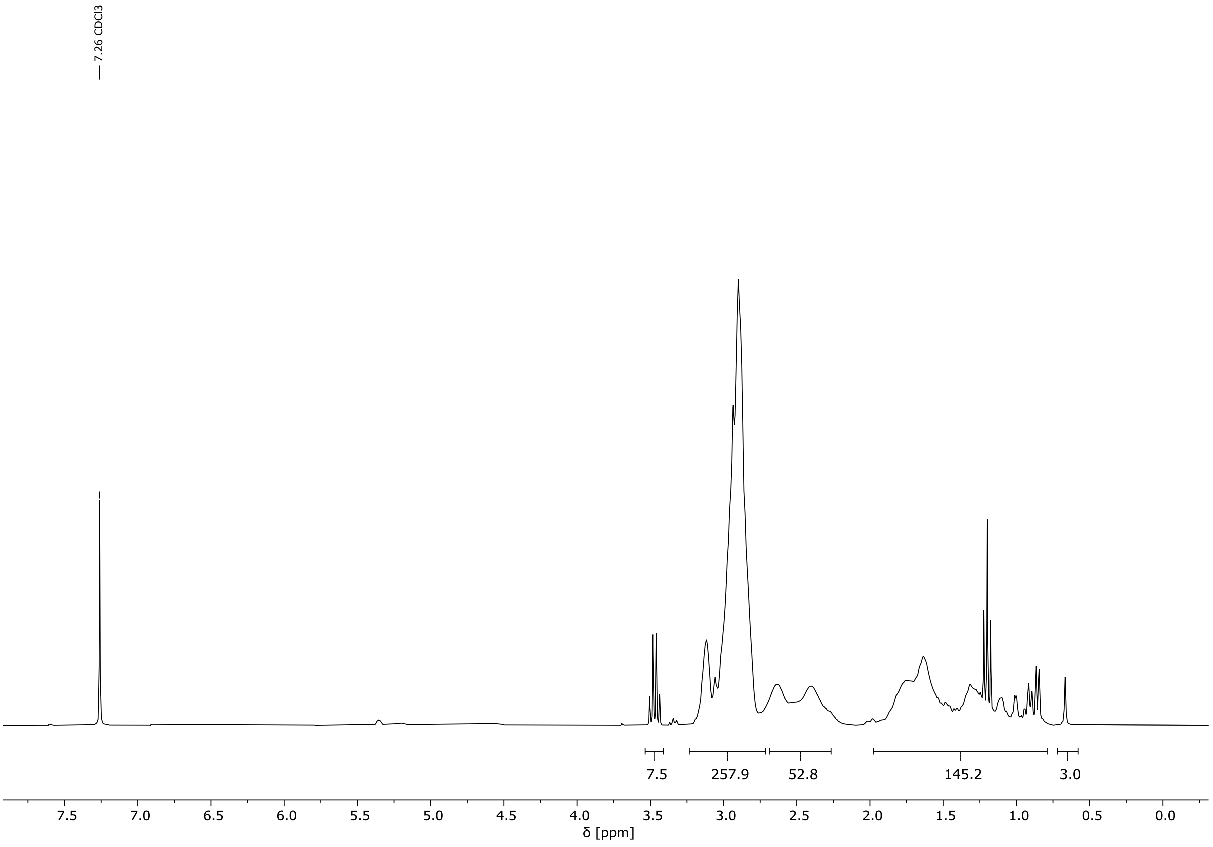

Figure S57: ^{1}H NMR (300 MHz, $CDCl_3$) of Chol-pDMA-*co*-RhoB.

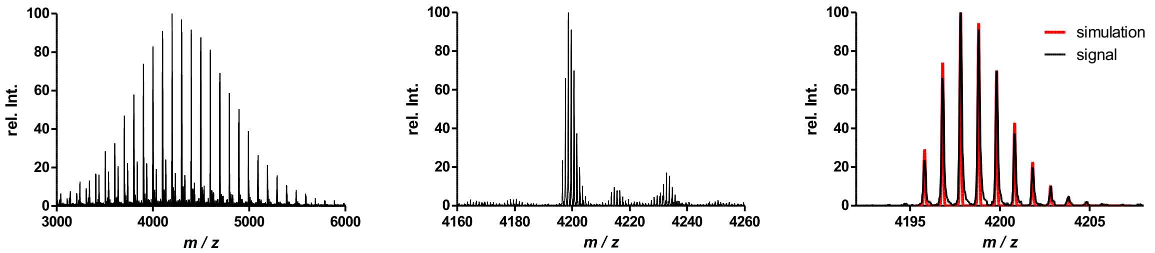

Figure S58: MALDI-ToF MS data of Chol-pDMA-*co*-RhoB. Full polymer mass range (left), zoomed mass range of DP with highest relative intensity (middle) and overlay of DP with highest relative intensity and its corresponding simulated isotope pattern in red (right).

Chol-pDMA-*co*-RhoB-SIL-IMDQ

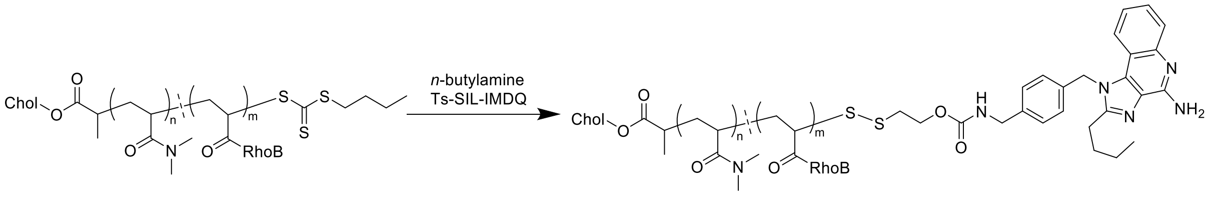

Chol-pDMA-*co*-RhoB (24 mg, 4.9 µmol) was dissolved in 362 µL Ts-SIL-IMDQ (50 mg/mL in DMSO, 29.2 µmol) and *n*-butylamine (3.61 µL, 36.5 µmol) was added and stirred for 5 days at room temperature in the dark. Dialysis against MeOH, MeOH/water = 1/1 and repeatedly against water gave the product after lyophilization as an amorphous pink solid.

SEC (HFIP; PMMA standard): M_n = 8946 g mol^{-1}; M_w = 10630 g mol^{-1}; PDI = 1.19.

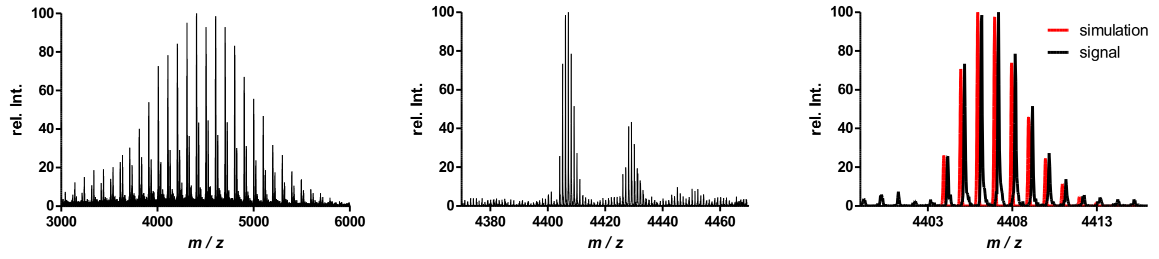

Figure S59: MALDI-ToF MS data of Chol-pDMA-*co*-RhoB-SIL-IMDQ. Full polymer mass range (left), zoomed mass range of DP with highest relative intensity (middle) and overlay of DP with highest relative intensity and its corresponding simulated isotope pattern in red (right).

Chol-pDMA-*co*-RhoB-DMA

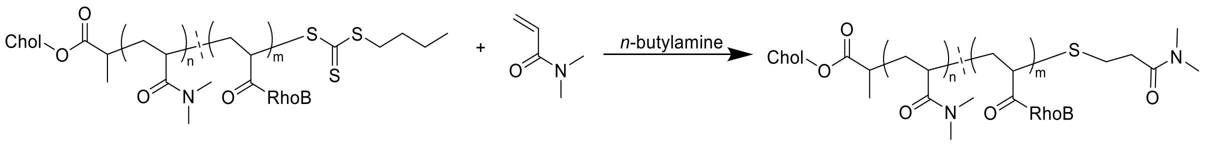

Chol-pDMA-*co*-RhoB (24 mg, 4.9 µmol), *n*-butylamine (5.42 µL, 54.8 µmol) and *N,N*-dimethylacrylamide (7.53 µL, 73.1 µmol) were dissolved in 0.5 mL degassed DMSO and the solution was stirred for three days at room temperature in the dark. Subsequent dialysis against MeOH, MeOH/water = 1/1 and repeatedly against water gave the product after lyophilization as an amorphous pink solid.

SEC (HFIP; PMMA standard): M_n = 7169 g mol^{-1}; M_w = 8118 g mol^{-1}; PDI = 1.13.

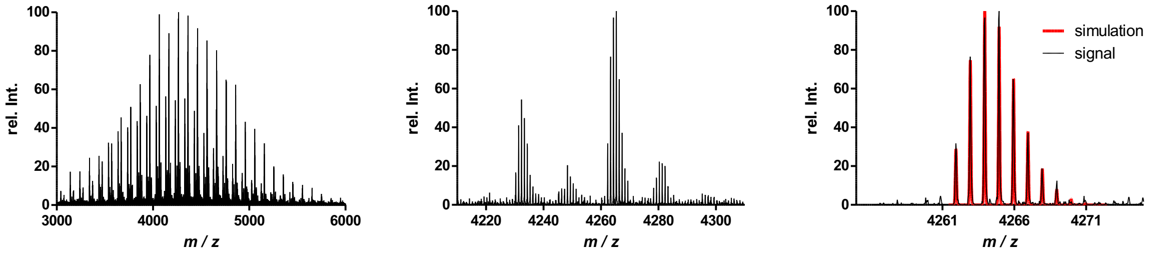

Figure S60: MALDI-ToF MS data of Chol-pDMA-*co*-RhoB-DMA. Full polymer mass range (left), zoomed mass range of DP with highest relative intensity (middle) and overlay of DP with highest relative intensity and its corresponding simulated isotope pattern in red (right).

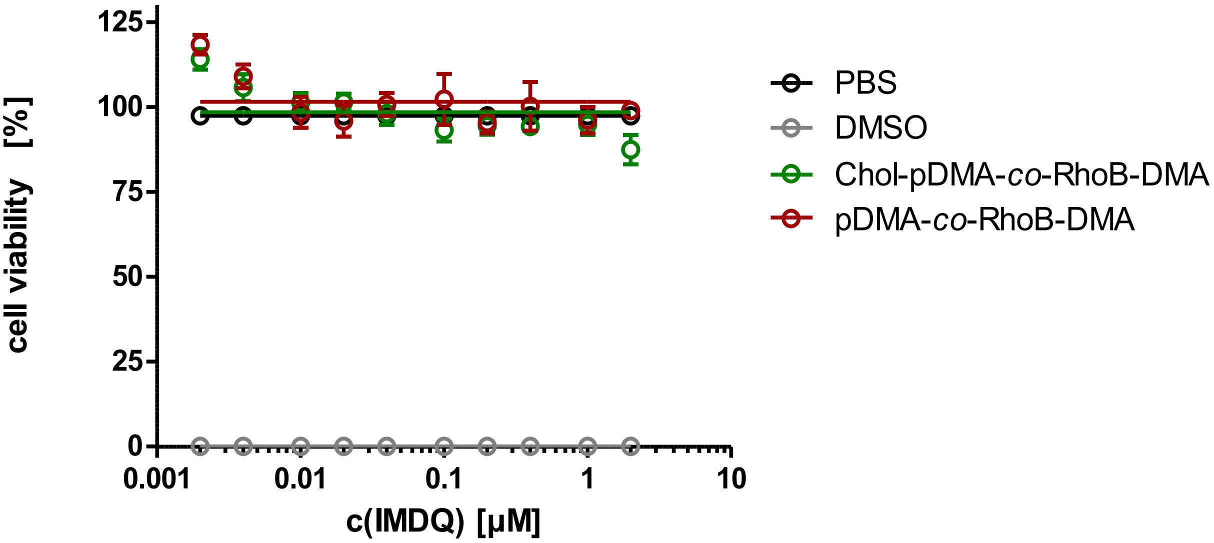

Figure S61: Cell viability assay of fluorescently labelled pDMA-*co*-RhoB-DMA and Chol-pDMA-*co*-RhoB-DMA.

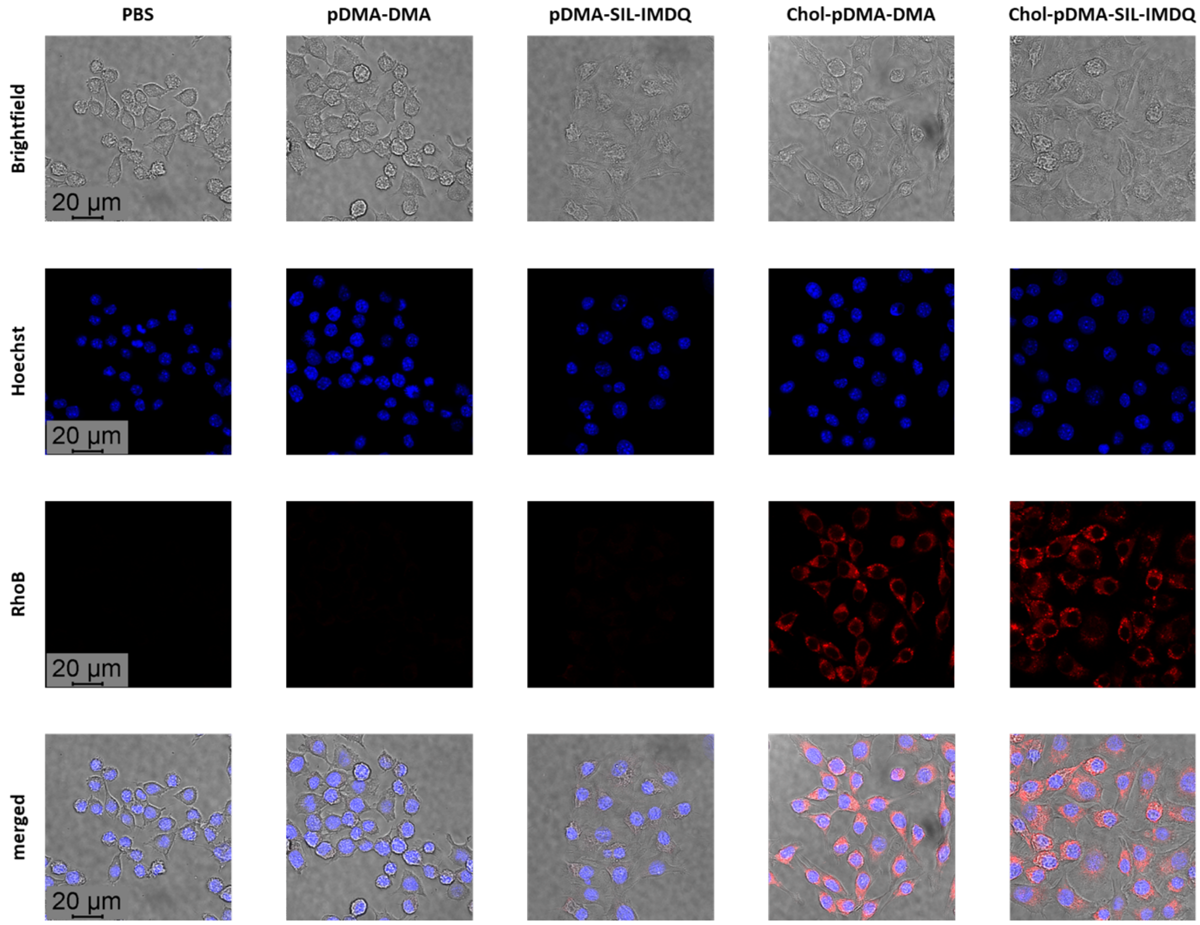

Figure S62: Confocal microscopy images of cells treated with PBS or rhodamine labelled pDMA-DMA, pDMA-SIL-IMDQ, Chol-pDMA-DMA and Chol-pDMA-SIL-IMDQ.

References:

[1] Scherger, M.; Räder, H. J.; Nuhn L. Self-Immolative RAFT-Polymer End Group Modification. *Macromol. Rapid Commun.* **2021**, *42*, 2000752.

[2] Jones, L. R.; Goun, E. A.; Shinde, R.; Rothbard, J. B.; Contag, C. H.; Wender, P. A. Releasable Luciferin−Transporter Conjugates: Tools for the Real-Time Analysis of Cellular Uptake and Release. *J. Am. Chem. Soc.* **2006**, *128*, 6526–6527.

[3] Bolli, E.; Scherger, M.; Arnouk, S. M.; Pombo Antunes, A. R.; Straßburger, D.; Urschbach, M.; Stickdorn, J.; De Vlaminck, K.; Movahedi, K.; Räder, H. J.; Hernot, S.; Besenius, P.; Van Ginderachter, J. A.; Nuhn, L. Targeted Repolarization of Tumor-Associated Macrophages via Imidazoquinoline-Linked Nanobodies. *Adv. Sci.* **2021**, *8*, 2004574.
